# Statistical modeling of RNA structure profiling experiments enables parsimonious reconstruction of structure landscapes

**DOI:** 10.1038/s41467-018-02923-8

**Published:** 2018-02-09

**Authors:** Hua Li, Sharon Aviran

**Affiliations:** Department of Biomedical Engineering and Genome Center, University of California at Davis, Davis, CA 95616 USA

## Abstract

RNA plays key regulatory roles in diverse cellular processes, where its functionality often derives from folding into and converting between structures. Many RNAs further rely on co-existence of alternative structures, which govern their response to cellular signals. However, characterizing heterogeneous landscapes is difficult, both experimentally and computationally. Recently, structure profiling experiments have emerged as powerful and affordable structure characterization methods, which improve computational structure prediction. To date, efforts have centered on predicting one optimal structure, with much less progress made on multiple-structure prediction. Here, we report a probabilistic modeling approach that predicts a parsimonious set of co-existing structures and estimates their abundances from structure profiling data. We demonstrate robust landscape reconstruction and quantitative insights into structural dynamics by analyzing numerous data sets. This work establishes a framework for data-directed characterization of structure landscapes to aid experimentalists in performing structure-function studies.

## Abstract (web summary)

Different experimental and computational approaches can be used to study RNA structures. Here, the authors present a computational method for data-directed reconstruction of complex RNA structure landscapes, which predicts a parsimonious set of co-existing structures and estimates their abundances from structure profiling data.

## Introduction

The diverse regulatory functions of RNA are deeply rooted in its ability to form and switch between specific structures, thus making structure prediction an essential task in biology and biotechnology^1^. Modeling RNA structure accurately, however, is a difficult task, both experimentally and computationally^2^. Of particular interest and challenge are regulatory systems that involve multiple functional and biologically important structures, such as natural and engineered riboswitches, riboSNitches, ribozymes, thermosensors, viral elements, protein-bound RNA, and folding pathways^3–10^. Often, these alternative structures are jointly present at different abundances, to confer these systems their flexibility and sensitivity in responding to diverse cellular signals. Accurate characterization of these complex and dynamic structure landscapes is thus critical to understanding and engineering riboregulators. However, despite steady progress in experimentally probing complex landscapes with nuclear magnetic resonance (NMR), single-particle, and single-molecule techniques, these approaches still suffer technological, infrastructure, size, and resolution limitations that prohibit their broad application^6,8,11,12^.

**Fig. 1:**
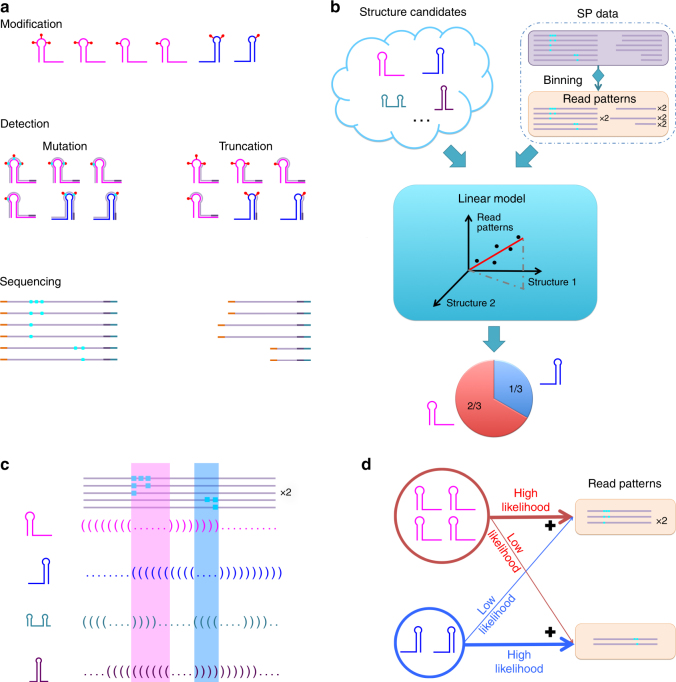
Cartoon depictions of structure profiling experiments, SLEQ’s workflow, and its modeling principles. **a** RNA molecules adopt multiple co-existing structures of potentially differing abundances (magenta and blue hairpins). Structures are probed with a reagent that preferentially modifies structurally unconstrained nucleotides. Modifications (red pins) are detected by reverse transcriptase, which either stops or introduces mutations at modified sites. Complementary DNA products are sequenced and reads are classified by modification locations, from which degrees of modification are quantified. **b** Workflow of SLEQ. Sequencing reads and candidate structures are input to SLEQ. Reads report detections via truncation or mutation (purple box) and are subsequently binned into patterns (yellow box). See Supplementary Fig. 1 for detailed binning routine. At the core of SLEQ is a linear model that links input structures to observed patterns. Through linear parameter estimation, a small set of structures is selected and their abundances are estimated. **c** SLEQ selects structures that jointly support observed read patterns. Candidate structures, shown in dot-bracket format, are aligned to read patterns. Modifications occur predominantly in unconstrained nucleotides (dots). Top three patterns are supported by the magenta structure, whereas the bottom two are consistent with the blue structure (background colors highlight loop locations). Green and purple structures are unlikely to generate shown patterns. These principles drive SLEQ to select magenta and blue structures. **d** Conceptual model description. Each observed pattern may be generated by any underlying structure with a structure-dependent likelihood. The structure-to-pattern likelihood is determined by the degree of consistency between modifications in a pattern and regions of unconstrained nucleotides in the structure. As indicated in **c**, the magenta structure is highly likely to generate patterns in upper yellow box but unlikely to generate patterns in lower yellow box, and vice versa for the blue structure. Subpopulations of candidate structures then additively contribute to observed pattern counts. Individual subpopulation contributions are determined by their relative abundance and pattern likelihood

Chemical structure profiling (SP) experiments have recently emerged as affordable and powerful methods for analyzing RNA structures in vivo, in vitro, and in a massively parallel fashion. Propelled by advances in DNA sequencing, this classical approach has dramatically expanded both in scope and depth, unveiling a complex layer of RNA-based regulation^13–16^. In SP, local structural characteristics are gleaned using structure-sensitive reagents, such as SHAPE and DMS, which preferentially modify structurally unconstrained nucleotides (Fig. 1a). Modifications are detected via reverse transcription, which either stops at modified nucleotides or proceeds while introducing a mutation. Resulting complementary DNA (cDNA) products are sequenced and stops/mutations are tallied to determine modification frequencies per nucleotide. Progress in experiments has also spurred development of computational tools that use SP data to either constrain secondary structure prediction algorithms or, more recently, to inform data-driven discovery in a range of emerging applications^17–19^.

Harnessing SP data to better predict structure computationally proved widely successful^20,21^, although efforts have centered on predicting a single secondary structure—typically the most thermodynamically stable. Less progress has been made on predicting multiple structures in situations where their joint presence is critical for function, primarily due to computational challenges. One key difficulty stems from the fact that SP measurements capture an ensemble-weighted average over the different structures^17,18^. Other than M^2^-REEFFIT and RING-MaP^22,23^, current approaches entail large-scale data-directed sampling of suboptimal secondary structures, which interprets SP data as the signature of one structure that dominates in solution^17^. Furthermore, generated secondary structures are often subject to limitations of thermodynamic models^21^ and also warrant lossy post-processing via clustering and/or dimensionality reduction to mine a simplified view of key structures^24–26^. While this paper was under review, another group reported a thermodynamics-based method, Rsample, which employs data-directed suboptimal sampling followed by large-scale clustering, yet it explicitly considers multiple structures as the sources of the data^27^. Rsample predicts multiple structures and their respective population fractions. Notably, M^2^-REEFFIT and RING-MaP rely on different large-scale clustering techniques, which extract information on dominant motifs (e.g., helices or local tertiary interactions) or on constituent structure profiles. Yet, to our knowledge, they do not directly output complete structures along with their respective population fractions. M^2^-REEFFIT is also data-intensive, warranting two-dimensional data sets comprising of *M* + 1 structure profiles for an RNA of length *M*, which can be currently obtained only in vitro. RING-MaP is specialized to a single SP technique and further requires ultra-high modification rates. Methods of broad applicability, which provide direct and compact reconstruction of complex ensemble dynamics are currently lacking.

Here, we introduce structure landscape explorer and quantifier (SLEQ), a method for sparse SP-guided reconstruction of RNA structure landscapes. SLEQ features a statistical model that leverages prior modeling work^28,29^ and “sample and select” principles^30^ to consider large structural ensembles and to interpret SP data as an aggregate (or average) measure of structure-specific modifications^17,25^. It selects a parsimonious set of structures that best explain SP data from a pre-determined candidate set and estimates their relative abundances. Such parsimony is critical to elucidating complex dynamics as it identifies dominant structures, which likely govern function. SLEQ is versatile in two key ways: (1) it accommodates complex structural features, which allow it to circumvent limitations of conventional structure prediction algorithms; and (2) it supports data obtained by two prominent SP paradigms—mutation and truncation, and thus applies to diverse data sets. Its versatility and succinct output render it a broadly applicable and accessible framework for quantitative studies of complex RNA dynamics. To demonstrate its performance, utility, and breadth, we analyze several systems whose function critically derives from complex structure landscapes.

## Results

### Statistical model and inference

SLEQ takes two inputs: a set of candidate structures and SP sequencing data (Fig. 1b). Candidate structures are obtained computationally and/or manually and represent prior knowledge of structural dynamics. Each sequencing read reports a chemical modification pattern detected in a single molecule (Methods). Two main approaches to modification detection exist: truncating the transcribed cDNA at the modification site or introducing a mutation at that site^13,31^. They differ in the set of patterns they detect (Fig. 1a), with mutational profiling being capable of assaying a richer and potentially more informative pattern set. SLEQ thus supports two modes: mutation and truncation, where reads are binned into the appropriate set of patterns (see Methods and Supplementary Fig. 1). A linear model then links observed pattern statistics to the unknown structural sample composition. By implementing non-negative least-squares model fitting, SLEQ selects a parsimonious subset of candidate structures that best explain observed modifications and jointly estimates these structures’ abundances. See Methods for details.

Figure 1c, d highlights the theoretical ideas and concepts that underlie our method. SLEQ’s reconstruction of the landscape is driven by the degree of consistency between each candidate structure and the modification patterns observed in reads. Since modifications are expected to occur predominantly in unconstrained or unpaired nucleotides, a structure is deemed consistent with a pattern if nucleotides modified in said pattern correspond to some of its unconstrained regions. For example, two regions of modifications are highlighted in Fig. 1c. Alignment of reads with dot-bracket representations of candidate structures indicates that the top hairpin (magenta) is consistent with the top three patterns and the second hairpin (blue) is consistent with the other two patterns. The other two candidate structures are inconsistent with the reads shown.

To quantify structure-to-pattern consistency, SLEQ employs a probabilistic model. Extending on prior models that link average modification frequencies in a sample to observed reads^28,29^, here we consider a heterogeneous population of explicit structures as the generative source of reads. Furthermore, we model the statistical relationship between any given structure and any possible read pattern, where modeling at the read resolution is key to SLEQ’s ability to unify treatment of truncation and mutation data sets. As structurally unconstrained nucleotides can potentially be modified, for each candidate structure, the model considers its nucleotide states (constrained/unconstrained) to calculate the likelihood that it would generate each of the observed reads (Fig. 1d). As SLEQ seeks a subset of structures that jointly support the data, our probabilistic model is embedded within a linear model, which captures the understanding that co-existing populations of distinct structures additively contribute to a total number of reads observed for each pattern. The linear model thus accounts for the relative abundances of these populations, which are unknown and estimated by fitting the model to the pattern counts. A particular advantage of linear modeling is the availability of well-established techniques for sparse optimization, such that a solution entailing a small subset of candidate structures can be found. See Methods for details.

### SLEQ recovers structural heterogeneity of a human riboSNitch

The recently developed DMS-MaPseq technique couples mutational profiling with DMS chemistry and attains modification densities substantially higher than previously feasible despite the fact that modifications are assayed only for adenines and cytosines^32^. One advantage of mutational profiling lies in its ability to resolve allelic origins of reads, as its cDNA products entail more complete sequence information than truncated cDNAs^31^. Such information becomes crucial when studying riboSNitches—single-nucleotide polymorphisms (SNPs) that result in local structural re-arrangement, which may have phenotypic outcomes^4^. In ref. ^32^, structural heterogeneity associated with the human *MRPS21* riboSNitch was revealed by DMS-MaPseq. Samples transcribed in vitro from two *MRPS21* alleles were mixed and profiled. Allele-specific data analysis uncovered highly distinct structure profiles, which were no longer separable from the mixed-data profile (Supplementary Fig. 2). This proof-of-principle is especially suited for method validation for several reasons: (1) constituent structural signatures were undetectable in mixture; (2) sample was sequenced deeply; and (3) SNP identity (A/C) within reads enabled recovery of true allelic composition for benchmarking.

**Fig. 2:**
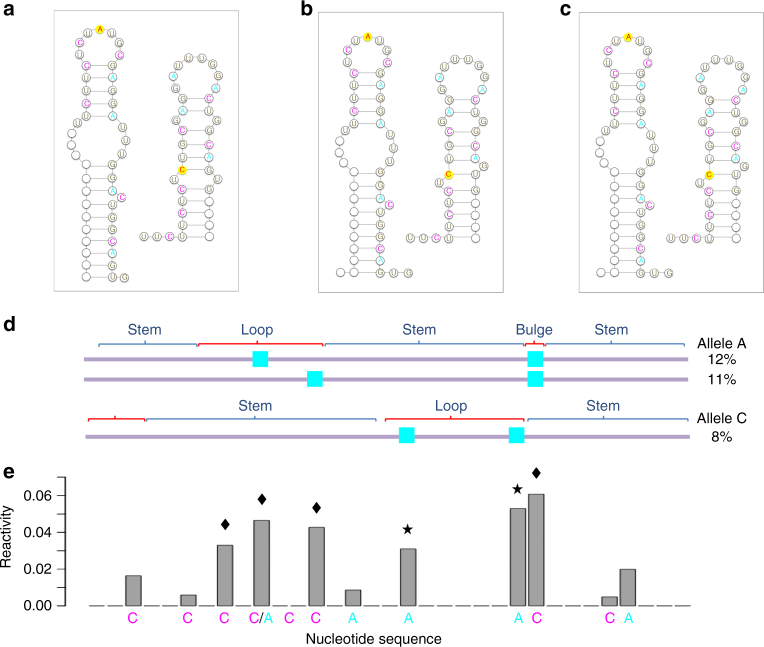
Structure heterogeneity of the *MRPS21* riboSNitch. **a** Reference structures of alleles A and C as represented in ref. ^32^. Representatives of candidate structures selected by SLEQ in mutation mode **b** and truncation mode **c**. Nucleotides marked by empty circles are found in regions of no overlap between the reference structures shown in ref. ^32^ and hence excluded from analysis. Data points with DMS information are highlighted in cyan and magenta for adenines and cytosines, respectively. Red letter over yellow background indicates SNP site. **d** Top three double-mutation data patterns are shown with their relative frequencies. The top two map to allele A and the third maps to allele C. A general sketch of the reference structure for each allele is shown on top of the patterns to help visualize the agreement between patterns and structures, which drives the selection of similar structures. The higher frequency of patterns supporting A drives the estimation of its higher abundance in the reconstructed ensemble. **e** Reactivity profile of the heterogeneous sample with both alleles. Profile is aligned with the sequencing reads shown in **d** and nucleotide labels and colors match the ones shown in **a**. One can see that average ensemble-based data does not fit individual structures well. For example, the structure selected for allele A does not support the highly reactive adenines marked by stars, which reside within a stem. Similarly, the structure selected for allele C does not support the highly reactive cytosines marked by diamonds

We tested SLEQ’s ability to recover the predicted allelic structures and estimate their abundances from mixture data (Fig. 2a–c). Analysis of >56 million reads revealed the ground truth to be 67.8%:32.2% for A:C (Supplementary Methods). We statistically sampled 1000 secondary structures for each allele sequence, removed duplicates, and combined samples into one set of >100 structures. To account for inter-sample variation at the base-pair level, which is characteristic of statistical samples^24,26^, we considered 10 independent samples in all analyses. Although DMS-MaPseq can report multiple modifications per read by way of mutational profiling, it is also possible to generate an equivalent truncation data set in silico, where each read reports up to one modification (see Methods for details and Supplementary Fig. 1 for illustration). By summarizing the sequencing data in these two forms, we were able to apply SLEQ in both truncation and mutation modes to gauge the impact of their differing information contents on its performance.

In mutation mode, a representative run of SLEQ selected only four structures with abundances exceeding 5%, populating 98% of the sample (estimates were 48, 14, 8, and 30%). Due to minor discrepancies between selected structures and structures shown in ref. ^32^, we clustered them into A/C structure classes. Each cluster comprised of two structures with total abundances 62%:38% (A:C). Figure 2a, b shows the reference structures and a structure representative for each cluster, respectively. Representatives were selected in two steps. First, we identified the most abundant structure in the cluster. Second, we locally minimized free energy of structures by retrieving all candidates whose pairing states at all probed adenines and cytosines were identical to their states in the selected structure. Of these, the structure with minimum free energy was chosen as representative. This strategy accounts for SLEQ’s data-directed principles, as these structures diverge in regions where no DMS information is available to guide SLEQ and are thus deemed comparable by SLEQ’s model despite their energy differences. Supplementary Table 1 summarizes our findings over 10 runs, indicating reproducible results with average A:C estimates 59%:41% and 4% standard deviation. Numbers of selected structures ranged from 4 to 6 and all were in complete or near-complete agreement with reference structures (on average, one nucleotide differed in pairing state). Notably, the best-performing run estimated abundances at 64%:36% (compare to ground truth 68%:32%).

To gain insight into how the data drives SLEQ’s reconstructions, we inspected prevalent read patterns. Figure 2d shows the three most frequent patterns of two mutations. The two top patterns map to allele A, and one can readily see that they are consistent with its reference structure but inconsistent with allele C’s reference structure. Together, these patterns are also significantly more prevalent than the third pattern, which maps to allele C and is consistent with its reference structure but inconsistent with reference structure A. The discrepancies in A/C pattern frequencies manifest in an ensemble composition that favors allele A. Furthermore, inspection of the reactivity profile of the mixed sample (Fig. 2e) revealed that measurements indeed capture ensemble averages, as modeled by SLEQ (Fig. 1d). One can see that neither individual structure selected by SLEQ solely supports the entirety of the reactivity profile, hence a heterogeneous solution was obtained.

Next, we applied SLEQ to the equivalent data set of in silico-generated truncated reads. Results were nearly identical, especially in terms of numbers of structures and their discrepancies from references. Average A:C estimates 62%:38 and 2% standard deviation indicated slight improvement, albeit not highly significant (paired *t* test, *p* value 0.067). Selected structures from a representative run are shown in Fig. 2c. These results demonstrate successful and robust landscape reconstruction, with minor differences between modes. In this particular example, truncation data appears to be sufficiently informative with respect to landscape reconstruction, such that reads with one modification carry the information needed to accurately resolve the mixture (see also Fig. 2e and Supplementary Fig. 2). Moreover, mutation data would embed more information than truncation data only if many reads had contained more than one modification. In this case, however, <4% of the reads belong to this category (see Methods for pattern statistics), which deems insufficient. Note, however, that such low frequency does not imply low modification rate because mutations were considered at 11 adenines/cytosines only (Fig. 2e). In fact, the modification rate is ~1 per 33 A/C nucleotides, an order of magnitude higher than previous standards in the field^33^. Given the exponential growth in multiple-modification patterns with increasing RNA length, their frequency would rapidly grow upon consideration of longer regions and may thereby confer mutational profiling access to additional valuable information.

### SLEQ quantitatively elucidates riboswitch folding dynamics

To further validate SLEQ’s capacity to uncover and quantify structural subpopulations, we turned to more complex dynamics. In ref. ^34^, in vitro RNA polymerase arrest was coupled to SHAPE-Seq to monitor cotranscriptional folding of *Bacillus cereus crcB* fluoride riboswitch in presence and absence of fluoride. Briefly, transcription roadblocks embedded into DNA templates capture folding intermediates, which are subsequently profiled (see Fig. 1 in ref. ^34^). Watters et al.^34^ visually inspected consecutive SHAPE-Seq profiles of increasing length to infer folding trajectories with/without fluoride, based on which they proposed a mechanistic model to elucidate how the riboswitch is directed into each of two previously characterized ligand-bound/unbound states^35,36^. Yet, crafting such model warrants manual inspection and interpretation of complex data sets, relying substantially on qualitative comparisons at select few nucleotides and user-guided structure modeling (see Figs. 4–6 in ref. ^34^).

**Fig. 3:**
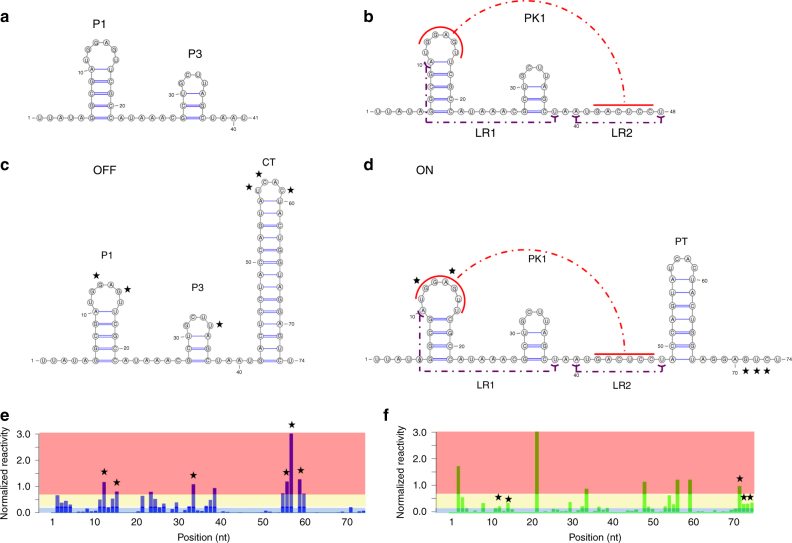
Structures that govern cotranscriptional folding of *crcB* fluoride riboswitch and reactivity profiles after formation of a complete terminator (CT). **a**,** b** Dominant motifs P1, P3, and PK1 form at intermediate transcript lengths 26, 41, and 49 nt independently of fluoride presence. **c**, **d** At sufficient transcript length, fluoride-dependent structural bifurcation takes place. In the absence of fluoride, the OFF structure **c** dominates whereas fluoride binding results in the emergence of an ON structure **d**. **e**, **f** Reactivity profiles matching the transcript length shown in **c**, **d** without **e** and with **f** fluoride. Reactivities were normalized by the 2–8% strategy^21^. Background colors indicate ranges of high (red)/medium (yellow)/low (blue) reactivities. In the absence of fluoride, SLEQ’s reconstruction indicates that the OFF structure dominates the ensemble. The profile in **e** reveals strong signals (highlighted by stars) from loops in the P1 and CT hairpins, which agree with the OFF structure. However, the ON structure does not support a P1 loop, as indicated by decreased reactivity in this region (see **f**). In the presence of fluoride, SLEQ reconstructed a heterogeneous ensemble that is well balanced between ON and OFF. The profile in **f** shows intermediate reactivities at the 3′ end, supporting the presence of an ON structure. However, reactivites at the 3′ end and at P1 loop regions (highlighted by stars) are in the intermediate range, implying that the average pairing states of nucleotides in these regions are neither fully constrained nor fully unconstrained. This suggests that these data are best supported by a mixture of ON and OFF. Note that despite noticeable differences in ON and OFF structures, regions of pairing-state differences are limited to P1 loop (nucleotides 12–16) and the region immediately following PT (nucleotides 67–73)

We used SLEQ to explore this folding trajectory quantitatively. Consistent with prior work^34,37^, our analysis distinguishes between two phases: ligand-independent and ligand-dependent. In the ligand-independent phase, two hairpins are folded sequentially—P1 and P3 (Fig. 3a). Details of SLEQ’s reconstruction of P1 and P3 formation at two intermediate lengths are found in Supplementary Methods. Their folding sets the stage for the formation of pseudoknot PK1 (Fig. 3b). This initial phase is followed by ligand-dependent bifurcation when OFF (terminated) and ON (antiterminated) states emerge (see Fig. 3c, d, Supplementary Fig. 10 in ref. ^36^ and Supplementary Fig. 10 in ref. ^35^). Note that differences between ON and OFF amount to helix formation between P1 loop and nucleotides 42–47 (pseudoknot PK1) and two long-range (LR) interactions (A10-U38, A40-U48). Previous studies thus suggested that fluoride activates transcription by preventing formation of a complete terminator (CT), as mediated by PK1 formation and stabilization at the aptamer domain^35–37^. Support for these ON–OFF differences can also be gleaned from reactivity profiles in the absence and presence of fluoride (Fig. 3e, f). Without fluoride, P1 loop is reactive, consistent with the OFF state. With fluoride, reactivity in this region decreases to intermediate levels, supporting the presence of ON structure in the sample.

To explore ligand-dependent dynamics, we analyzed profile lengths 55–80 nt. Since conventional statistical sampling algorithms disregard pseudoknoted structures^24^, we spiked six PK1-containing structures into candidate sets (Supplementary Methods). SLEQ’s output structures and abundance trajectories revealed several interesting findings. As expected, the ON structure (PK1 and partial terminator PT) is consistently and substantially more abundant in fluoride’s presence than in its absence (Supplementary Fig. 3, solid purple and dashed green lines). A similar trend is seen upon inspection of the total fraction of structures that contain PK1 but not necessarily in tandem with PT (Supplementary Fig. 3, solid red and dashed blue lines). This population was obtained by manually clustering the small number of structures output by SLEQ. It is of interest because PK1 suffices to prohibit terminator formation (Fig. 3c, d) and is thus key to ON structure’s function in gene regulation^35,36^. Moreover, without fluoride, ON structure disappears at 71 nt and similarly for the structures containing PK1 (76 nt). These dynamics support previous insights into the ligand’s role in stabilizing the aptamer domain^34,36^.

SLEQ outputs are best visualized by pairing probabilities plots (PPPs). For example, Supplementary Fig. 4 shows outputs at 69 nt with/without fluoride. The OFF structure dominates without fluoride (see P1, P3, terminator helices), whereas ON dominates with fluoride (see P1, P3, PT, PK1 helices and LR). Ovals highlight substantially elevated PK1 and LR presence (note yellow-to-red transition). Prevention of CT formation in favor of aptamer interactions can be seen from base-pair colors at lower-right corner.

**Fig. 4:**
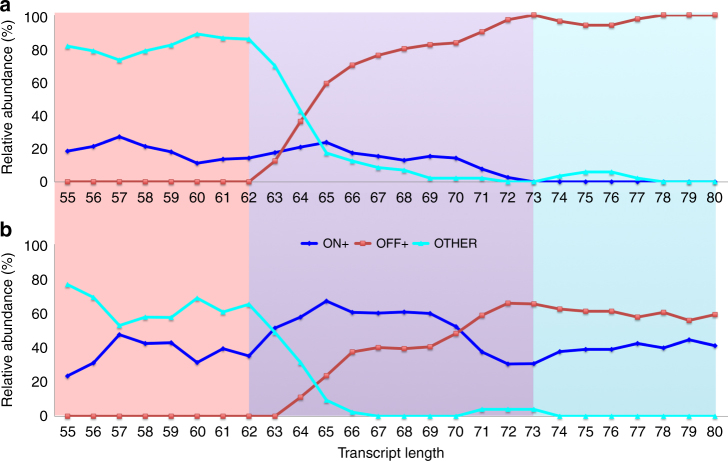
Quantitative analysis of the cotranscriptional structure landscape of *crcB* fluoride riboswitch based on SLEQ outputs. Relative abundance trajectories of ON+, OFF+, and OTHER clusters in the absence **a** and presence **b** of fluoride, as dependent on intermediate transcript length. The structures output by SLEQ were manually assigned into clusters according to whether they feature certain motifs that are key to ON and OFF structures functionality. The ON+ cluster includes structures that contain PK1. The OFF+ cluster includes structures containing P1 and the forming CT

From abundance trajectories, three subphases emerged, defined by two events: formation of terminator helices starting at *t*_start_ (~62 nt) and completion of CT at *t*_end_ (~73 nt). To illustrate this, Fig. 4a, b depicts population fractions of three classes: ON+, OFF+, and OTHER (Supplementary Table 2). Classes were obtained by manually assigning the small number of structures selected by SLEQ into three clusters, which we defined from a functional perspective. Particularly, based on prior studies^35^, we identified motifs within the ON and OFF structures (Fig. 3c, d), which are key to their function with and without fluoride, respectively. As mentioned above, the key motif to ON structure’s functionality is PK1, hence class ON+ comprises of all structures that feature PK1 (see Supplementary Fig. 5 for examples). Similarly, OFF+ class comprises of structures with both P1 and terminator (see Supplementary Fig. 6 for examples), whereas OTHER consists of the remaining structures. Without ligand, OFF+ population upsurges at *t*_start_ at the expense of ON+ and OTHER populations, and reaches its maximum at *t*_end_. With ligand, dynamics are fundamentally different: OFF+ and ON+ populations expand simultaneously from *t*_start_ until they surpass OTHER, then competing and driving the system into steady state at *t*_end_. The third subphase displays ligand-dependent outcomes: dominance of OFF cluster (without ligand) vs. ON:OFF co-existence at 40%:60% (with ligand). Taken together, these results highlight steady progress toward structural homogeneity and system stabilization. Revisiting the SHAPE-Seq profiles in Fig. 3e, f, one can see how these results manifest in the data. With ligand, the mixed ON:OFF population is consistent with intermediate reactivities at the 3′ end (f) as compared to suppressed ones without ligand (e). The mixture is further supported by the reduction in P1 loop’s reactivity associated with ligand presence. SLEQ analyses of 10 independent statistical samples at 5 intermediate transcript lengths with and without fluoride resulted in similar findings (Supplementary Methods).

Despite differing steady-state distributions between the two conditions, the OTHER population steadily declines from 80% to extinction. This led us to consider how structural diversity evolves over time, which we evaluated by average Shannon entropy of base-pairing (Methods). To estimate pairing probabilities for each nucleotide, we used SLEQ’s predictions, which simplify ensemble dynamics into a relatively small number of feasible states and therefore underestimate the entropy of the true ensemble. Low/high entropy indicates low/high diversity, respectively. Entropies shown in Supplementary Fig. 7 highlight the phase dependence of structural diversity. Entropies are fairly stable until terminator formation (subphase 1), then decline (subphase 2) until after terminator fully forms, and stability is reestablished (subphase 3). Generally, fluoride’s presence results in lowered entropies, suggesting that ligand-induced interactions constrain the system, which aligns with our understanding of ligand-binding thermodynamics^3^. Interestingly, relationships reverse near *t*_end_, possibly due to convergence to one vs. two states.

Further insights into *crcB* fluoride riboswitches were previously gained from functional mutagenesis studies^35^. We thus analyzed mutants cotranscriptional data to test how well structural dynamics correlate with function measurements (see Supplementary Fig. 8a and Supplementary Fig. 8 in ref. ^35^). Mutations were introduced to disrupt base pairs in PK1 stem only (M18), in both PK1 and CT (M19), or in CT only (M20). Mutations were also combined: M21 = M18 + M19 restores PK1 but affects CT, M22 = M19 + M20 restores CT but affects PK1, and M23 = M18 + M19 + M20 restores PK1 and CT (Supplementary Table 3). SLEQ’s outputs confirmed disruption and restoration of PK1 and CT in all mutants, as PPPs show (Supplementary Fig. 8b–g). Further, they highlighted that targeting PK1 bears more dramatic effects compared to CT. Specifically, PK1 does not form in M18 and M19, whereas CT is visible in M19 and M20. Nevertheless, such limited disruption suffices to render CT nonfunctional^35^.

Functional fluoride-induced outcomes of these mutations were assessed at the protein level via reporter fusion assays^35^ and at the RNA level via transcript readthrough quantification^34^. To link structure to function, we considered all structures that are functional, i.e., that may explain measured expression levels. Similar to our analysis of wild-type (wt) sequence, we manually clustered SLEQ’s outputs by their inclusion of key functional state-specific motifs. As before, if a structure has PK1, it is assigned to the ON+ class. However, in contrast to wt, we are also interested in structures that contain P1 and CT with disrupted base pairs, which are assigned to a class called DEF-OFF+. This is because mutants with such defective CT are unable to shut down transcription and thus contribute to protein levels. We then quantified the relationship between these clusters and protein levels (see Supplementary Fig. 9B in ref. ^35^). Supplementary Fig. 9a depicts ON+ and DEF-OFF+ abundances along with protein levels. It is apparent that neither ON+ nor DEF-OFF+ solely explain discrepancies in protein levels among mutants, supporting their joint contribution to gene expression. To uncover their relative contributions, we fitted a linear model

1 $${\mathrm{Protein}}\,{\mathrm{level}} = \alpha \,{\mathrm{Abundance}}\,{\mathrm{(ON + )}} \\ + \beta \,{\mathrm{Abundance}}\,\left( {{\mathrm{DEF}} \text{-} {\mathrm{OFF + }}} \right)$$

and normalized *α*, *β* to obtain relative contributions 0.66 and 0.34, respectively (Supplementary Fig. 9b). These results allude to superior translation efficiency of ON structures and to translation-inhibiting activity via ribosome binding-site sequestration^38^. As final note, we point out that the two-state composition shifted in favor of OFF state when attempting to recover WT functionality with M23. It may be because replacing a G-C pair with A-U exerts stronger destabilizing effects on PK1 compared to CT. This recapitulates our previous finding that PK1 is easier to disrupt (Supplementary Fig. 8). See Supplementary Methods for further details and analysis of additional data sets.

### Performance evaluations and comparisons

We compared SLEQ to RING-MaP^23^ and M^2^-REEFFIT^22^, which also reconstruct information on multiple structures from SP data. RING-MaP extracts distinct reactivity profiles from mutation data by clustering reads according to their modification patterns. It mines correlations between multiple modifications that reside in the same read, hence it only utilizes a subset of the reads and necessitates very high modification rates. For each identified cluster, RING-MaP outputs its respective reactivity profile and population fraction. As RING-MaP only outputs profiles, it can be supplemented by other methods to obtain data-directed single secondary structure predictions for each profile^39–41^. We applied RING-MaP to the *MRPS21* DMS-MaPseq data and compared reconstructions to its ground truth. Overall, performances were comparable, with both SLEQ and RING-MaP deviating by a few percents from ground truth in estimating allele population fractions. To compare predicted structures, we fed RING-MaP’s output profiles to RNAstructure^39^—predicted structures were close to both the references and SLEQ’s predictions. The complete details are found in Supplementary Methods. It is worth noting, however, that by summarizing DMS-MaPseq reads as truncation patterns in silico, we have demonstrated that in this data set, reads with a single modification, or equivalently, the reactivity profile (Fig. 2e), contain sufficient information for accurate reconstruction. While SLEQ can extract this information from both data types, RING-MaP necessitates generation of mutation data.

M^2^-REEFFIT reconstructs RNA landscapes from mutate-and-map (M^2^) data sets consisting of reactivity profiles of the wt and all single mutant sequences. Similarly to SLEQ, it applies “sample and select” principles, yet it relies on the premise that the wt and mutants ensembles share a common set of alternative structures, whose populations are re-distributed upon sequence mutations. Furthermore, Cordero and Das^22^ reported that they were unable to properly recover landscapes from wt profiles alone and that mutants data were critical to reconstruction.

We compared SLEQ and M^2^-REEFFIT reconstructions on three RNAs analyzed from real data in ref. ^22^ and for which ground truth information is available from NMR or crystallography studies: a bistable RNA (BST), *Vibrio vulnificus* adenosine deaminase (*add*) riboswitch and *Escherichia coli* 16S ribosomal RNA (rRNA). To bridge between the electrophoresis-based measurements analyzed in ref. ^22^ and sequencing readouts, we applied a simulation technique we previously developed^42^. Complete details of all comparisons and our simulation methodology are provided in Supplementary Methods. Here, we briefly summarize our findings. For BST—an engineered RNA—comparison is straightforward as only two alternative hairpins co-exist. Both methods generated accurate reconstructions, although SLEQ did so with much greater precision between repeated runs. The landscape of the *add* riboswitch is far more complex, as NMR studies identified three underlying structures, each comprising of several helices (see Fig. 1a in ref. ^43^). In the absence of ligand, two structures (apoA and apoB) co-exist, whereas in its presence, another structure (holo) dominates. Here, we followed an approach similar to our fluoride riboswitch analysis, where we clustered SLEQ’s outputs by functional motifs. SLEQ’s reconstruction was consistent with NMR measurements with and without ligand. In the presence of ligand, M^2^-REEFFIT found that holo structure dominates but did not quantify its abundance. In the absence of ligand, M^2^-REEFFIT recovered population fractions of helices but not of combinations thereof, which renders a direct comparison to the complete structures output by SLEQ infeasible. We therefore converted our results to helix abundances and found good agreement for half these helices. Finally, we analyzed a 110-nt region of *E. coli* 16S rRNA where a crystallography-based model was in disagreement with a SHAPE-directed model^44^ (see Fig. 2h in ref. ^22^). M^2^-REEFFIT recovered a single dominant structure that is similar to the crystal structure but found no evidence for the SHAPE-directed structure. In contrast, SLEQ’s reconstruction supports co-existence of both crystal and SHAPE-directed structures. As Cordero and Das^22^ reported population fractions of helices, we converted SLEQ’s results to single-helix level and found that both methods generated highly consistent estimates for over half of the nine helices while deviating for remaining ones. While our results generally agree with the results by Cordero and Das^22^, SLEQ has consistently generated substantially improved precision between runs.

In this work, we utilized samples of suboptimal structures to create structural diversity. In such cases, structural variants of a target are often observed. To test SLEQ’s robustness to divergence of sampled structures from true structures, we performed a case study on cotranscriptional SHAPE-Seq data. We perturbed the ON and OFF structures by incrementally removing up to seven base pairs from key functional motifs. This resulted in 24 candidate sets that are missing ON or OFF but include their variants at varying degrees of divergence. SLEQ consistently selected variants as long as no >40% of the base pairs in a key motif were disrupted. However, variation in structures manifested in gradual re-distribution of population fractions (see Supplementary Methods for complete details). This case study highlights the importance of statistical samples’ quality, which may be improved via deeper sampling. In this work, we found that sample size of 1000 sufficed to observe complete hairpin motifs in repeated samples.

We further confirmed SLEQ’s accuracy and robustness by simulating different ground-truth landscapes and varying noise levels for numerous real and engineered RNAs. We also analyzed real data for two additional riboswitches and for fluoride riboswitch in equilibrium conditions. See Methods for details.

### Assumptions and limitations

A simplifying assumption in our model is that for a given sequence, all unconstrained nucleotides in any structure are statistically identical in their propensity to be modified by SHAPE. For DMS, all unconstrained adenines display identical modification propensity and similarly for all unconstrained cytosines. We further assumed that constrained sites cannot be modified and that modification and noise dynamics are statistically independent between sites. Differences in reactivities are thus entirely attributed to ensemble dynamics. While discrepancies in modification propensities are known to exist^45,46^, these simplifications serve to reduce the model’s dimensionality in the interest of retaining its simplicity and alleviating risks of overfitting.

To ensure robust parameter estimation and reliable modeling, high-quality data are necessary, as evident from our simulations (see Methods, Supplementary Methods, and Supplementary Note 1). It is therefore necessary, albeit sometimes insufficient, to deeply sequence a region of interest, as sequencing coverage is a major determinant of data quality^42^. For transcripts of low abundance, this can be achieved by targeted priming^32^. Our simulations indicate that average coverage per nucleotide of 10^3^ is sufficient for reproducible reconstructions. Nevertheless, we recommend assessing reproducibility from biological replicates when available and also from in silico bootstrap replicates, as the latter capture technical variation at a given sequencing depth (see ref. ^47^ for SP-specialized bootstrap techniques). We find that such approach is more informative on coverage requirements than general guidelines derived from select examples because in our experience, performance also depends on other properties, such as information content of the data with respect to regions of differences between alternative structures. Other determinants to data quality include noisy cellular environment and biases in read counts introduced by library preparation steps such as PCR amplification and random priming^48^. To minimize such issues, we considered data obtained by targeted priming in well-optimized in vitro conditions. Additionally, SLEQ’s model directly accounts for 5′ bias incurred in targeted-priming truncation protocols^42^ (see Methods for details).

By design, “sample and select” approaches are strongly dependent on the initial sample of candidate structures. Its completeness is thus critical to accurate reconstruction in the sense that the correct structures must be included a priori. In this work, we generated candidates using statistical sampling alone or combined with other sources of prior knowledge (e.g., PK1 spike-ins). While generating suboptimal structures is generally useful as a way of enriching structural diversity among candidates^49^, current approaches rely on thermodynamic models. In some situations, e.g., when considering long RNAs or non-nested motifs, it is difficult and sometimes impossible to ensure that the correct key structures are generated, even when one samples deeper. There are several reasons for this limitation, including simplifications made in the thermodynamic model, exclusion of certain motifs from consideration to reduce complexity, insufficient sample size, and numerical accuracy limitations. Such uncertainty poses the greatest challenge to working with SLEQ, as SLEQ neither supports de novo structure generation nor it alerts the user that a key structure is missing. To help discern such situations, we recommend to visually compare the reactivity profile against an “ideal” profile that is expected from SLEQ’s reconstruction. The latter is indicative of local trends we expect to see in the data, i.e., where we expect reactivities to peak or decline according to motifs that dominate the reconstructed ensemble. Due to the stochastic nature of SP data and simplifying assumptions made in SLEQ’s model, one should not expect near-perfect matches between model-based and real reactivities. However, one should seek good agreement when it comes to clear trends observed in the data. Specifically, local peaks of real reactivities should be well-captured by the ideal model-based pattern, such that the model’s inability to reproduce such peaks should serve to alert a user that unconstrained motifs might be missing. Details of the proposed routine are found in Supplementary Methods along with examples of reconstructions when key structures are missing.

It is also worth noting that methods for structure generation other than those used here may be helpful in approaching completeness of candidate sets. For example, suboptimal pseudoknotted structures can be generated by specialized algorithms^39,50,^ and SP data can be used to direct ensemble sampling the same way it has been used to improve single-structure prediction^44,51^. Alternatively, one could enhance diversity by sampling the wt and all SNP sequences^22,52^. This approach, inspired by riboSNitches, aims at revealing structures that are rare in wt but emerge upon SNP introduction. Another direction, recently taken by RING-MaP, mines several constituent reactivity profiles directly from SP reads. These may subsequently be used to direct candidate generation.

To simplify modeling of complex structural dynamics, SLEQ seeks a parsimonious solution. In linear modeling, the most common fitting routine is the method of least squares. To guarantee non-negativity of abundances, non-negative least squares (NNLS) is warranted. Nonetheless, NNLS is not guaranteed to yield sparse solutions. In this work, NNLS consistently yielded sparse solutions in extensive analyses of simulated and real data sets with multiple candidate samples and for numerous small RNAs (see Supplementary Methods and Supplementary Note 1). In these cases, non-negativity constraints sufficed to constrain the solution space, allowing us to opt for a simple and unbiased method such as NNLS. Otherwise, least absolute shrinkage and selection operator (LASSO) is commonly used to impose sparsity^53^. Due to potential estimation bias associated with LASSO and the challenge in appropriately choosing its regularization parameter, we recommend using the simpler NNLS whenever it yields a sparse solution. Otherwise, a two-step approach may be more appropriate, where LASSO is applied to limit the number of selected structures, followed by NNLS for accurate abundance estimation^54^. Also, one may cluster either the input structures or the structures selected by NNLS using methods specialized to RNA structure^4,24,26^. However, clustering might eliminate features that may be informative to downstream analysis. Finally, we stress that SP data contain only partial information on structures. First, structures are assayed as sequences of constrained/unconstrained nucleotides as opposed to base-pair patterns. This inherently limits SP’s ability to distinguish between structures that have distinct base pairs but similar pairing states. Second, reactivities display large variation within a given structural context (i.e., paired/unpaired), manifesting in occasional discrepancies between reactivity magnitudes and the true structural context^17,20^. Such situations of imperfect information might lead to mispredictions.

## Discussion

We developed SLEQ to aid experimentalists in quantitatively gauging RNA structure landscapes. Ultimately, the goal is to elucidate sequence–structure–function relationships that govern RNA-based regulation in natural and engineered systems^9,55,56^. Of particular interest are situations where dynamics are significantly impacted by multiple structural states, as current computational approaches fall short in their ability to model them. Here, we explored several such scenarios and demonstrated SLEQ’s capability to glean quantitative information on system dynamics.

In recent years, SP experiments have expanded in both scope and depth to encompass diverse modification and detection strategies, library preparation protocols, sequencing choices, and analysis pipelines. Yet, these methods also share a common workflow^19^, which we leveraged in SLEQ’s design. Besides accommodating popular reagents such as SHAPE and DMS, SLEQ unifies the treatment of two major approaches to modification detection: truncation and mutation. This is particularly relevant to landscape studies because of fundamental differences in single-molecule information they extract. Specifically, only mutational profiling assays joint pairing states of nucleotides in individual structures, thereby accessing more complete information on ensemble composition. Although we observed subtle differences between mode-specific reconstructions from DMS-MaPseq data, we attribute this to the negligible frequency of reads with multiple modifications that are feasible over a stretch of only 11 informative nucleotides. Nonetheless, differences will presumably arise in scenarios where truncation data might possess limited discriminatory power with respect to a candidate set and when the region of interest is sufficiently long to feature substantial fraction of reads with single-molecule information. The richer set of mutation patterns would then impose additional constraints that can potentially drive SLEQ to parsimonious solutions. Interestingly, in simulations, we found mutation data to better-direct SLEQ toward the ground truth in the presence of high noise levels, exemplifying one potential advantage (Supplementary Note 1). This is particularly important in light of the recent explosion in transcriptome-wide in vivo data sets, which are generally noisy due to dramatic variations in transcript coverages.

Besides accommodating diverse data sets, SLEQ is versatile with respect to structure modeling, as a breadth of structural motifs are admissible, encompassing tertiary, non-canonical, inter-molecular, and non-nested interactions. This is in contrast to popular thermodynamics-based methods, which limit consideration to nested canonical secondary structures^57^. Two SLEQ features allow circumventing such limitation. First, structure prediction is decoupled from thermodynamic modeling: structures are specified a priori, and subsequently only data guides their selection. Moreover, candidate specification may or may not rely on thermodynamic modeling. Second, our model captures susceptibility to modification, whose determinants extend well beyond canonical base-pairing. For example, G-quadruplex motifs can be “encoded” into nucleotide modification susceptibilities^58^, and riboswitch-ligand interactions that alter nucleotide accessibility to modification can be captured by constraining them. Nevertheless, we stress that SLEQ does not accommodate three-dimensional structures, as such high-resolution models must be translated into binary representation of nucleotide states as constrained/unconstrained based on our knowledge of their susceptibility to modification.

Importantly, the decoupled design that confers SLEQ with much flexibility also brings about a major weakness, namely, its reliance on completeness of candidate sets. As SLEQ’s prediction via selection is constrained by prior knowledge, a landscape cannot be faithfully portrayed in the absence of the correct landmarks. Other confounding factors are data quality and information content. As mentioned earlier, SLEQ is a data-directed algorithm and its predictions are susceptible to noise. Further, known discrepancies between observed modifications and assumed structural contexts contribute to mispredictions^20^. Structure profiling also cannot reveal base-pairing partners and therefore has limited capability to distinguish between distinct structures that share similar modification patterns. Broadly speaking, performance depends on how informative modifications are at sites where alternative structures differ.

Our work was motivated by recent advances and massive expansion in SP techniques, which have become widely accessible and are capable of delivering high-fidelity and high-resolution snapshots of structural states, aggregated over ensembles of co-existing structures. When these data sets are judiciously combined with statistical learning techniques, it becomes feasible to parse these bulk measurements into core constituent elements. The data-informed computational workflow we developed gives rise to a concise, albeit simplified, representation of complex biological processes—a building block that is integral to complex systems analysis. Such crisp outputs adjoined by integration of diverse data inputs render SLEQ a powerful and accessible framework that will accelerate discovery and design of new functional RNAs.

## Methods

### Binning reads into patterns

Read binning depends on the type of SP technique, as it defines the modification patterns that can be observed. Detection via mutation yields a superset of patterns, as multiple nearby modifications can be assayed within a single read (Fig. 1a, Supplementary Fig. 1), whereas truncation reveals only the modification that is closest to the priming or fragmentation site^31^. Obviously, one can readily reduce the dimensionality of the richer set of mutation-generated patterns by recording the first modification in each read (Supplementary Fig. 1). This essentially projects the data onto the set of truncation-generated singleton patterns, but notably, this reduction is irreversible. To leverage on the richness of information in mutation detection, SLEQ features two modes: mutation and truncation. Truncation mode is simpler and considers *P* = *L* + 1 patterns, where *L* is the RNA length^59^. In mutation mode, 2^*L*^ patterns are theoretically observable, but in practice, this number is substantially lower due to limitations on achievable modification rates^32^. It should be noted that when base-selective chemistries such as DMS are used, pattern numbers are smaller since only sites with detectable modifications are considered. Counts of reads-per-pattern are converted to frequencies, which form the observation (or response) vector **y** of the linear model.

### Candidate structures

We initialize SLEQ with a set of candidate structures. There is full flexibility in assembling these candidates, and here we describe several straightforward ways to accomplish this task. For example, popular softwares for secondary structure analysis can be used to generate a pre-specified number of structures that are either nearly optimal or represent a statistical sample of the Boltzmann ensemble^50,60–62^. In this work, we used ViennaRNA^50^ to generate independent samples of 1000 structures, as this sample size is generally considered sufficient for statistical reproducibility in first-order sample statistics such as base-pair frequencies^24^. We further verified that such sample size is sufficient by applying SLEQ to 10 statistical samples and testing the reproducibility of its reconstructions (Supplementary Methods). Occasionally, and especially for small RNAs, sampling may be sufficiently deep with respect to the structure landscape, such that duplicates emerge and need to be removed.

Alternatively, one can manually determine which structures to consider as candidates, which may be useful when prior knowledge of the biological system is available. It is also possible to combine approaches by spiking in structures of interest into software-generated samples, as we demonstrated in our riboswitch folding pathway analysis. In that case, standard statistical sampling algorithms preclude pseudoknoted structures from consideration^24^, hence “spike-in” served to alleviate this constraint. Alternatively, one may use samplers specialized to account for pseudoknots^39,50^. It is also worth noting that structures need not conform to common limitations in RNA structure analysis. Specifically, many popular methods restrict analysis to nested secondary structures due to computational complexity limitations, whereas SLEQ handles pseudoknots, non-canonical motifs, and tertiary interactions in a fashion similar to standard base pairs. SLEQ’s generalized treatment is facilitated by its explicit modeling of each nucleotide’s susceptibility to modification (see subsection Design matrix), as it may be influenced by factors other than canonical base-pairing interactions. For example, the pseudoknot and LR interactions characteristic of the *crcB* fluoride riboswitch were modeled by representing all constrained nucleotides as paired (Supplementary Methods).

### Linear model

Fundamental to our approach is a mathematical description of the aggregate degrees of modification observed at each nucleotide as a linear superposition of modifications at individual molecules. From a statistical perspective, all molecular copies that fold into a given structure are identical in terms of their propensity to generate each modification pattern. Hence, the unknown relative abundances of each structure are the parameters of interest. Structure abundances are then combined with likelihoods of pattern generation by each structure, such that the observed frequency of each pattern is expressed as linear superposition of the probabilities of its generation by each structural population. This can be written as :

2 $${\bf{y}} = {\mathrm X} {\bf{\rho}} + {\bf{\varepsilon }},$$

where **y** ∈ ${\Bbb R}^P$ is a vector of *P* observations, **X** ∈ ${\Bbb R}^{P \times S}$ is a design matrix that captures the relationship between observations and relative abundances of candidate structures, **ρ** ∈ ${\Bbb R}^S$ is a vector of unknown relative abundances of *S* candidate structures, and **ε** ∈ ${\Bbb R}^P$ is a vector of *P* error terms. Observation *y*_*p*_ is the frequency of pattern *p* in the data obtained from the treated sample. A design matrix entry *x*_*ps*_ = Pr(pattern *p* | structure *s*) stands for the conditional probability of observing a read of pattern *p* given that it originates from structure *s*. See subsections Binning reads into patterns and Design matrix for details of their calculation. Our goal is to find the relative abundances of candidate structures (**ρ**) that best explain the observations and to output all structures with non-zero abundance.

### Probabilistic model

To construct **X**, we introduce additional parameters, whose estimates are mode-dependent, and a probabilistic model, which we use to populate **X**’s entries. For simplicity, we limit attention to data obtained with chemistries that are indifferent to a nucleotide’s base identity, such as SHAPE. Minor adjustments to this framework are warranted for data from base-selective probes, such as DMS, as described in Supplementary Methods.

To describe SLEQ’s model, we first briefly review a previous model on which it extends^28,59^. It was developed to estimate reactivity profiles in truncation experiments to correct for biases introduced by coverage discrepancies arising from random priming or from directionality in cDNA synthesis^29,42,59^. To simplify the exposition, we limit discussion to truncation protocols, however, a similar framework applies to mutation data, as will become evident from SLEQ’s model derivation.

For an RNA of length *L*, we number its nucleotides 1 to *L* from 3′ to 5′, where the priming site is adjacent to the 3′-end. Hereafter, we refer to nucleotides as sites. We define the reactivity of site *l*, *β*_*l*_, as the probability of modification at this site. Since observed read counts reflect a combined effect of modification and natural (i.e., not modification-based) transcription termination, the latter is treated as noise and measured in a mock-treated control sample that undergoes the same protocol. Because natural propensity to terminate transcription may vary between sites, for each site *l*, we define *γ*_*l*_ as the conditional probability of termination at said site due to noise. We now have two *L*-dimensional parameter vectors, **γ** and **β**, capturing degrees of noise and modification, respectively. We use them to express the probability of each cDNA fragment that may be observed in the experiment or control. In the control, the probability of elongating up to and including site *l* − 1 and terminating at *l* is

3 $$\mathrm{Pr}\left( {{\mathrm{read}}\,{\mathrm{span}}\,{\mathrm{sites}}\,{\mathrm{1}}\,{\mathrm{to}}\,l - 1\,{\mathrm{in}}\,{\mathrm{control}}} \right) = \gamma _l\mathop {\prod}\limits_{k = 1}^{l - 1} \left( {1 - \gamma _k} \right).$$

In the experiment, elongation continues up to *l* − 1 as long as there is no natural termination or modification at any site in between the primer and *l*. This happens with probability $\mathop {\prod}\nolimits_{k = 1}^{l - 1} \left( {1 - \gamma _k} \right)\left( {1 - \beta _k} \right)$. Termination at *l* happens either naturally or if *l* is modified, hence with probability 1 − (1 − *γ*_*l*_)(1 − *β*_*l*_). Taken together, the probability that a read of length *l* − 1 is observed is

4 $$\begin{array}{l}\mathrm{Pr}\left( {{\mathrm{read}}\,{\mathrm{span}}\,{\mathrm{sites}}\,{\mathrm{1}}\,{\mathrm{to}}\,l - 1\,{\mathrm{in}}\,{\mathrm{experiment}}} \right)\\ = \left( {1 - \left( {1 - \gamma _l} \right)\left( {1 - \beta _l} \right)} \right)\mathop {\prod}\limits_{k = 1}^{l - 1} \left( {1 - \gamma _k} \right)\left( {1 - \beta _k} \right).\end{array}$$

Also, one may observe complete reads of length *L* when no truncation occurs, in which case the probabilities are $\mathop {\prod}\nolimits_{k = 1}^L \left( {1 - \gamma _k} \right)$ and $\mathop {\prod}\nolimits_{k = 1}^L \left( {1 - \gamma _k} \right)\left( {1 - \beta _k} \right)$ in control and experiment, respectively.

It is worth noting that the model by Aviran et al. treats all RNA copies in a sample as if they adopt a single structure that displays the average modification properties of the underlying ensemble. Furthermore, it does not consider explicit structures or nucleotide pairing states as inputs nor does it link a structure to observed reads. It merely links the proportion of molecules modified at each nucleotide (**β**) to observed reads. In what follows, we populate SLEQ’s design matrix with expressions derived from an extended version of this model, which links a structure to read modification patterns.

In SLEQ’s model, we make a simplifying assumption by treating all structurally unconstrained nucleotides in any given structure (folded from the same sequence) as statistically identical in their propensity to be modified. We also assume that paired/constrained sites cannot be modified. We then define *η* as the probability of modification at unconstrained nucleotides. Similarly to the model by Aviran et al., *γ*_*l*_ is a noise term, capturing the probability of truncation (mutation) at *l* in truncation (mutation) experiments. However, the models differ in the way they treat modifications, as here, *η* is used in place of Aviran et al.’s (*β*_1_, … *β*_*L*_).

In truncation mode, we consider *P* = *L* + 1 modification patterns, where pattern *p* (1 ≤ *p* ≤ *L*) corresponds to truncation at site *p* and *p* = *L* + 1 represents a complete read that arises when no truncation occurs. To calculate entry *x*_*ps*_ in **X**, we consider the following events and respective probabilities:

1. ${\cal A}$: given that *l* is unpaired, truncation occurs at *l* due to modification or noise.
  5 $$\mathrm{Pr}({\cal A}) = 1 - (1 - \eta )(1 - \gamma _l).$$
2. ${\cal B}$: given that *l* is unpaired, reverse transcriptase (RT) reads through *l*.
  6 $$\mathrm{Pr}({\cal B}) = (1 - \eta )(1 - \gamma _l).$$
3. ${\cal C}$: given that *l* is paired, truncation occurs at *l* due to noise.
  7 $$\mathrm{Pr}({\cal C}) = \gamma _l.$$
4. ${\cal D}$: given that *l* is paired, RT reads through *l*.
  8 $$\mathrm{Pr}({\cal D}) = 1 - \gamma _l.$$

Given a pattern *p* and structure *s*, we denote the sets of unpaired and paired nucleotides within the first *p* − 1 sites by U_*ps*_ and P_*ps*_, respectively. Thus, *x*_*ps*_, the probability that structure *s* generates pattern *p*, is calculated as follows.

1. If site *p* is unpaired in *s*:
  9 $$\begin{array}{*{20}{l}} {x_{ps}} \hfill & = \hfill & {\mathop {\prod}\nolimits_{l \in {\mathrm{U}}_{ps}} {(1 - \eta )\left( {1 - \gamma _l} \right)} } \hfill \\ {} \hfill & {} \hfill & {\mathop {\prod}\nolimits_{l \in {\mathrm{P}}_{ps}} {(1 - \gamma _l)[1 - (1 - \eta )(1 - \gamma _p)]} } \hfill \end{array}.$$
2. If site *p* is paired in *s*:
  10 $$x_{ps} = \mathop {\prod}\nolimits_{l \in {\mathrm{U}}_{ps}} {(1 - \eta )(1 - \gamma _l)} \mathop {\prod}\nolimits_{l \in {\mathrm{P}}_{ps}} {(1 - \gamma _l)\gamma _p} .$$
3. For the complete read pattern *p* = *L* + 1, we obtain
  11 $$x_{L + 1,s} = \mathop {\prod}\nolimits_{l \in {\mathrm{U}}_{L + 1,s}} {(1 - \eta )(1 - \gamma _l)} \mathop {\prod}\nolimits_{l \in {\mathrm{P}}_{L + 1,s}} {(1 - \gamma _l)} .$$

In mutation mode, we consider the following events:

1. ${\cal A}$: given that *l* is unpaired, *l* is mutated due to modification or noise.
  12 $$\mathrm{Pr}({\cal A}) = 1 - (1 - \eta )(1 - \gamma _l).$$
2. ${\cal B}$: given that *l* is unpaired, no mutation occurs at *l*.
  13 $$\mathrm{Pr}({\cal B}) = (1 - \eta )(1 - \gamma _l).$$
3. ${\cal C}$: given that *l* is paired, *l* is mutated due to noise.
  14 $$\mathrm{Pr}({\cal C}) = \gamma _l.$$
4. ${\cal D}$: given that *l* is paired, no mutation occurs at *l*.
  15 $$\mathrm{Pr}({\cal D}) = 1 - \gamma _l.$$

Given a pattern *p* and structure *s*, we denote the subsets of unpaired sites, which are mutated or non-mutated as UM_*ps*_ or UN_*ps*_, respectively. Similarly, the two subsets of paired sites are denoted as PM_*ps*_ and PN_*ps*_. Thus, *x*_*ps*_, the probability that structure *s* generates pattern *p*, is calculated as follows.

16 $$\begin{array}{*{20}{l}} {x_{ps}} \hfill & = \hfill & {\mathop {\prod}\nolimits_{l \in {\mathrm{UM}}_{ps}} {[1 - (1 - \eta )(1 - \gamma _l)]} } \hfill \\ {} \hfill & {} \hfill & {\mathop {\prod}\nolimits_{l \in {\mathrm{UN}}_{ps}} {(1 - \eta )(1 - \gamma _l)} } \hfill \\ {} \hfill & {} \hfill & {\mathop {\prod}\nolimits_{l \in {\mathrm{PM}}_{ps}} {\gamma _l} \mathop {\prod}\nolimits_{l \in {\mathrm{PN}}_{ps}} {(1 - \gamma _l)} .} \hfill \end{array}$$

### Design matrix

The entries of the design matrix are expressed as functions of the unknown modification probability at unpaired sites (*η*) and noise parameters (**γ**). To estimate *η*, it is helpful to consider a related vector from the model by Aviran et al., namely, **β**. Recall that *β*_*l*_ is the probability that site *l* is modified when considering all molecules in the sample^59^, or in other words, it is a modification propensity that is aggregated over the many different structures in the ensemble. Importantly, while *η* is kept constant across all unpaired sites within an ensemble of structures, *β*_*l*_ is both site-specific and an ensemble-based average measure. To illustrate the relationship between *η* and **β**, consider three sites with the following properties: *i* is unpaired in all structures, *j* is paired in all structures, and *k* is paired in some and unpaired in others. The respective ensemble-average probabilities would then be *β*_*i*_ ≈ *η*, *β*_*j*_ ≈ 0, and 0 < *β*_*k*_ < *η*. Thus, estimating **β** at sites such as *i* allows one to infer *η* at reasonable accuracy. However, not knowing which candidate structures are highly abundant, it is not trivial to identify such sites. A conservative approach is to seek sites that are unpaired in all candidate structures, but it may be challenging in cases where structural diversity is high. Here, we estimate *η* from $\hat {\bf{\beta}}$ empirically by sorting the $\hat \beta _l$’s from high to low, calculating their mean, and setting $\hat \eta _{}^{}$ as the median of those exceeding the mean. This approach is motivated by the expectation that *β*_*l*_ would be large at sites that are primarily unpaired in the ensemble. At the same time, it considers a subset of reactive sites to confer robustness to outliers, which are commonly observed in SP data^19^. If no sites that are unpaired across the ensemble exist, we are likely to underestimate *η*, which in turn will degrade prediction accuracy.

To estimate **β** and **γ**, we use maximum-likelihood estimates derived by Aviran et al.^59^:

17 $$\begin{array}{*{20}{c}} {\hat \gamma _l = \frac{{Y_l}}{{C_{l - }}};} & {\hat \beta _l = \frac{{\frac{{X_l}}{{C_{l + }}} - \hat \gamma _l}}{{1 - \hat \gamma _l}},} \end{array}$$

where *X*_*l*_ and *Y*_*l*_ (*l* = 1, 2, … *L*) are counts of truncations/mutations at site* l* observed in treated and control samples, respectively, and *C*_*l*+_ and *C*_*l*−_ are local coverages in said samples. Local coverage at site *l* in a sample stands for the sequencing depth at *l*, which definition is straightforward in mutation mode. In truncation mode, local coverage is defined as the number of reads that either stop at or pass through *l*^63^.

### Structure selection and relative abundance estimation

Prior to feeding the linear model with candidate structures and sequencing reads, we screen for inconsistencies between these two distinct inputs and remove structures that are markedly inconsistent with the data. Such dimensionality reduction serves to alleviate potential model overfitting and improve performance. Notably, the linear system in truncation mode has a relatively small number of patterns compared to mutation mode, which number is often substantially smaller than the number of structures considered, resulting in an under-determined system and the risk of overfitting. Pre-filtering of inconsistent structures then serves to alleviate such concern. A structure is deemed inconsistent with the data if there is sufficient evidence of nucleotides that are paired in this structure but are highly reactive. This is because we expect highly reactive sites to be predominantly unpaired in the ensemble. We define all sites where $\hat \beta _l > \hat \eta$ as highly reactive. Strictly, if a structure has one of these sites paired, it should be removed. However, $\hat \eta$ and $\hat {\bf{\beta}}$ might not always be highly accurate, especially when data are very noisy. Additionally, highly reactive sites are the most susceptible to measurement errors and typically have the highest variance^64^. For these reasons, we only remove structures that were inconsistent with more than a single nucleotide, specifically, more than half the highly reactive sites. Relative abundances were estimated using standard NNLS fitting. Structures whose estimated abundances exceed a user-specified threshold are selected, and then abundances are normalized to sum to 1. In this work, we thresholded at 1%.

### Post-processing SLEQ’s outputs

Structures selected by SLEQ and their abundance estimates were used to estimate the probabilities of each possible base pair (*P*_*ij*_ for any two sites *i* and *j*) and the probability that a site is unpaired (*P*_*ii*_ for any site *i*) in the reconstructed ensemble. For each site *i*, *P*_*ij*_ (*j* ≠ *i*) and *P*_*ii*_ form a probability distribution, whose Shannon entropy is^65,66^

18 $$\begin{array}{*{20}{l}} {H_i = - \mathop {\sum}\limits_{j = 1}^L P_{ij}\mathrm{log}_2\left( {P_{ij}} \right).} \hfill \end{array}$$

To account for variable lengths of intermediate transcripts, site-specific entropies were averaged over all sites. Base-pairing entropy was used as an approximation of structural entropy^67,68^ because selected structures change as sequence length increases, which renders the underlying distributions and their entropies incomparable. Furthermore, structures sometimes share certain motifs but not others, which is well-captured by base-pairing entropy but overlooked by structural entropy. All curves in Fig. 4 and Supplementary Figs. 4 and 7 are smoothed with a mean filter of window size 3 to improve signal-to-noise ratio.

### Performance evaluations via simulations

To further test SLEQ’s performance and robustness, we conducted extensive simulations spanning multiple RNAs, parametric regimes, and noise scenarios. For each tested RNA, we set a ground truth that consists of a small set of structures and their abundances along with *η*, **γ** values. Structures were chosen based on prior studies of these RNAs, and abundances relied on literature when available^22,69,70^. From ground truths, we calculated probabilities of observing each possible pattern in truncation and mutation modes and repeatedly drew from these distributions a pre-set number of sequencing reads, as previously described in^42^. These simulated data sets were analyzed as described above and results were compared to ground truth. Tests include varying the ground truth abundances of structures, *η*, and noise levels. Noise was introduced in two ways: read counts were perturbed by additive Gaussian noise and 10 randomly sampled decoy structures were added to ground-truth structures to populate 10% of the ensemble. The former noise term mimics measurement noise whereas the latter mimics more realistic structural dynamics in solution. Data were simulated for the following RNAs: preQ_1_^70^, VcQrr_3_^69^, TBWN, and MST^22^. See Supplementary Note 1 for more details and simulation results.

### Data pre-processing

Raw DMS-MaPseq reads were quality-trimmed and stripped of sequencing adapters using cutadapt (parameters “-q 25” and “-a CACTCGGGCACCAAGGA,” respectively)^71^. Reads were aligned to both allelic sequences using bowtie2 ver. 2.2.5 (parameters “–local D20 -R3 -N2 -L15 -i S,1,0.5 –score-min G,20,8 –ma 2 –mp 6,2 –rdg 5,1 -rfq 5,1 -x Allele”)^72^. Probed sequences are TGCTGCCATCTCTTTTCTTCTCTATGCGAGGATTTGGACTGGCAGTG (A allele) and ATCTCTTTTCTTCTCTCTGCGAGGATTTGGACTGGCAGTGAGAATAAGAGACAA (C allele). Since we analyzed the 33-nt region shared by the two allele-specific structures shown in ref. ^32^ (also see nucleotide labeled with letters in Fig. 2a), we retained reads that fully overlap this region. A ground truth for this experiment was obtained directly from read alignments. We then binned reads into >600 patterns that are restricted to the 11 adenines and cytosines included in the 33-nt region. The numbers of mutations over the 11 nucleotides were distributed as 71.3% (0), 25% (1), 3% (2), 0.2% (3) and 0.5% (≥4), yielding average modification rate of ~0.33, which implies that a mutation is observed on average every 33 informative adenines/cytosines nucleotides. For structure generation, we merged independent samples of 1000 structures from both sequences and removed those that formed duplicates over the 33-nt region. Since data did not include a control sample, we could not estimate noise levels and thus set **γ** to **0**.

SHAPE-Seq profiles for *crcB* fluoride riboswitch intermediate and complete transcripts were obtained in the form of counts of truncated transcripts. For each transcript length, all cDNA products span sequences between a common priming site at the 3′ end and a variable truncation site. Such targeted priming allowed us to recover local coverages at each site (see Design matrix to estimate **β** and **γ**). Since the experiment involves RNA polymerase arrest, its 14-nt footprint at the 3′ end was removed from all count profiles and additionally excluded from sequences prior to generating candidate structures.

### Code availability

Software implementation of SLEQ along with code for SP data pre-processing, simulations, and entropy calculations are freely available at https://github.com/AviranLab/SLEQ under the BSD-2 license^73^.

### Data availability

Original data sets used in this study are available at the links listed in Supplementary Table 4. Other data are available from the corresponding author upon reasonable request.

## Electronic supplementary material

Supplementary Information
