# Publisher Correction: Statistical modeling of RNA structure profiling experiments enables parsimonious reconstruction of structure landscapes

**DOI:** 10.1038/s41467-018-03519-y

**Published:** 2018-03-13

**Authors:** Hua Li, Sharon Aviran

**Affiliations:** Department of Biomedical Engineering and Genome Center, University of California at Davis, Davis, CA 95616 USA

Correction to: *Nature Communications* 10.1038/s41467-018-02923-8, published online 09 February 2018

The originally published version of this Article contained an error in Figure 2, due to a typesetting error. Panels d and e were positioned such that the locations of the mutations in panel d did not align correctly with the corresponding nucleotides in the reactivity profile in panel e. This has now been corrected in both the PDF and HTML versions of the Article.

