# Supplementary material for: Statistical modeling of RNA structure profiling experiments enables parsimonious reconstruction of structure landscapes
Source: Nat Commun. 2018 Feb 9;9:606. doi: 10.1038/s41467-018-02923-8 (PMC5807309; doi:10.1038/s41467-018-02923-8)
Supplement: Supplementary file 1 — Supplementary Information [file 41467_2018_2923_MOESM1_ESM.pdf]

“Statistical modeling of RNA structure profiling experiments enables parsimonious reconstruction of structure landscapes”

Li *et al.*

Contents

1 SUPPLEMENTARY FIGURES **4**

2 SUPPLEMENTARY TABLES **38**

3 SUPPLEMENTARY METHODS **69**

3.1 Pattern reduction 69

3.2 Design matrix for base-selective profiling techniques 69

3.3 Analysis of human *MRPS21* riboSNitch from DMS-MaPseq data 71

3.4 Analysis of *crcB* fluoride riboswitch from SHAPE-Seq data 72

3.5 Analysis of *crcB* fluoride riboswitch mutants from SHAPE-Seq data 73

3.6 Analysis of *crcB* fluoride riboswitch in equilibrium from SHAPE-Seq data 74

3.7 Analysis of *thiM* TPP and *add* adenine riboswitch aptamer domains from SHAPE-Seq data 75

3.8 Comparison with RING-MaP 75

3.9 Comparison with M^2-REEFFIT 76

3.9.1 Methodology 77

3.9.2 BST 77

3.9.3 *add* 78

3.9.4 16S 79

3.10 Performance of SLEQ on the perturbation of important structures 80

3.11 Performance of SLEQ in the case of missing important structures 82

3.11.1 BST 83

3.11.2 Human *MRPS21* riboSNitch 84

3.11.3 *crcB* fluoride riboswitch 84

3.12 Performance of SLEQ on different average sequencing depths 86

3.13 Performance of SLEQ on different Boltzmann ensembles 87

3.14 Comparison of mutation and truncation modes with real data 88

4 SUPPLEMENTARY NOTE 1: SIMULATIONS **88**

4.1 Performance as a function of η 88

4.2 Performance as a function of relative abundances 89
4.3 Robustness to noise in structure ensemble 89
4.4 Robustness to noise in SP data 90
4.5 Additional RNAs 90

1 SUPPLEMENTARY FIGURES

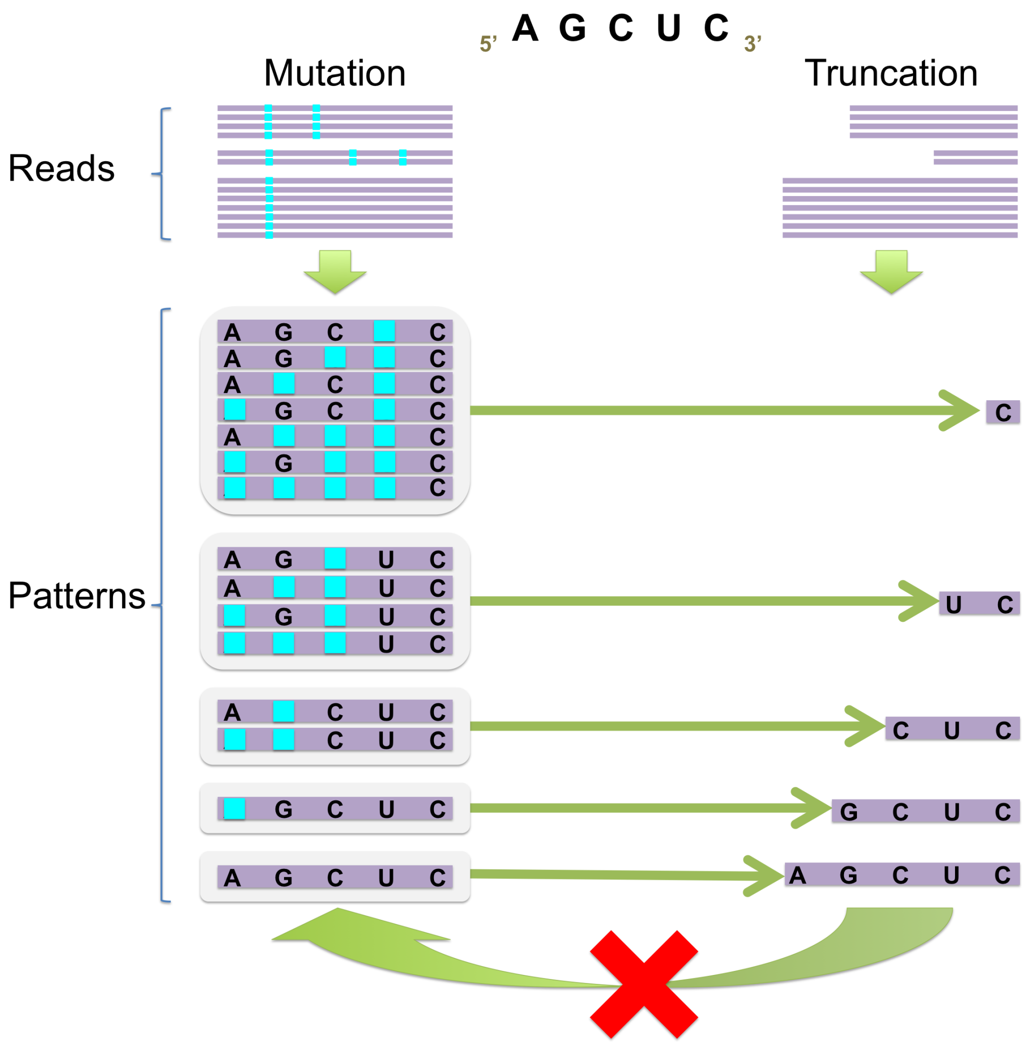

Supplementary Figure 1: **Binning reads into patterns in two SLEQ modes.** Read binning entails assignment of reads to patterns depending on the profiling technique. For example, the top 4 reads in mutation mode belong to one pattern, as they record mutations at identical sites. The top 4 reads in truncation mode should also be binned into a single pattern, as they both start and end at the same locations. Mutation profiling can detect multiple modifications per read (left column), resulting in more modification patterns compared to truncation profiling (right column). In contrast, detecting modifications via truncation preserves only the closest modification to the priming or fragmentation site, resulting in fewer patterns. One can readily reduce the richer set of mutation patterns into a compact set of truncation patterns by recording the first modification in each read (projection is depicted by arrows). For example, in the top projection, the first modification in all RNA copies is at the U site. If modifications are detected via truncation, cDNAs will stop at the U site. We therefore reduce all mutation pattern in the top list into a single truncation pattern. However, this projection is irreversible.

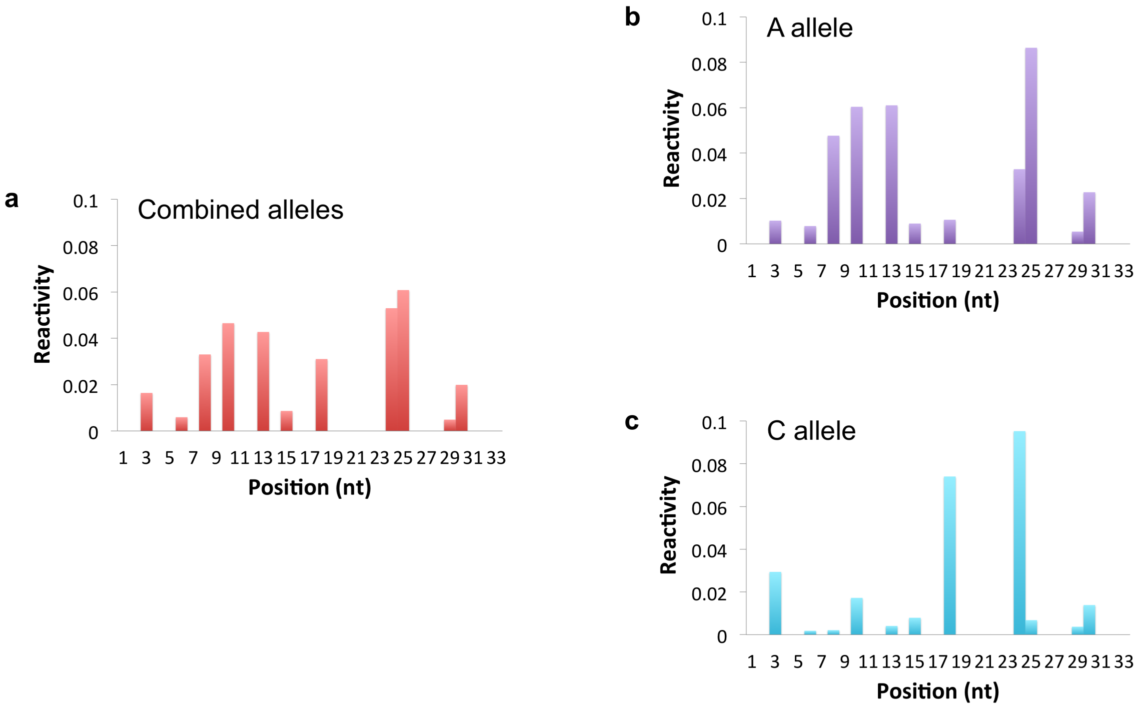

Supplementary Figure 2: **Reactivity profiles of combined (a) and separate alleles (b, c).** It is difficult to discern the two distinct structures from the combined reactivity profile.

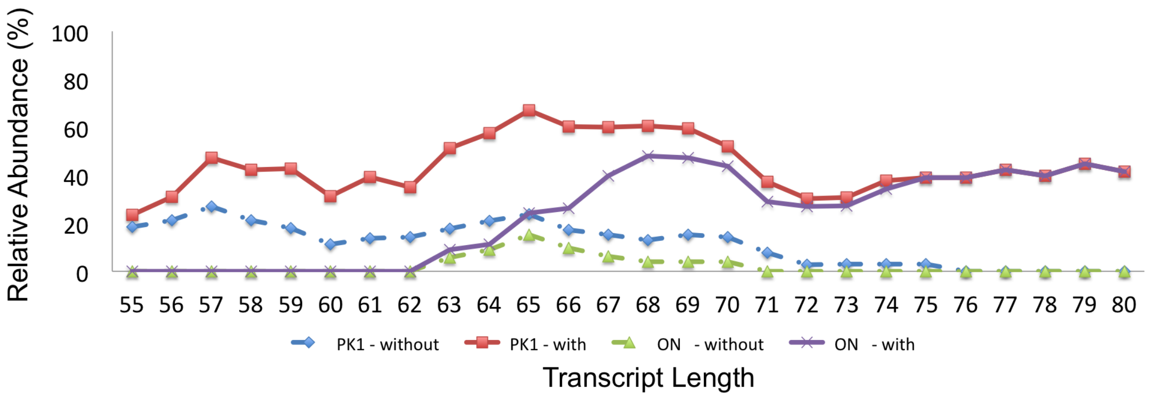

Supplementary Figure 3: **Abundances of ON structure and functional motif PK1 with and without ligand, depending on intermediate elongating transcript length for the *crcB* fluoride riboswitch.**

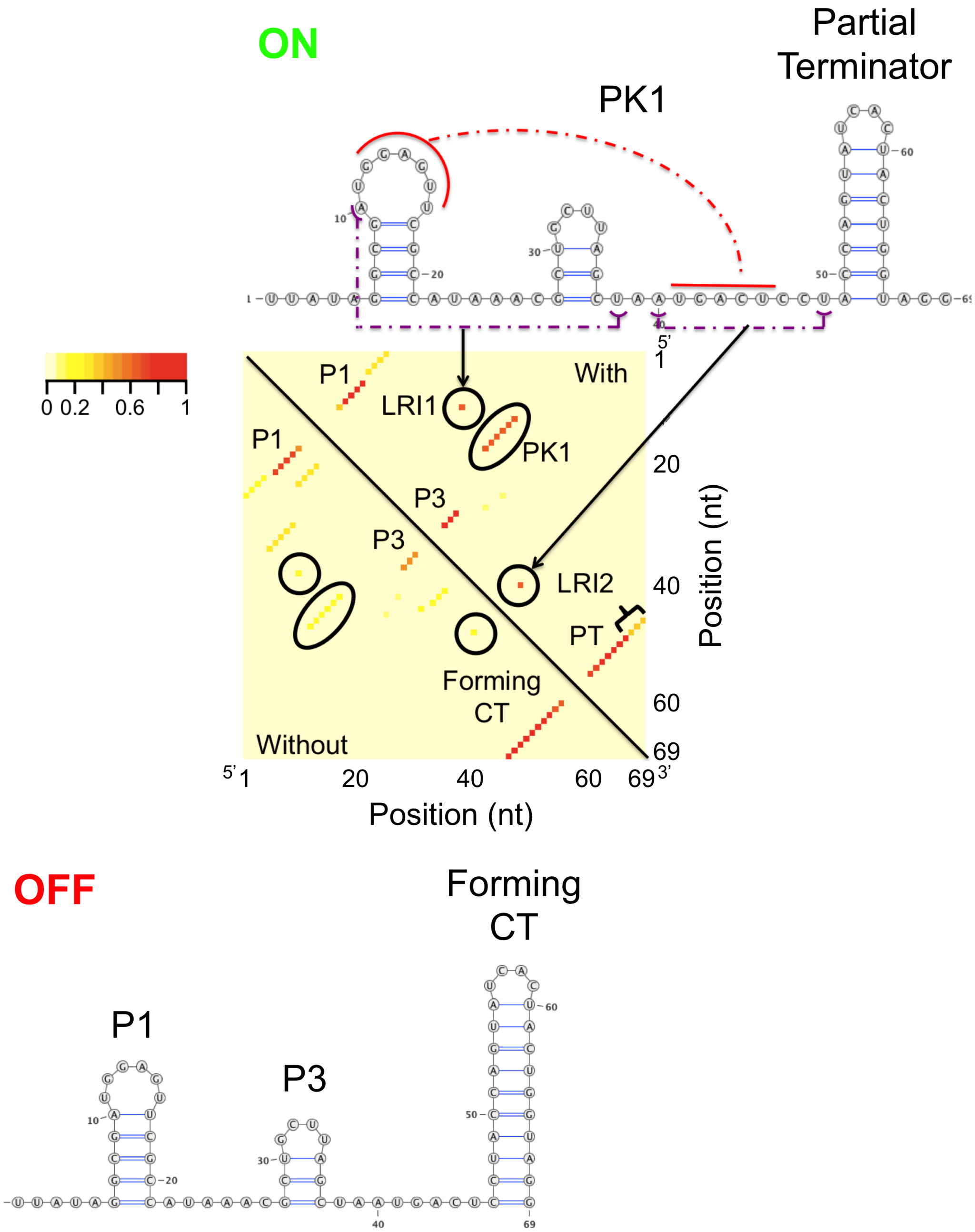

Supplementary Figure 4: **Visualization of SLEQ's outputs via pairing probabilities plots (PPP).** We applied SLEQ to analyze cotranscriptional SHAPE-Seq data of the *crcB* fluoride riboswitch at length 69 nt with/without ligand. From the PPP, it is evident that the OFF structure dominates without fluoride (see P1, P3, terminator helices below the diagonal line) while the ON structure is more abundant with ligand (see P1, P3, partial terminator, PK1 helices and LR above the diagonal line). The substantial elevation of PK1 and LR presence is highlighted by black ovals (note the transition from yellow, without ligand, to red, with ligand). Additionally, the prevention of CT formation in favor of aptamer interactions is evident from base-pair colors at the lower right corner (highlighted by braces). All structures in this paper were drawn using the VARNA package[1].

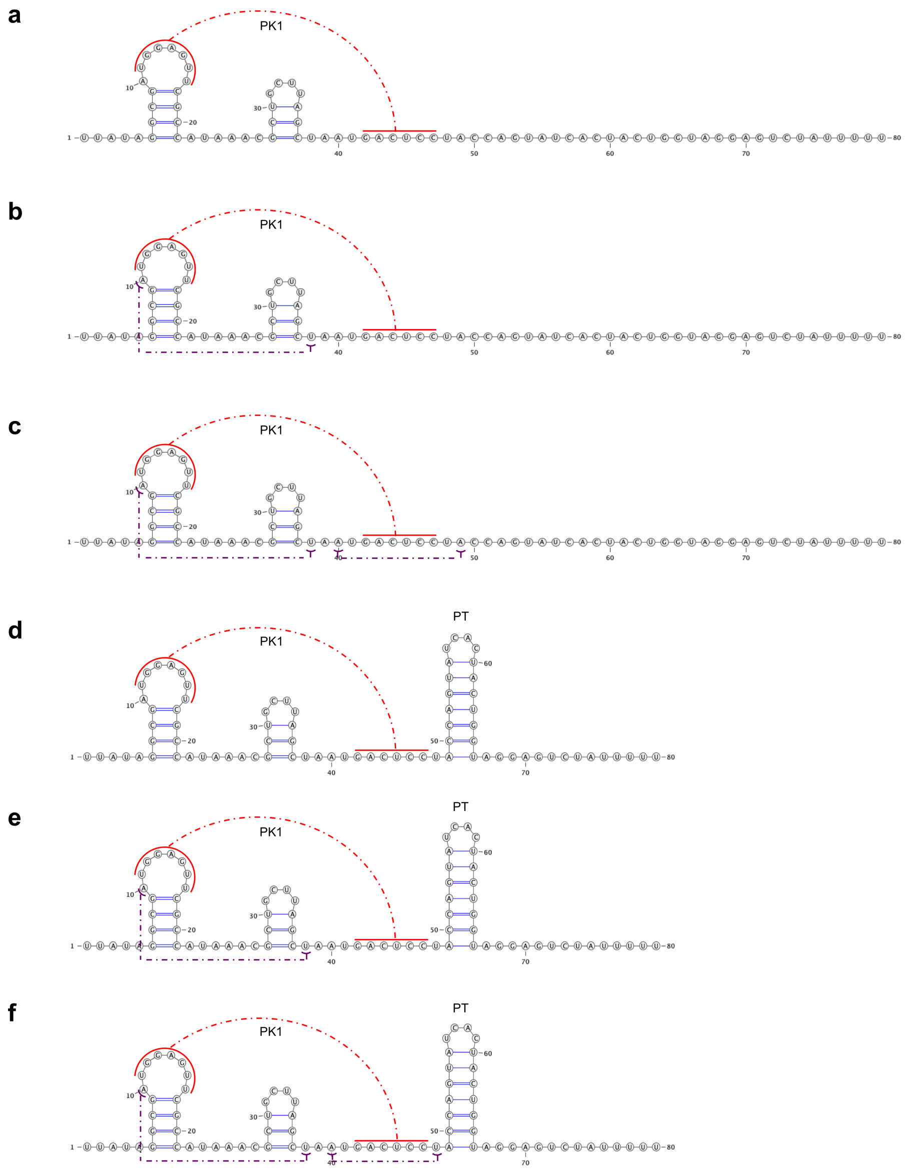

Supplementary Figure 5: **Pseudoknot-containing structures that were spiked into candidate sets.** As the transcript is elongated from 55 to 61 nt, we spike structures **(a), (b)** and **(c)** into candidate sets. As the transcript is elongated from 62 to 74 nt, we additionally spike in structures **(d), (e)** and **(f)**, because PT begins to form at 62 nt. **(a)** Structures only include PK1. As the transcript is elongated from 55 to 74 nt, we sequentially extend it with unpaired nucleotides. **(b)** Structures contain PK1 and LR1. The extension method is the same as in **(a)**. **(c)** Structures contain PK1 and two long-range interactions. The extension method is the same as in **(a)**. **(d)** Structures contain PK1 and partial terminator (PT).The extension method is as follows: as the transcript is elongated from 62 to 66 nt, we extend the structure by sequentially adding base pairs due to PT formation. After completeness of PT formation, from 67 to 74 nt, we extend the structure by adding unpaired nucleotides. **(e)** Structures contain PK1, LR1 and PT. The extension method is the same as in**(d)**.**(f)** Structures contain PK1, two long-range interactions, and PT. The extension method is the same as **(d)**.

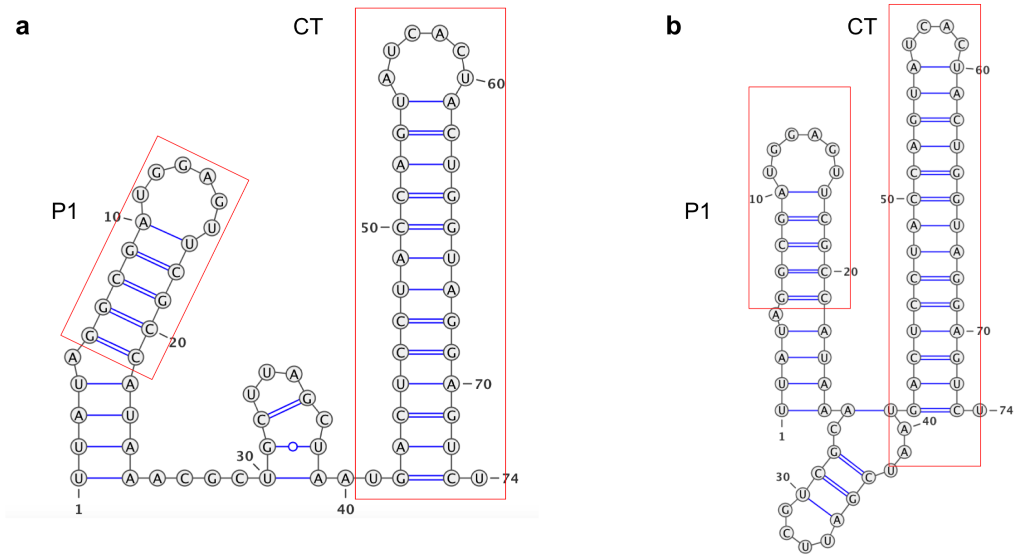

Supplementary Figure 6: **Examples of OFF+ structures for *crcB* fluoride riboswitch. (a)** One example of OFF+ structure. **(b)** The other example of OFF+ structure. Both structures have P1 and CT motifs, as required by our definition of the OFF+ cluster.

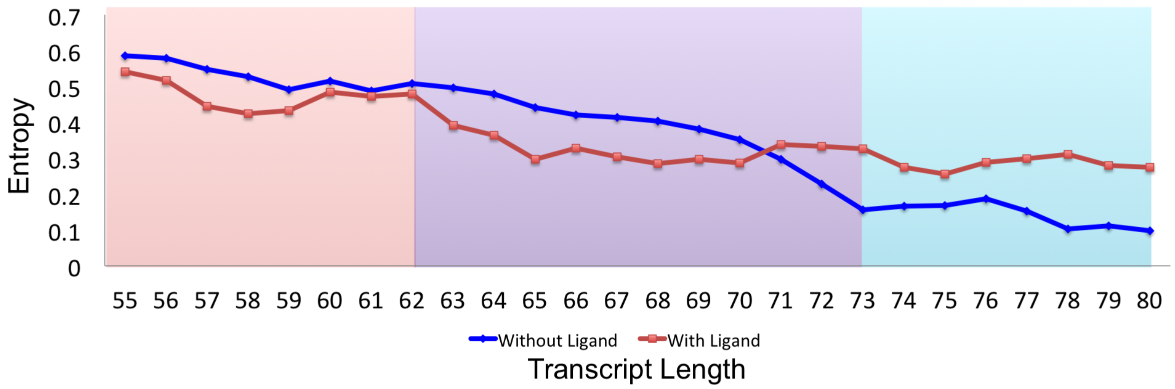

Supplementary Figure 7: **Quantitative analysis of entropies for the cotranscriptional structure landscape of the *crcB* fluoride riboswitch based on SLEQ outputs.** Base-paring entropies with and without ligand as a function of transcript lengths are depicted. Three sub-phases in fluoride-dependent system dynamics are highlighted by different background colors.

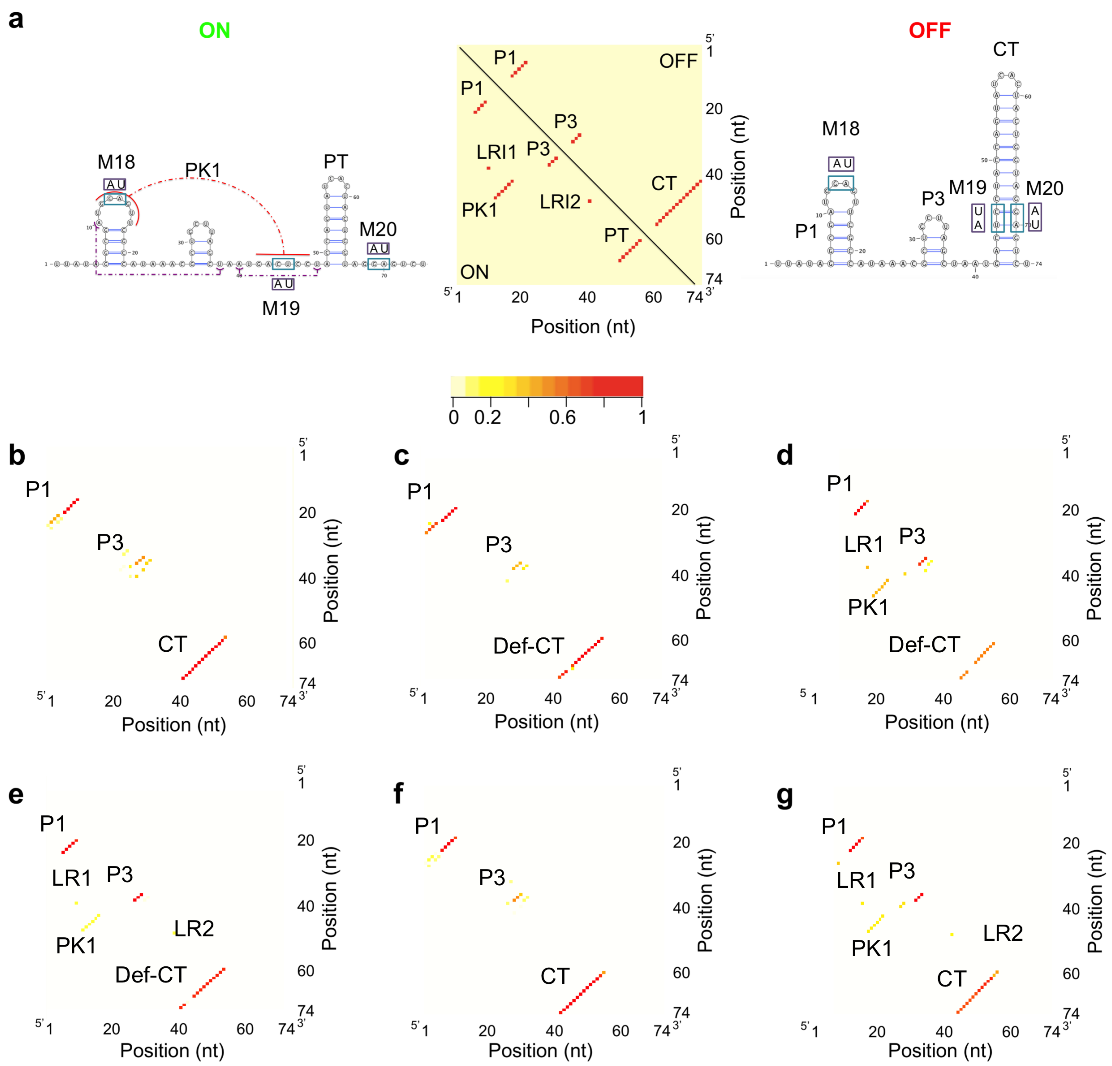

Supplementary Figure 8: **PPP visualization of analyses of *crcB* fluoride riboswitch mutants data at length 74 nt in the presence of ligand.** **(a)** ON and OFF structures with their corresponding PPPs. The locations of M18, M19 and M20 mutations on two structures are shown. M21 is the combination of M18 and M19, M22 is the combination of M19 and M20, and M23 is a combination of M18, M19 and M20. **(b)** M18. Pseudoknot PK1 was fully disrupted, due to M18 being located on base pairs forming PK1. **(c)** M19. PK1 disappeared while CT was affected on several base pairs. **(d)** M20. Only CT was affected and PK1's relative abundance was 43%. **(e)** M21. PK1 was restored and its population fraction was 18%. **(f)** M22. PK1 was disrupted completely while CT was restored. **(g)** M23. Both CT and PK1 were restored. PK1's relative abundance was 29%.

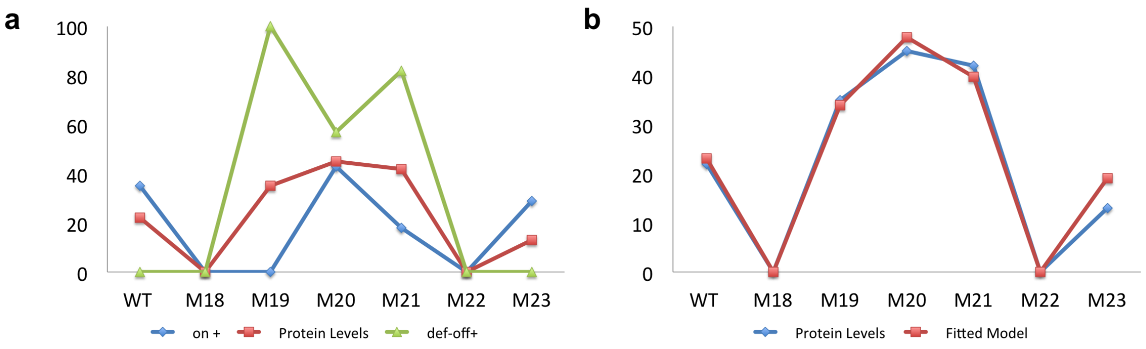

Supplementary Figure 9: **Two structural contributors to protein synthesis.** **(a)** Abundances of ON+ and DEF-OFF+ structural clusters estimated by SLEQ and reporter gene fusion measurements from [2] in the presence of fluoride. **(b)**Fitting of two-variable linear model to gene activity measurements yields a near-perfect match.

.

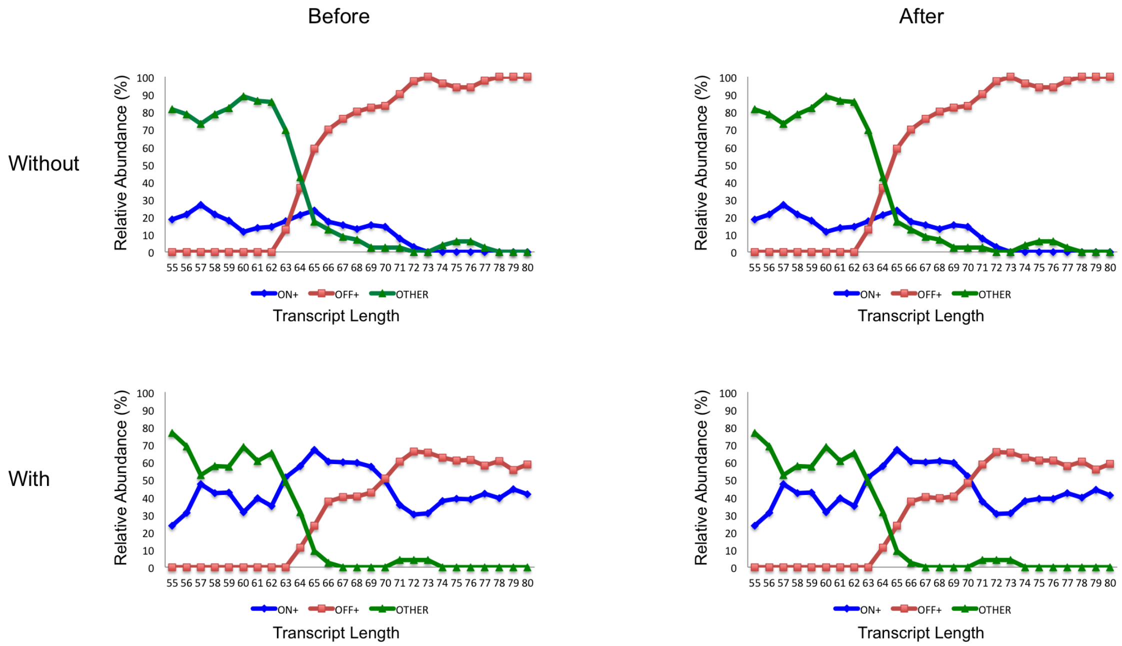

Supplementary Figure 10: **Abundances of ON+, OFF+ and OTHER clusters with and without ligand, depending on intermediate elongating transcript length for *crcB* fluoride riboswitch.** The first and second columns show the results before and after spiking-in structures with LR1. There is no observable difference between results.

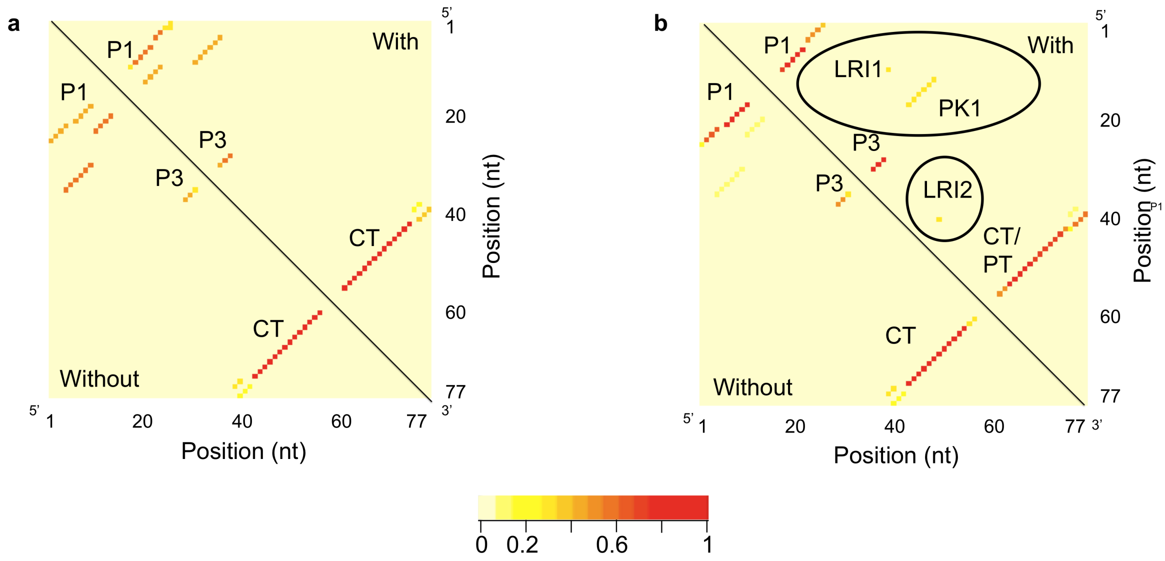

Supplementary Figure 11: **Analyses of the fluoride riboswitch cotranscriptional and equilibrium datasets at 77 nt in the presence and absence of ligand.** **(a)** PPP of results at equilibrium conditions. The OFF structure (P1, P3 and CT) dominates both with and without fluoride. **(b)** PPP of results during transcription. The OFF structure dominates in the absence of ligand. However, with the ligand, both OFF and ON (P1, P3, PK1, LR and PT) structures are present. This suggests that cotranscriptional folding is critical to the formation of the ligand-dependent ON structure.

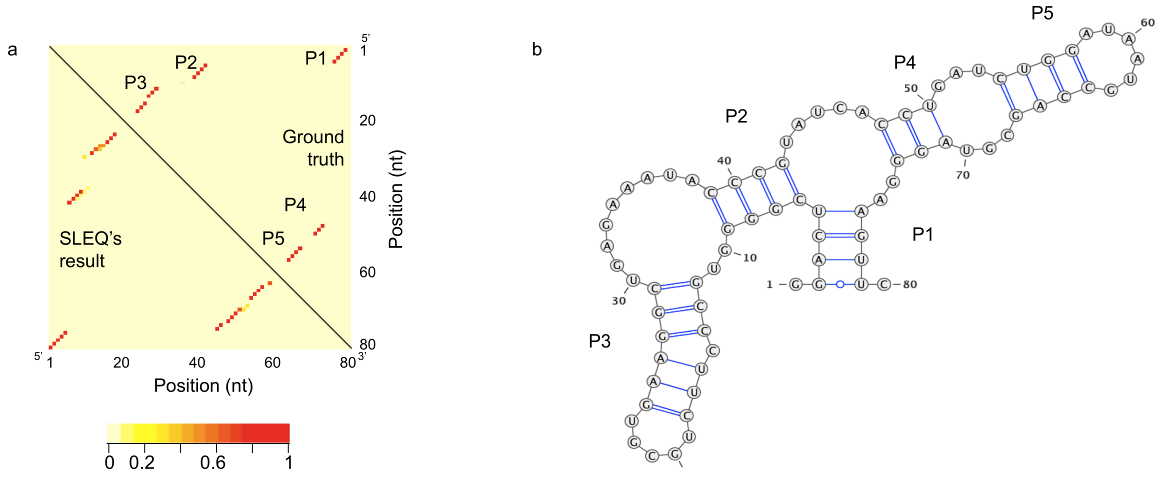

Supplementary Figure 12: **SLEQ recovers *thiM* TPP riboswitch aptamer domain in the presence of ligand.** **(a)** Compared to the ground truth, SLEQ recovers all main helices accurately. **(b)** The ground truth is the ligand-bound secondary structure of *thiM* TPP riboswitch aptamer domain[3].

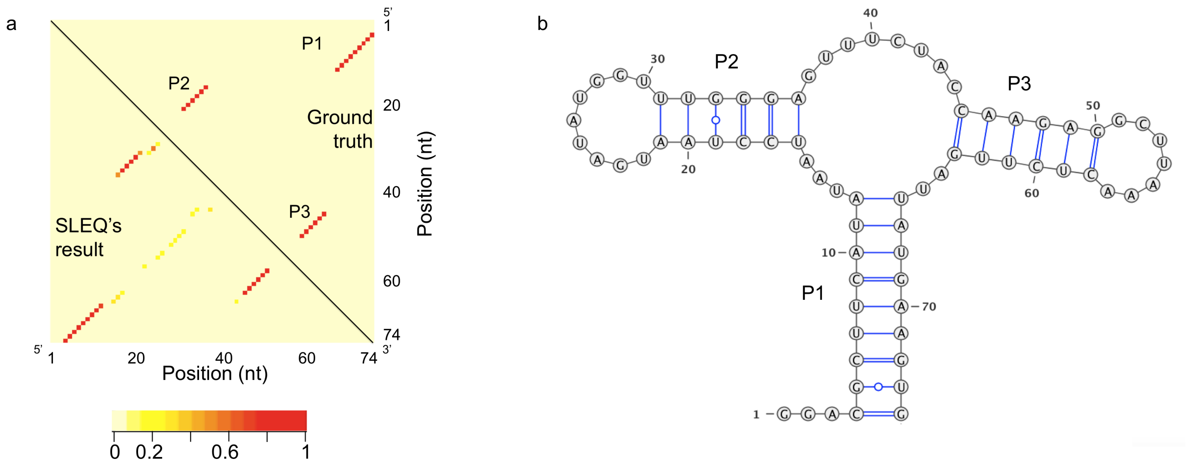

Supplementary Figure 13: **SLEQ recovers *add* adenine riboswitch aptamer domain in the presence of ligand. (a)** Compared to the ground truth, SLEQ is able to successfully reconstruct the main helices P1, P2 and P3. **(b)** The ground truth is the ligand-bound secondary structure of *add* adenine riboswitch aptamer domain[4].

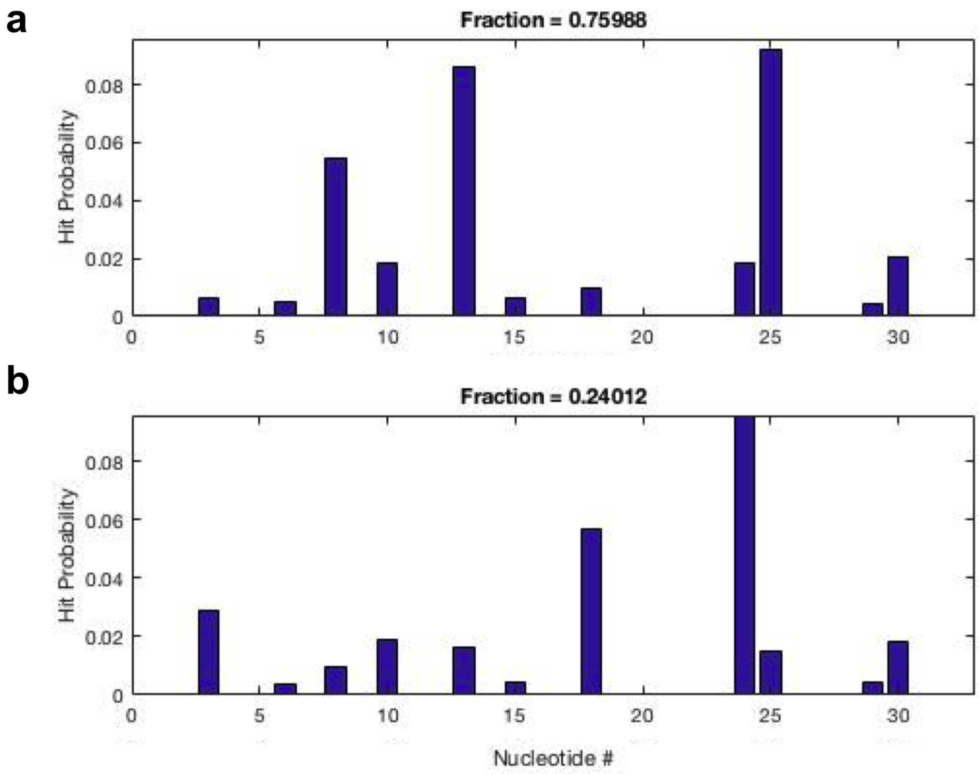

Supplementary Figure 14: **Results of the human *MRPS21* riboSNitch analyzed by RING-MaP. (a, b)** Reactivity profiles and population fractions of two clusters. The top cluster was consistent with allele A and the bottom cluster was consistent with allele C.

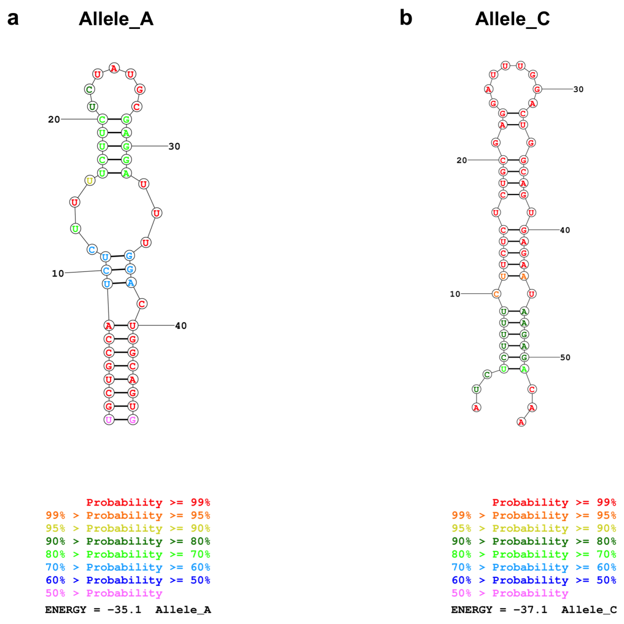

Supplementary Figure 15: **Structures predicted for alleles A and C by RNAstructure[5] based on RING-MaP's clustered reactivity profiles (shown in Supplementary Fig. 14). (a)** Structure prediction for Allele A. **(b)** Structure pediction for Allele C.

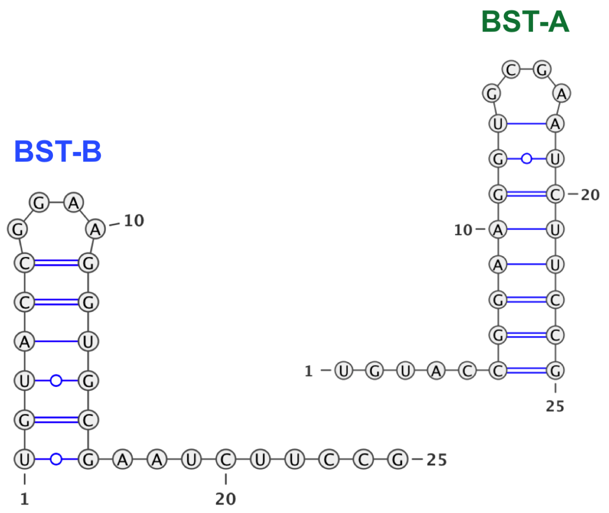

Supplementary Figure 16: **BST is a 25-nt synthetic RNA which adopts two competing hairpins, BST-A and BST-B.**

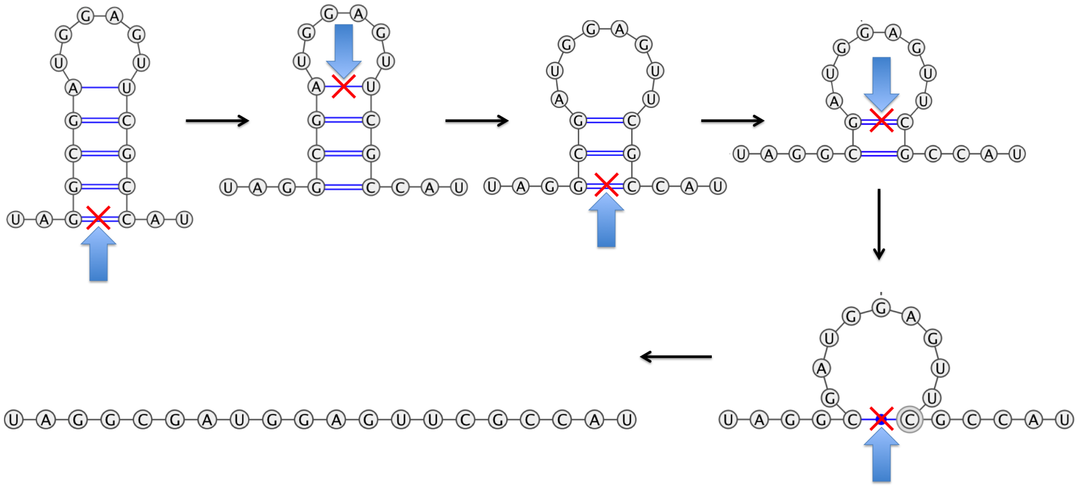

Supplementary Figure 17: **Divergence of the P1 helix from both ends towards the center in a zipper-like fashion.**

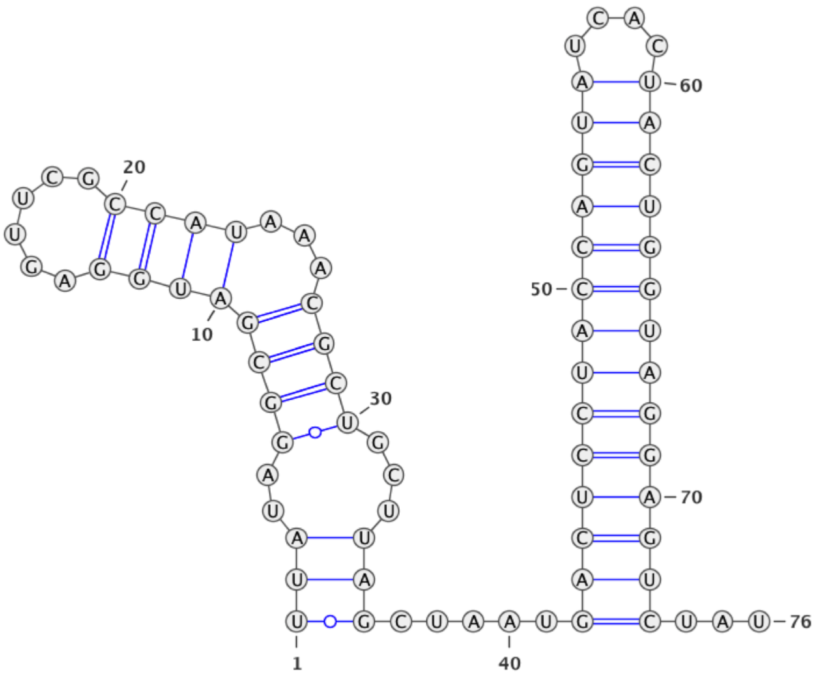

Supplementary Figure 18: **An OTHER structure of *crcB* fluoride riboswitch at length 76 nt.** Though it has CT motif, the absence of P1 motif results in its OTHER cluster classification.

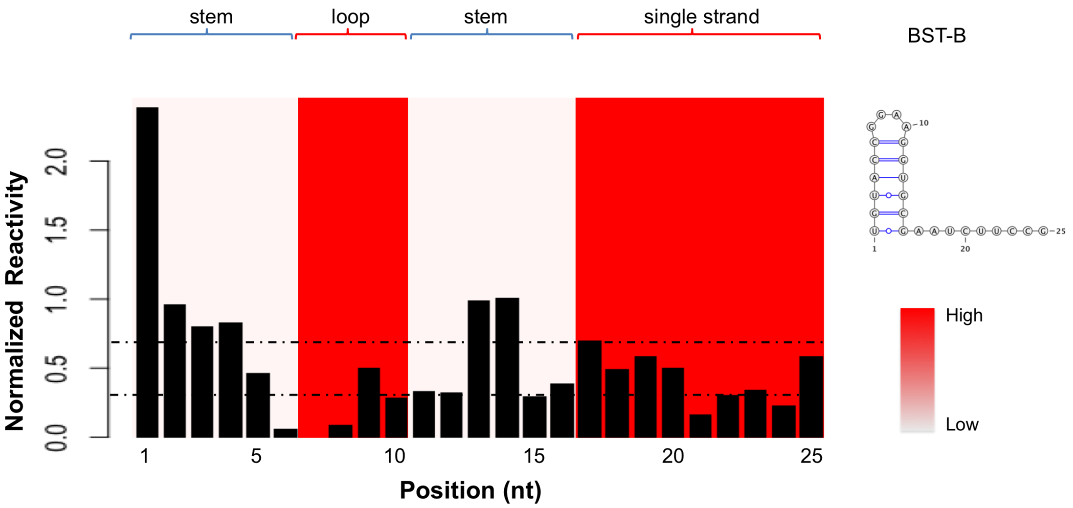

Supplementary Figure 19: **Comparison between the expected reactivity and the real profile for BST RNA when BST-B is the only candidate.** To mimic the scenario in which important structures were missing from the candidate set, we considered the case in which BST-B was the only candidate, meaning the important structure BST-A was missing. We compared the reactivity profile we expected to see in such a case to BST's real profile and detected mismatches. The expected reactivity profile was constructed as follows: for constrained nucleotides in BST-B, the expected reactivity values should be low (colored in white); for unconstrained nucleotides in BST-B, the expected reactivity values should be high (colored in red). The real profile (black bars) was normalized using 2%-8%[6] method (shown overlaid on the expected profile). Two dashed horizontal lines denote low/medium/high reactivity regions commonly used when interpreting normalized reactivities. We observed that despite being paired in BST-B, nucleotides $1-4$, and $13-14$ were highly reactive. This suggests that BST-B alone could not explain the real data. Therefore, we infer that certain key structures containing unpaired nucleotides on some of these sites were missing from the candidate set.

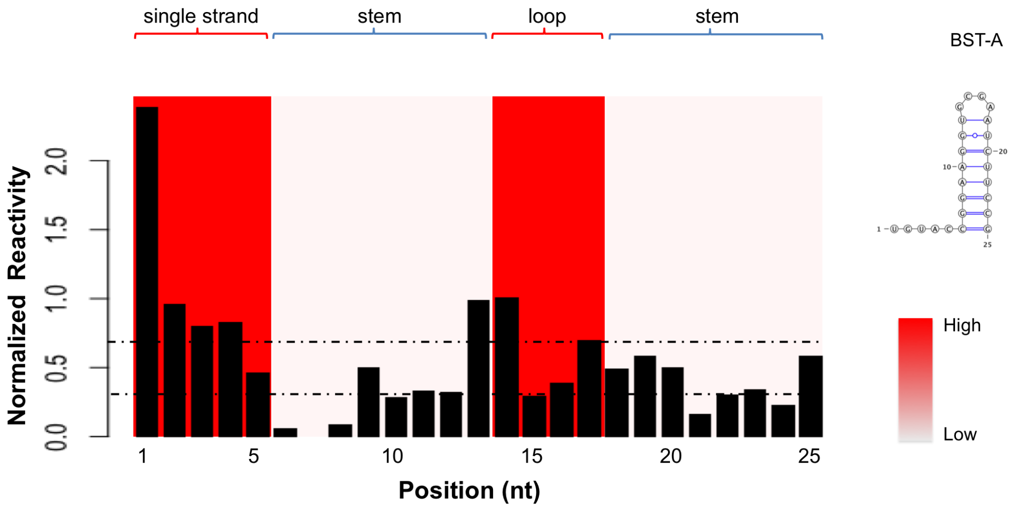

Supplementary Figure 20: **Comparison between the expected reactivity and the real profile for BST RNA when BST-A is the only candidate.** We considered the case in which BST-A was the only candidate, meaning the important structure BST-B was missing. We compared the reactivity profile we expected to see in such a case to BST's real profile and detected mismatches. We constructed expected reactivity profile and normalized real reactivity profile (black bars) as described in **Supplementary Figure 19**. We observed that nucleotides 9, 13, 18 – 20 and 25 were reactive but they were paired in BST-A. This suggested that BST-A alone could not explain the real data.

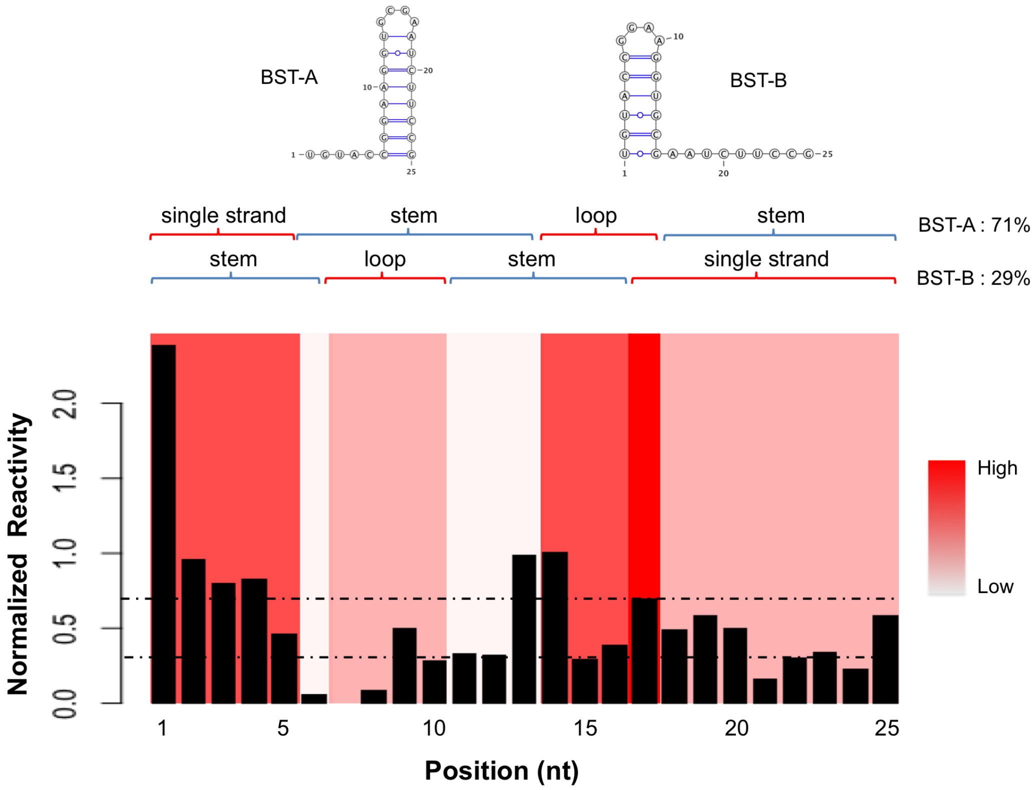

Supplementary Figure 21: **Comparison between the expected reactivity and the real profile for the BST RNA when both BST-A and BST-B coexist in the candidate set.** We include both the BST-A and BST-B structures into the candidate set and SLEQ estimated the population fractions of these two structures as BST-A:BST-B = 71%:29%. We compared the reactivity profile we expected to see in such a case to BST's real profile and detected mismatches. We constructed expected reactivity profile and normalized real reactivity profile (black bars) as described in **Supplementary Figure 19**. The expected profile was constructed as before, but with population fractions also taken into account. Specifically, if a nucleotide was unpaired in both BST-A and BST-B, it was 100% unpaired and its reactivity should be high (referred as η); if a nucleotide was unpaired in BST-A but paired in BST-B, it was 71% unpaired and its reactivity value should be 71% of η; if a nucleotide was paired in BST-A but unpaired in BST-B, it was 29% unpaired and its reactivity value should be 29% of η; if a nucleotide was paired in both structures, is was 0% unpaired and its reactivity value should be 0. By comparing the two profiles visually, we observed that most of highly reactive regions had reddish backgrounds, indicating an improved match.

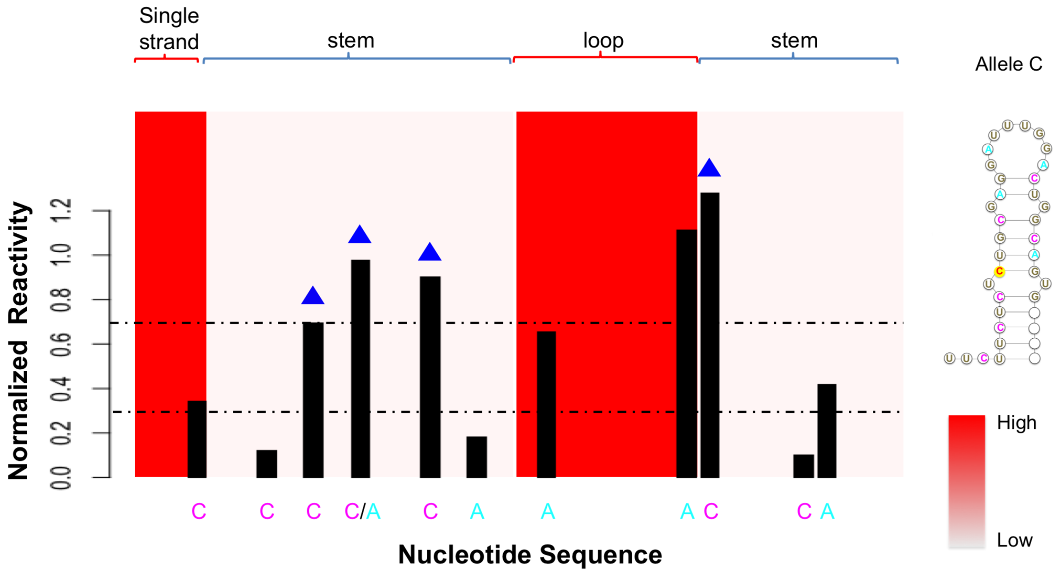

Supplementary Figure 22: **Comparison between the expected reactivity and the real profile for the *MRPS21* human riboSNitch when allele A is missing.** We removed allele A and all its variants from the candidate set and compared the reactivity profile we expect to see in such a case to the real profile to detect mismatches. We constructed the expected reactivity profile and normalized the real reactivity profile (black bars) as described in **Supplementary Figure 19**. We observed that the nucleotides marked by blue triangles were highly reactive but expected to be low. This suggested that allele C alone was unable to suitably fit the real data.

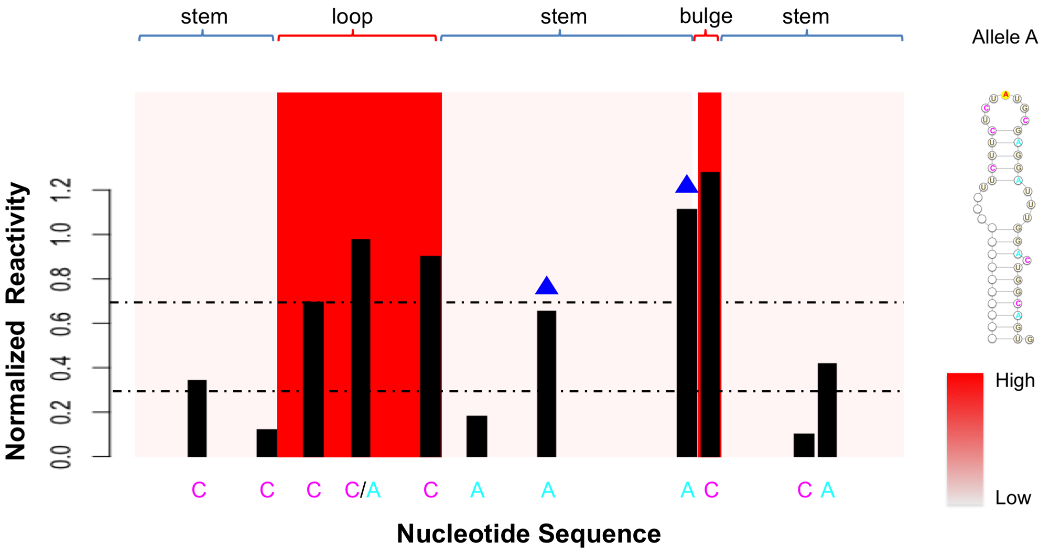

Supplementary Figure 23: **Comparison between the expected reactivity and the real profile for *MRPS21* human riboSNitch when allele C is missing.** We removed allele C and all its variants from the candidate set and compared the reactivity profile we expect to see in such case to the real profile to detect mismatches. We constructed the expected reactivity profile and normalized the real reactivity profile (black bars) as described in **Figure 19**. We observed that nucleotides marked by blue triangles were highly reactive but expected to be low. This suggested that allele A alone could not explain the real data.

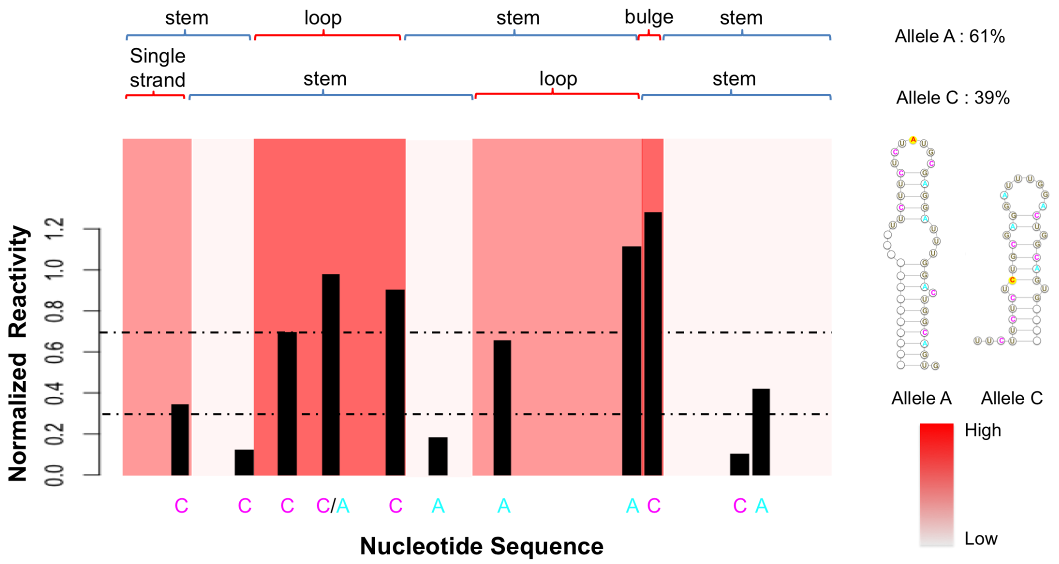

Supplementary Figure 24: **Comparison between the expected reactivity and the real profile for the *MRPS21* human riboSNitch RNA when both allele A and allele C coexist in the candidate set.** We include both structures for alleles A and C in the candidate set and SLEQ estimated population fractions of these two structures A:C = 61%:39%. We compared the reactivity profile we expect to see in such a case to the real profile to detect mismatches. We constructed the expected reactivity profile and normalized the real reactivity profile (black bars) as described in **Figure 21**. By comparing two profiles visually, we observed that most of highly reactive regions had reddish backgrounds, indicating an improved match.

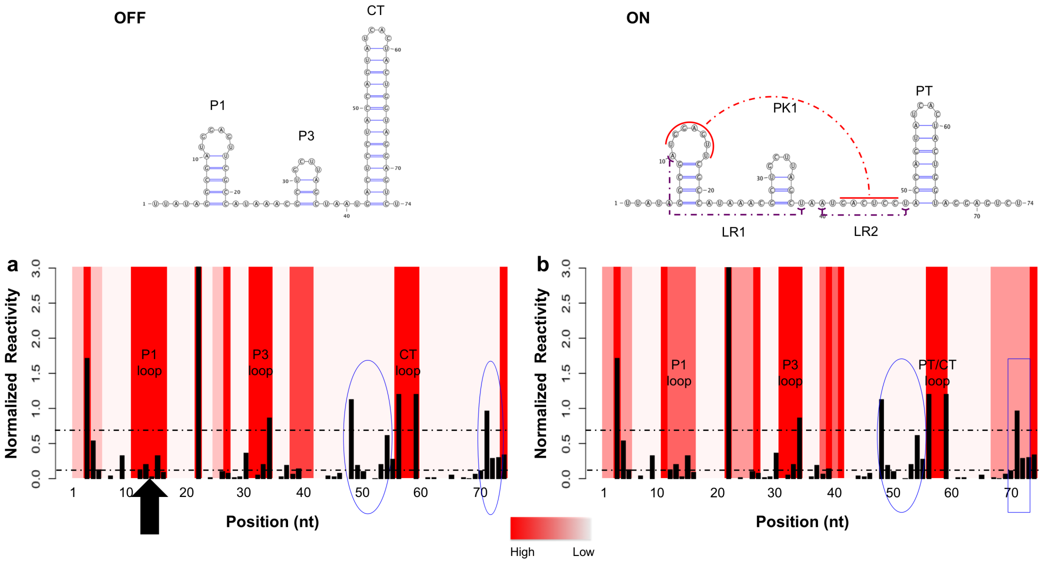

Supplementary Figure 25: **Comparison between the expected reactivity and the real profiles for *crcB* fluoride riboswitch at 76nt length in presence of ligand before and after including ON structure.** **(a)** Before including ON structure into the candidate set, we compared the reactivity profile we expect to see in such a case to the real profile to detect mismatches. We applied same methods to construct expected reactivity and normalized the real profile (black bars) described in **Supplementary Figure 21**. By comparing the two profiles visually, we observed that the two mismatch regions marked by ovals where real reactivity values were medium-to-high but expected to be low. We inferred that certain important structures with unpaired nucleotides in some of these regions were missing. **(b)** After including ON into the candidate set, population fractions estimated by SLEQ were ON+:OFF+ = 39%:61%. We constructed expected reactivity profile and normalized real reactivity profile (black bars) as described in **Supplementary Figure 21**. We observed that one of these two mismatches was fixed marked by rectangular, since nucleotides in this region in ON structure were unpaired.

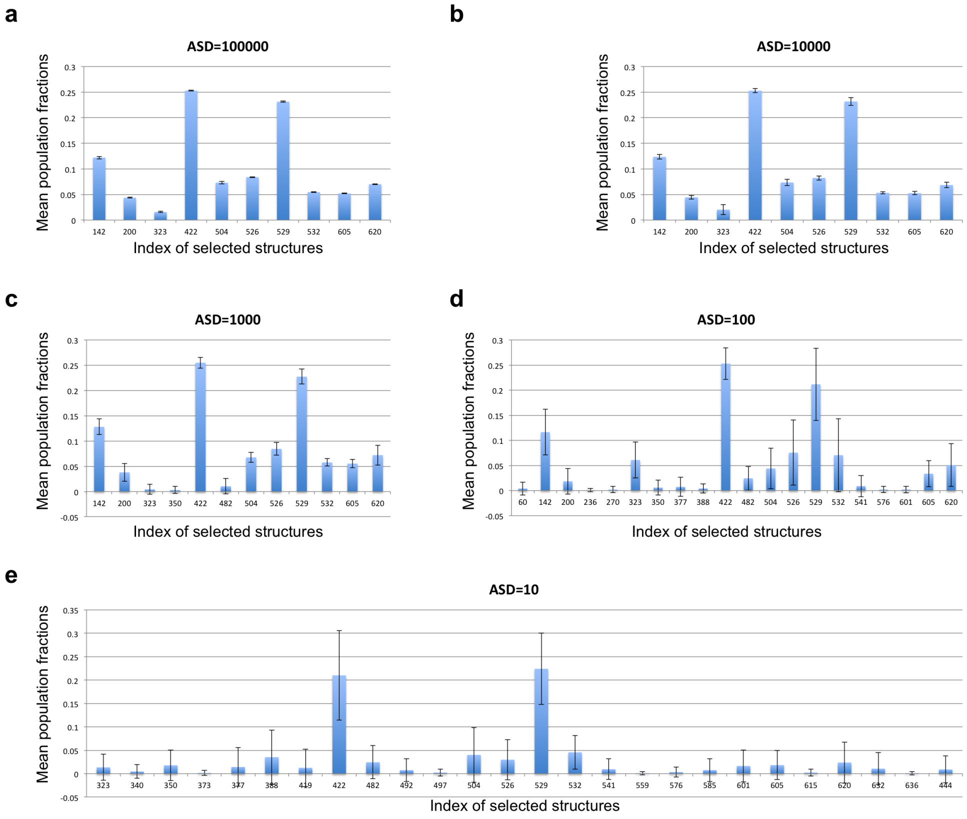

Supplementary Figure 26: **SLEQ's reconstruction on** 10 **independent simulated libraries of various average sequencing depths (ASD) for *E. coli* 16S ribosomal RNA.** Mean population fractions for selected structures are shown. **(a)** ASD=10^5. **(b)** ASD=10^4. **(c)** ASD=10^3. **(d)** ASD=10^2. **(e)** ASD=10.

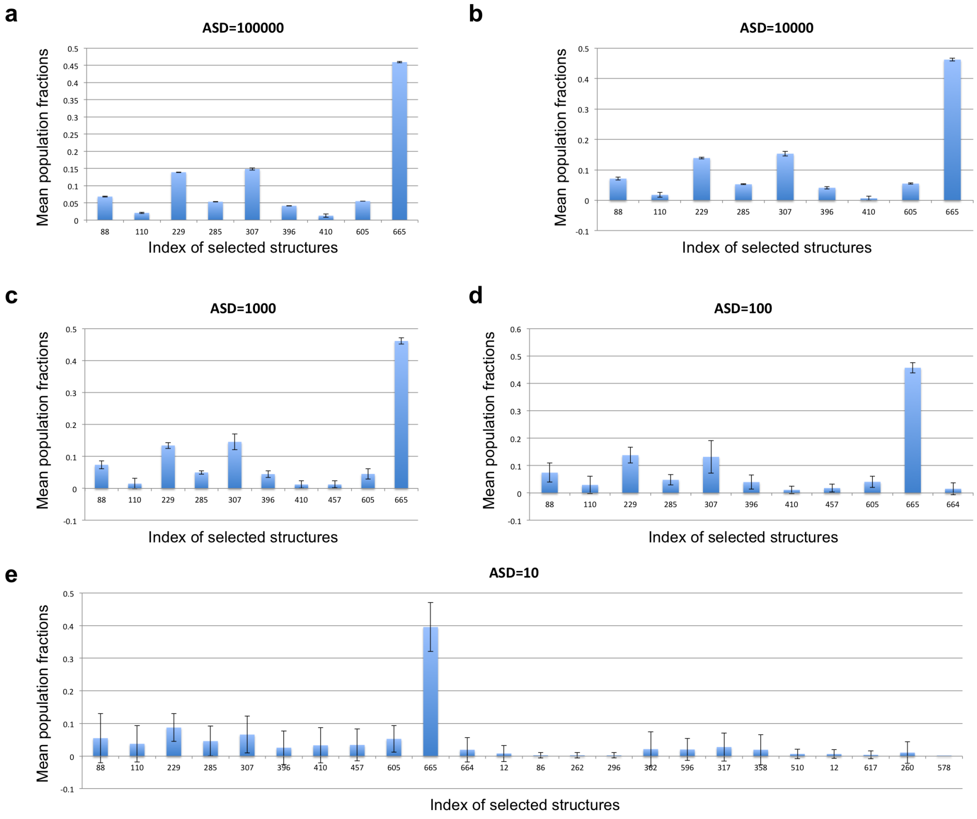

Supplementary Figure 27: **SLEQ's reconstruction on 10 independent simulated libraries of various average sequencing depths (ASD) for *add* RNA in the presence of ligand.** Mean population fractions for selected structures are shown. **(a)** ASD=10^5. **(b)** ASD=10^4. **(c)** ASD=10^3. **(d)** ASD=10^2. **(e)** ASD=10.

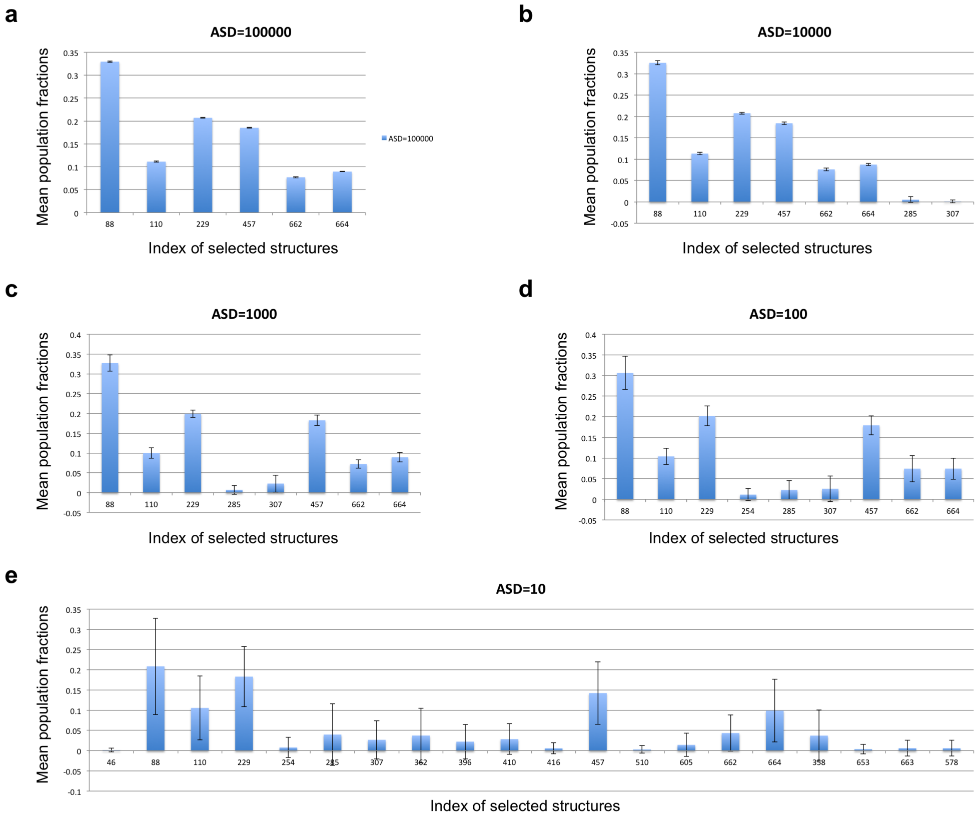

Supplementary Figure 28: **SLEQ's reconstruction on 10 independent simulated libraries of various average sequencing depths (ASD) for *add* RNA in the absence of ligand.** Mean population fractions for selected structures are shown. **(a)** ASD=10^5. **(b)** ASD=10^4. **(c)** ASD=10^3. **(d)** ASD=10^2. **(e)** ASD=10.

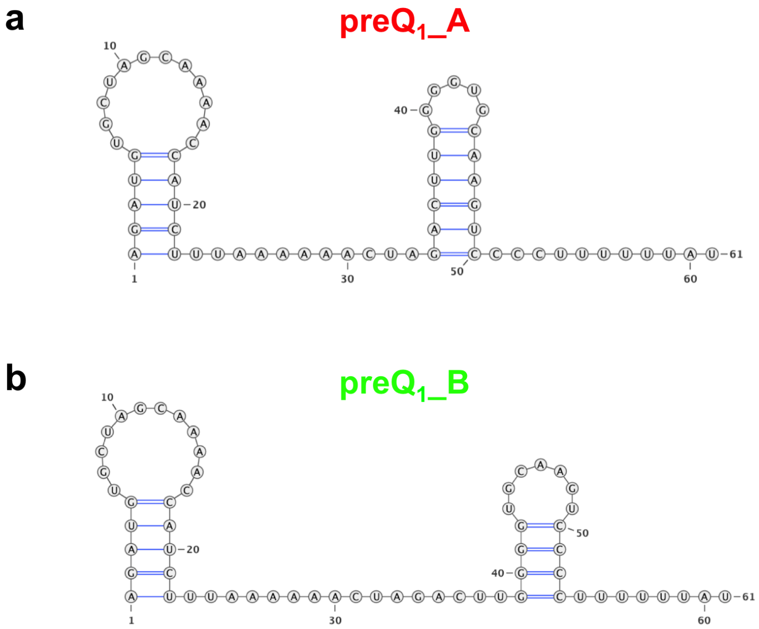

Supplementary Figure 29: **Two competing structures of the preQ$_1$ responsive RNA element from *Fusobacterium nucleatum*, as shown in [7]. (a)** preQ$_1$_A. **(b)** preQ$_1$_B.

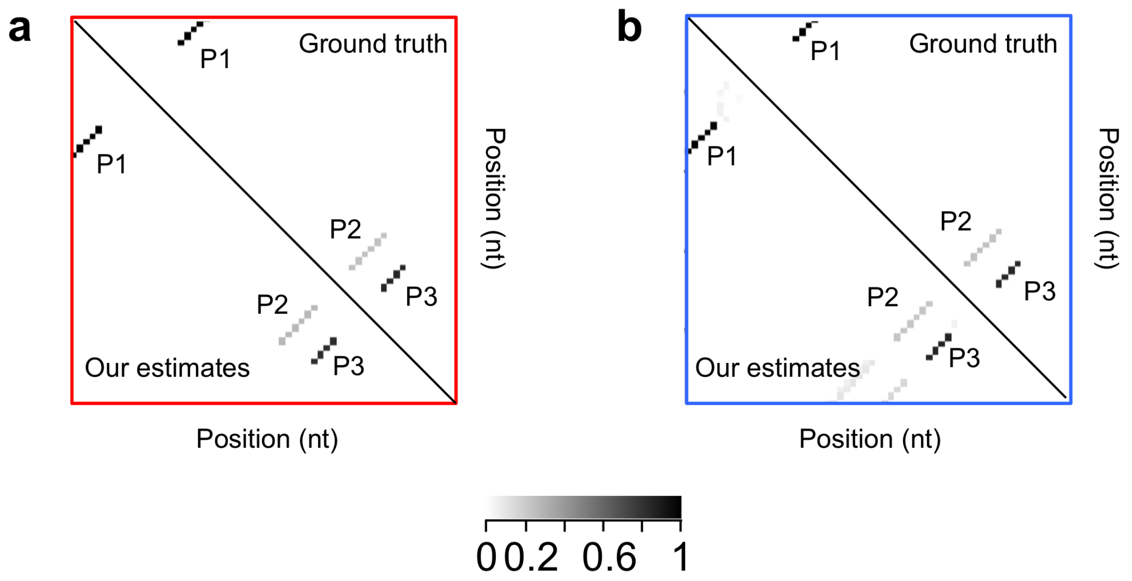

Supplementary Figure 30: **Comparisons of ground truth base-pairing probabilities (above diagonal) to SLEQ's estimates (below diagonal) at 10dB noise level in both modes.** **(a)** Mutation mode. **(b)** Truncation mode. The main helices - P1, P2, P3 - were recovered. Gray level indicates base-pairing probability.

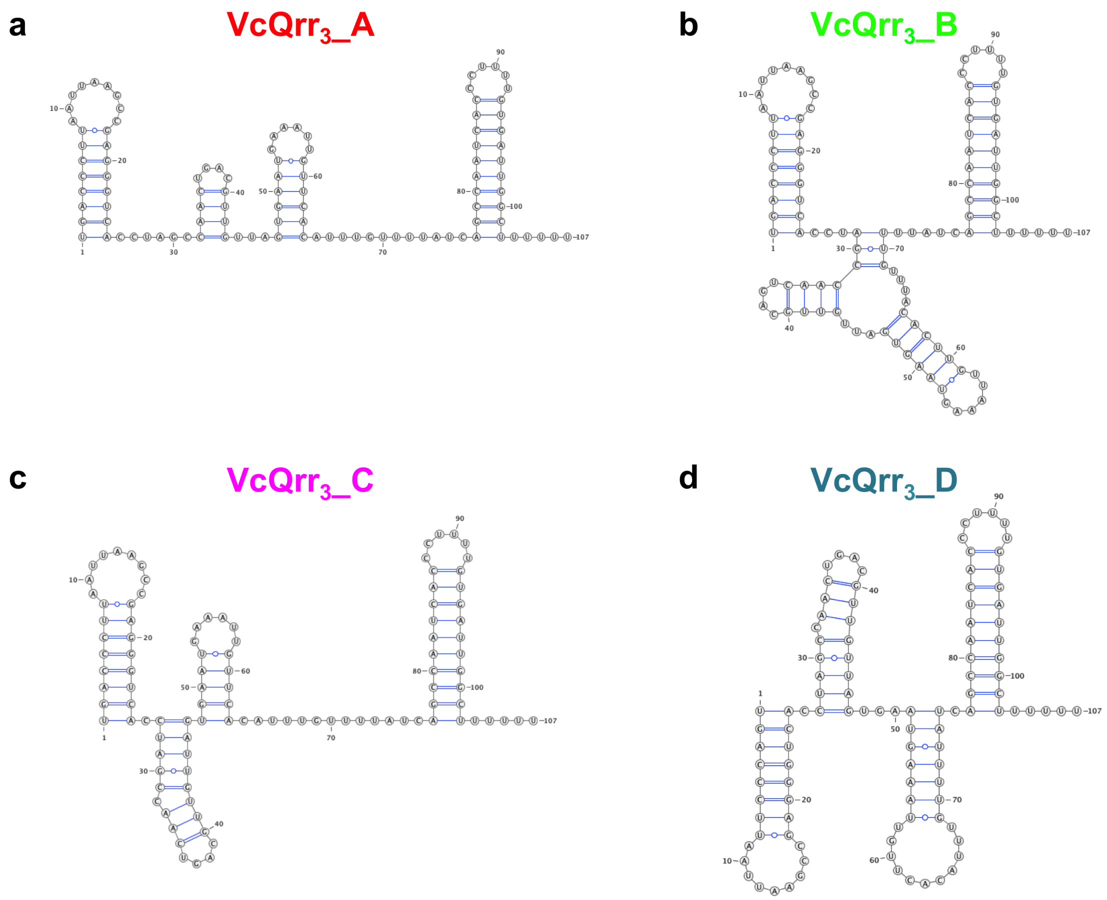

Supplementary Figure 31: **Four alternative structures of VcQrr$_3$ RNA from V. *cholerae*, as predicted in [8].** **(a)** VcQrr$_3$_A. **(b)** VcQrr$_3$_B. **(c)** VcQrr$_3$_C. **(d)** VcQrr$_3$_D.

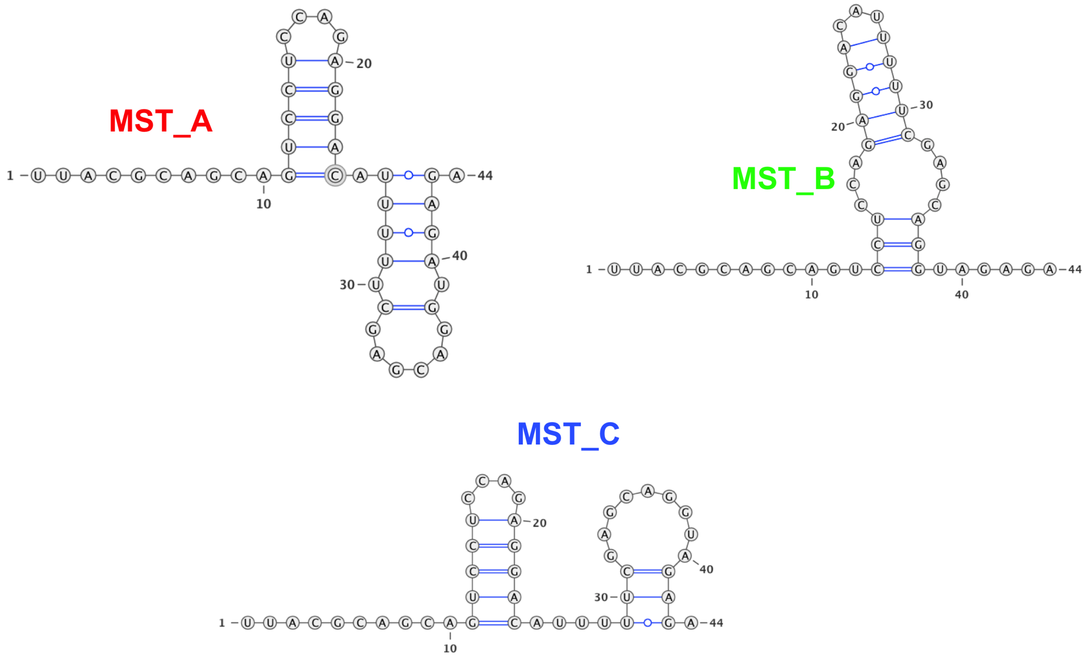

Supplementary Figure 32: **Three alternative structures of M-stable RNA (MST), as predicted in [9].**

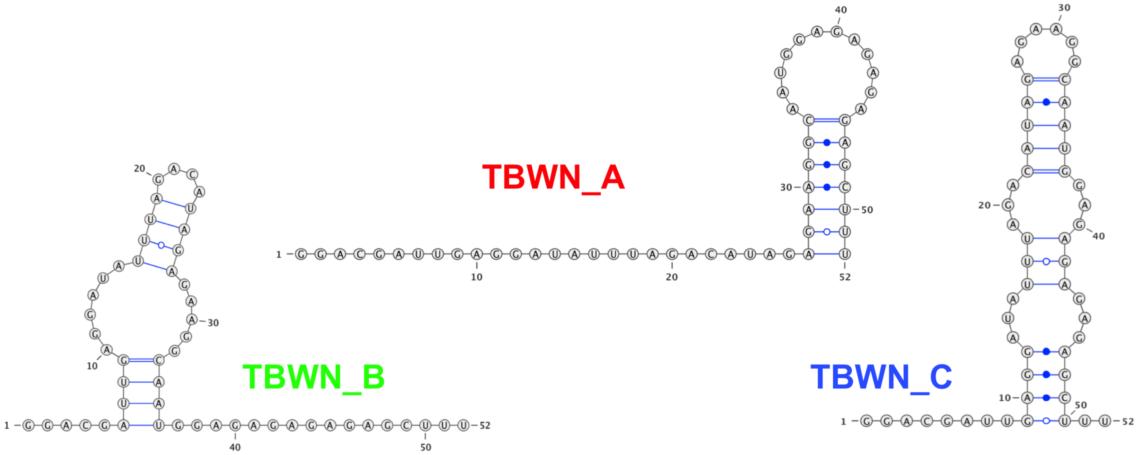

Supplementary Figure 33: **Three alternative structures of Tebowned (TBWN) RNA, as predicted in [9].**

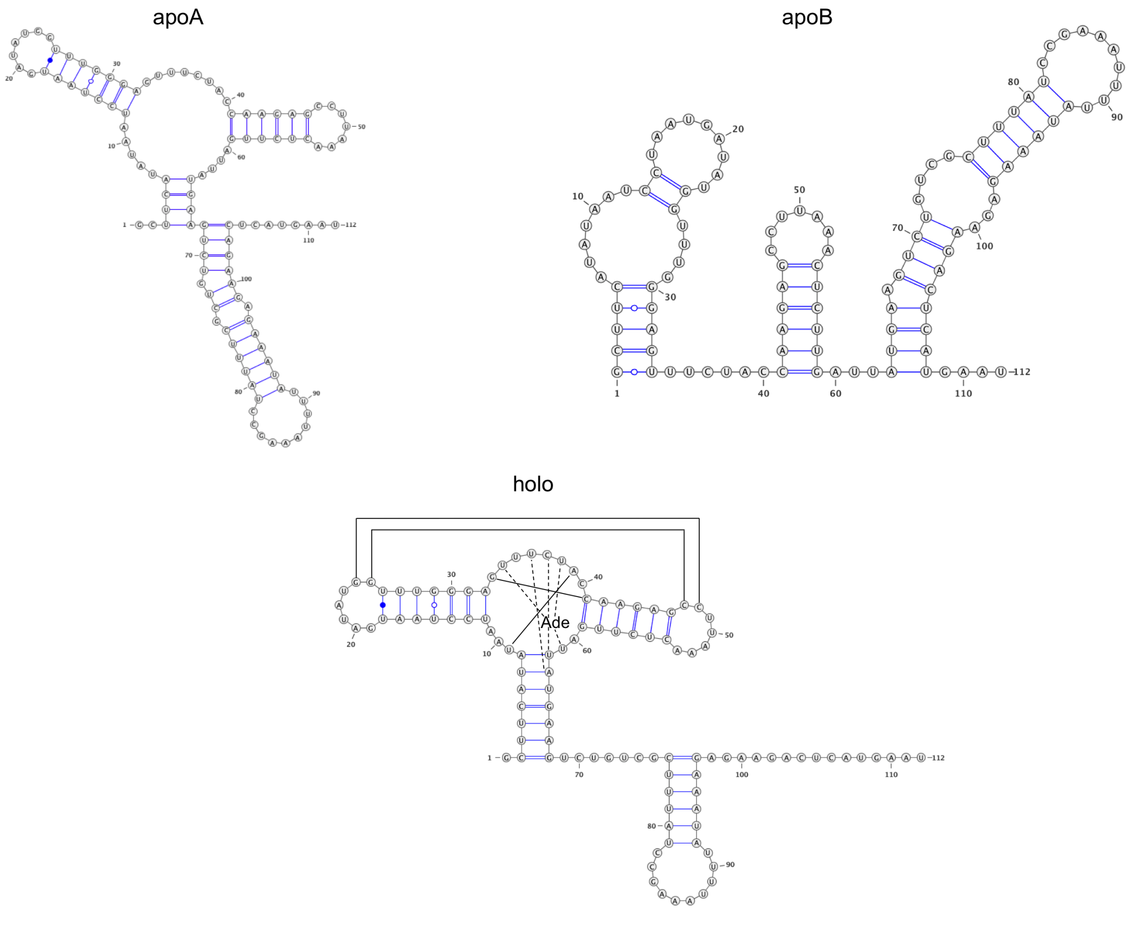

Supplementary Figure 34: **Three alternative structures of *add* RNA[10].**

2 SUPPLEMENTARY TABLES

		Truncation		Mutation	
Run	Number of Candidates	$\hat{\rho}$	Number of selected	$\hat{\rho}$	Number of selected
1	105	0.67 : 0.33	5	0.62 : 0.38	5
2	109	0.62 : 0.38	4	0.60 : 0.40	4
3	114	0.61 : 0.39	5	0.59 : 0.41	5
4	101	0.60 : 0.40	6	0.63 : 0.37	4
5	107	0.62 : 0.38	6	0.54 : 0.46	5
6	104	0.60 : 0.40	5	0.58 : 0.42	5
7	114	0.59 : 0.41	4	0.54 : 0.46	5
8	111	0.61 : 0.39	5	0.64 : 0.36	6
9	115	0.62 : 0.38	5	0.54 : 0.46	6
10	114	0.60 : 0.40	4	0.61 : 0.39	4
mean		0.62 : 0.38	5	0.59 : 0.41	5
sd		0.020		0.038	

Supplementary Table 1: **SLEQ analysis results for the *MRPS21* human riboSNitch DMS-MaPseq data.** In each of the 10 SLEQ runs, the candidate structure set was independently sampled using the ViennaRNA package[11].

		Absence of fluoride		Presence of fluoride	
Transcript length	Number of candidates	Number of selected	ON+:OFF+:OTHER	Number of selected	ON+:OFF+:OTHER
55	240	8	0.20 : 0.00 : 0.80	7	0.15 : 0.00 : 0.85
56	263	7	0.17 : 0.00 : 0.83	6	0.32 : 0.00 : 0.68
57	265	6	0.27 : 0.00 : 0.73	4	0.46 : 0.00 : 0.54
58	313	8	0.37 : 0.00 : 0.63	5	0.64 : 0.00 : 0.36
59	291	8	0.00 : 0.00 : 1.00	6	0.17 : 0.00 : 0.83
60	316	7	0.17 : 0.00 : 0.83	6	0.47 : 0.00 : 0.53
61	345	9	0.17 : 0.00 : 0.83	7	0.30 : 0.00 : 0.70
62	368	10	0.07 : 0.00 : 0.93	6	0.41 : 0.00 : 0.59
63	359	10	0.19 : 0.00 : 0.81	7	0.34 : 0.00 : 0.66
64	178	11	0.27 : 0.38 : 0.35	4	0.79 : 0.00 : 0.21
65	145	9	0.17 : 0.71 : 0.12	7	0.60 : 0.33 : 0.07
66	161	8	0.27 : 0.68 : 0.05	5	0.62 : 0.38 : 0.00
67	175	10	0.08 : 0.71 : 0.21	3	0.59 : 0.41 : 0.00
68	136	10	0.11 : 0.89 : 0.00	4	0.59 : 0.41 : 0.00
69	118	8	0.20 : 0.80 : 0.00	4	0.64 : 0.36 : 0.00
70	148	10	0.15 : 0.78 : 0.07	5	0.56 : 0.44 : 0.00
71	118	11	0.08 : 0.92 : 0.00	4	0.36 : 0.64 : 0.00
72	140	8	0.00 : 1.00 : 0.00	5	0.20 : 0.68 : 0.12
73	110	7	0.00 : 1.00 : 0.00	5	0.35 : 0.65 : 0.00
74	103	5	0.00 : 1.00 : 0.00	2	0.37 : 0.63 : 0.00
75	114	9	0.00 : 0.89 : 0.11	3	0.41 : 0.59 : 0.00
76	141	8	0.00 : 0.93 : 0.07	2	0.39 : 0.61 : 0.00
77	170	8	0.00 : 1.00 : 0.00	5	0.37 : 0.63 : 0.00
78	168	7	0.00 : 1.00 : 0.00	4	0.51 : 0.49 : 0.00
79	197	3	0.00 : 1.00 : 0.00	6	0.31 : 0.69 : 0.00
80	168	8	0.00 : 1.00 : 0.00	3	0.51 : 0.49 : 0.00

Supplementary Table 2: **SLEQ analysis results for the *crcB* fluoride riboswitch SHAPE-Seq data at lengths** $55-80$ **nt in the presence and absence of fluoride.** A structure is selected if its estimated abundance exceeds 1%. Descriptions of ON+, OFF+, and OTHER structural clusters can be found in the main text.

Sequence	PK1	CT
WT	✓	✓
M18	×	✓
M19	×	×
M20	✓	×
M21	✓	×
M22	×	✓
M23	✓	✓

Supplementary Table 3: **Effects of mutations on structural motifs that are key to transcription termination in the presence of fluoride.** In WT, both PK1 and CT exist. In M18, PK1 is disrupted. In M19, both PK1 and CT are disrupted. In M20, only CT is disrupted. In M21, PK1 is restored but CT is disrupted. In M21, CT is restored while PK1 is disrupted. In M23, both PK1 and CT are restored.

Data	Profiling technique	Repository	Organism	Condition	Sequencing	Reference
MRPS21 riboSNitch alleles	DMS-MaPseq	GEO (accession code GSE84537)	Human	*in vitro*	single-end	Zubradt *et al.*, 2016
crcB fluoride riboswitch	SHAPE-Seq	RNA Mapping Database[12] FLUORSW_BZCN_0001<br>FLUORSW_BZCN_0004<br>FLUORSW_BZCN_0007<br>FLUORSW_BZCN_0008<br>FLUORSW_BZCN_0009<br>FLUORSW_BZCN_0010<br>FLUORSW_BZCN_0011<br>FLUORSW_BZCN_0012<br>FLUORSW_BZCN_0013<br>FLUORSW_BZCN_0014<br>FLUORSW_BZCN_0015<br>FLUORSW_BZCN_0016<br>FLUORSW_BZCN_0017<br>FLUORSW_BZCN_0018<br>FLUORSW_BZCN_0019<br>FLUORSW_BZCN_0020	B. *cereus*	*in vitro*	paired-end	Watters *et al.*, 2016
Hobartner bistable switch (BST)	Mutate-and-Map	RNA Mapping Database BSTHPN_1M7_0000	Engineered			Cordero *et al.*, 2015
add adenine riboswitch (*add* RNA)	Mutate-and-Map	RNA Mapping Database ADDSCHW_1M7_0000<br>ADDSCHW_1M7_0001	V. *vulnificus*	*in vitro*		Cordero *et al.*, 2015
16S rRNA four-way junction (16S)	Mutate-and-Map	RNA Mapping Database 16SFWJ_1M7_0001	*E. coli*	*in vitro*		Tian *et al*, 2014
add adenine riboswitch aptamer domain (*add* aptamer)	SHAPE-Seq	RNA Mapping Database ADDSC_1M7_0008	V. *vulnificus*	*in vitro*	paired-end	Watters *et al.*, 2016
thiM TPP riboswitch aptamer domain (TPP)	SHAPE-Seq	RNA Mapping Database TPPSC_1M7_0005	E. *coli*	*in vitro*	paired-end	Watters *et al.*, 2016

Supplementary Table 4: **Datasets analyzed in this study.**

Sequences of *MRPS21* riboSNitch alleles:

Allele A: UGCUGCCAUCUCUUUUCUUCUCUAUGCGAGGAUUUGGACUGGCAGUG.

Allele C: AUCUCUUUUCUUCUCUCUGCGAGGAUUUGGACUGGCAGUGAGAAUAAGA-GACAA.

Sequences of *crcB* fluoride riboswitch intermediate transcripts analyzed in this study:

26 nt: UUAUAGGCGAUGGAGUUCGCCAUAAA

41 nt: UUAUAGGCGAUGGAGUUCGCCAUAAACGCUGCUUAGCUAAU

55 nt: UUAUAGGCGAUGGAGUUCGCCAUAAACGCUGCUUAGCUAAUGACUCCUACCAGUA

56 nt: UUAUAGGCGAUGGAGUUCGCCAUAAACGCUGCUUAGCUAAUGACUCCUACCAGUAU

57 nt: UUAUAGGCGAUGGAGUUCGCCAUAAACGCUGCUUAGCUAAUGACUCCUACCAGUAUC

58 nt: UUAUAGGCGAUGGAGUUCGCCAUAAACGCUGCUUAGCUAAUGACUCCUACCAGUAUCA

59 nt: UUAUAGGCGAUGGAGUUCGCCAUAAACGCUGCUUAGCUAAUGACUCCUACCAGUAU-CAC

60 nt: UUAUAGGCGAUGGAGUUCGCCAUAAACGCUGCUUAGCUAAUGACUCCUACCAGUAU-CACU

61 nt: UUAUAGGCGAUGGAGUUCGCCAUAAACGCUGCUUAGCUAAUGACUCCUACCAGUAU-CACUA

62 nt: UUAUAGGCGAUGGAGUUCGCCAUAAACGCUGCUUAGCUAAUGACUCCUACCAGUAU-CACUAC

63 nt: UUAUAGGCGAUGGAGUUCGCCAUAAACGCUGCUUAGCUAAUGACUCCUACCAGUAU-CACUACU

64 nt: UUAUAGGCGAUGGAGUUCGCCAUAAACGCUGCUUAGCUAAUGACUCCUACCAGUAU-CACUACUG

65 nt: UUAUAGGCGAUGGAGUUCGCCAUAAACGCUGCUUAGCUAAUGACUCCUACCAGUAU-CACUACUGG

66 nt: UUAUAGGCGAUGGAGUUCGCCAUAAACGCUGCUUAGCUAAUGACUCCUACCAGUAU-CACUACUGGU

67 nt: UUAUAGGCGAUGGAGUUCGCCAUAAACGCUGCUUAGCUAAUGACUCCUACCAGUAU-CACUACUGGUA

68 nt: UUAUAGGCGAUGGAGUUCGCCAUAAACGCUGCUUAGCUAAUGACUCCUACCAGUAU-CACUACUGGUAG

69 nt: UUAUAGGCGAUGGAGUUCGCCAUAAACGCUGCUUAGCUAAUGACUCCUACCAGUAU-CACUACUGGUAGG

70 nt: UUAUAGGCGAUGGAGUUCGCCAUAAACGCUGCUUAGCUAAUGACUCCUACCAGUAU-CACUACUGGUAGGA

71 nt: UUAUAGGCGAUGGAGUUCGCCAUAAACGCUGCUUAGCUAAUGACUCCUACCAGUAU-CACUACUGGUAGGAG

72 nt: UUAUAGGCGAUGGAGUUCGCCAUAAACGCUGCUUAGCUAAUGACUCCUACCAGUAU-CACUACUGGUAGGAGU

73 nt: UUAUAGGCGAUGGAGUUCGCCAUAAACGCUGCUUAGCUAAUGACUCCUACCAGUAU-CACUACUGGUAGGAGUG

74 nt: UUAUAGGCGAUGGAGUUCGCCAUAAACGCUGCUUAGCUAAUGACUCCUACCAGUAU-CACUACUGGUAGGAGUGU

75 nt: UUAUAGGCGAUGGAGUUCGCCAUAAACGCUGCUUAGCUAAUGACUCCUACCAGUAU-CACUACUGGUAGGAGUGUA

76 nt: UUAUAGGCGAUGGAGUUCGCCAUAAACGCUGCUUAGCUAAUGACUCCUACCAGUAU-CACUACUGGUAGGAGUGUAU

77 nt: UUAUAGGCGAUGGAGUUCGCCAUAAACGCUGCUUAGCUAAUGACUCCUACCAGUAU-CACUACUGGUAGGAGUGUAUU

78 nt: UUAUAGGCGAUGGAGUUCGCCAUAAACGCUGCUUAGCUAAUGACUCCUACCAGUAU-CACUACUGGUAGGAGUGUAUUU

79 nt: UUAUAGGCGAUGGAGUUCGCCAUAAACGCUGCUUAGCUAAUGACUCCUACCAGUAU-CACUACUGGUAGGAGUGUAUUUU

80 nt: UUAUAGGCGAUGGAGUUCGCCAUAAACGCUGCUUAGCUAAUGACUCCUACCAGUAU-CACUACUGGUAGGAGUGUAUUUUU

Sequence of BST:

25 nt: UGUACCGGAAGGUGCGAAUCUUCCG

Sequence of *add* RNA:

112 nt: GCUUCAUAUAAUCCUAAUGAUAUGGUUUGGGAGUUUCUACCAAGAGCCU-UAAACUCUUGAUUAUGAAGUCUGUCGCUUUAUCCGAAAUUUUAUAAAGAGAAGAC-UCAUGAAU

Sequence of 16S:

110 nt: GGGAAACUGCCUGAUGGAGGGGGAUAACUACUGGAAACGGUAGCUAAUACCG-CAUAACGUCGCAAGACCAAAGAGGGGGACCUUCGGGCCUCUUGCCAUCGGAUGUGCCC

Sequence of *add* aptamer:

74 nt: GGACGCUUCAUAUAAUCCUAAUGAUAUGGUUUGGGAGUUUCUACCAAGAGC-CUUAAACUCUUGAUUAUGAAGUG

Sequence of TPP:

80 nt: GGACUCGGGGUGCCCUUCUGCGUGAAGGCUGAGAAAUACCCGUAUCACCU-GAUCUGGAUAAUGCCAGCGUAGGGAAGUUC

Supplementary Table 5: **Sequences analyzed in this study.**

Number of modifications per read	Number of reads
0	40472900
1	14335548
2	1846880
3	92437
4	2611
5	138
6	20

Supplementary Table 6: **Modification densities in the *MRPS21* human riboSNitch DMS-MaPseq data.** Modifications were counted only for adenines and cytosines within the 33 nt region of interest.

	Population fractions
Ground Truth	67.8% : 32.2%
SLEQ	62% : 38%
RING-MaP	76% : 24%

Supplementary Table 7: **Comparison of population fractions estimated by SLEQ and RING-MaP for *MRPS21* human riboSNitch DMS-MaPseq data.**

Run	Population fractions BST-A : BST-B
1	70.9% : 29.1%
2	70.7% : 29.3%
3	70.7% : 29.3%
4	71.2% : 28.8%
5	71.1% : 28.9%
6	71.0% : 29.0%
7	71.1% : 28.9%
8	70.9% : 29.1%
9	70.8% : 29.2%
10	70.7% : 29.3%
mean	70.9% : 29.1%
std	0.2%

Supplementary Table 8: **Population fractions estimated by SLEQ for BST RNA based on 10 simulated read datasets drawn from the real reactivity profile.**

	Population fractions BST-A : BST-B
NMR	$70 \pm 5\%$: $30 \pm 5\%$
SLEQ	$71 \pm 0.2\%$: $29 \pm 0.2\%$
M^2-REEFFIT	$73 \pm 11\%$: $26 \pm 9\%$

Supplementary Table 9: **Comparison of population fractions estimated by SLEQ and M^2-REEFFIT for BST RNA.**

	P1	P2	P3	P4	P4B	P5
NMR	30%	30%	100%	30%	70%	100%
SLEQ	$26 \pm 0.2\%$	$65 \pm 0.1\%$	$89 \pm 0.1\%$	$8 \pm 0.2\%$	$74 \pm 0.2\%$	$79 \pm 0.1\%$
M^2-REEFFIT	$14 \pm 10\%$	$37 \pm 15\%$	$86 \pm 18\%$	$69 \pm 10\%$	$36 \pm 10\%$	$95 \pm 18\%$

Supplementary Table 10: **The comparison of population fraction of helices estimated by SLEQ and M^2-REEFFIT for the *add* RNA.**

	P1a	P1b	P1c	P2b	P3	P4a	shift-P4a	alt-P1d	alt-P4
SLEQ	$69 \pm 0.1\%$	$20 \pm 0.0\%$	$96 \pm 0.2\%$	$100 \pm 0.0\%$	$85 \pm 0.2\%$	$0 \pm 0.0\%$	$14 \pm 0.2\%$	$32 \pm 0.1\%$	$2 \pm 0.1\%$
M^2-REEFFIT	$> 80\%$	$> 80\%$	$> 80\%$	$> 80\%$	$> 80\%$	$21 \pm 16\%$	$60 \pm 23\%$	low	low

Supplementary Table 11: **The comparison of population fraction of helices estimated by SLEQ and M^2-REEFFIT for the 126-235 region of the *E. coli* 16S ribosomal RNA.**

Run	P1a	P1b	P1c	P2b	P3	P4a	shift-P4a	alt-P1d	alt-P4
1	68.6%	19.6%	95.3%	100%	84.5%	0%	13.7%	31.9%	1.6%
2	68.7%	19.7%	95.4%	100%	84.7%	0%	13.7%	32.1%	1.7%
3	68.4%	19.6%	95.7%	100%	85.1%	0%	13.7%	32.1%	1.8%
4	68.5%	19.6%	95.6%	100%	84.9%	0%	13.3%	32.2%	1.7%
5	68.4%	19.6%	95.7%	100%	85.0%	0%	13.6%	32.2%	1.6%
mean	68.5%	19.6%	95.5%	100%	84.8%	0%	13.6%	32.1%	1.7%
std	0.1%	0.0%	0.2%	0.0%	0.2%	0.0%	0.2%	0.1%	0.1%

Supplementary Table 12: **Population fractions of helices estimated by SLEQ for the 126-235 region of the *E. coli* 16S ribosomal RNA based on 5 independent simulated read datasets drawn from the real reactivity profile.**

	BP Removed	ON+:OFF+:OTHER
Control		39% : 61% : 0%
	ON+	
PT	{A49-U66}	32% : 68% : 0%
	{A49-U66, A55-U60}	26% : 74% : 0%
	{A49-U66, A55-U60, C50-G65}	22% : 78% : 0%
	{A49-U66, A55-U60, C50-G65, U54-A61}	21% : 79% : 0%
	{A49-U66, A55-U60, C50-G65, U54-A61, C51-G64}	14% : 86% : 0%
	{A49-U66, A55-U60, C50-G65, U54-A61, C51-G64, U53-C62}	12% : 88% : 0%
	{A49-U66, A55-U60, C50-G65, U54-A61, C51-G64, U53-C62, A52-U63}	10% : 90% : 0%
LRs	{A10-U38}	28% : 72% : 0%
	{A40-U48}	36% : 64% : 0%
	{A10-U38, A40-U48}	24% : 76% : 0%
PK1	{G12-C47}	28% : 72% : 0%
	{G12-C47, U17-G42}	18% : 82% : 0%
	{G12-C47, U17-G42, G13-C46}	10% : 90% : 0%
	{G12-C47, U17-G42, G13-C46, U16-A43}	2% : 98% : 0%
	{G12-C47, U17-G42, G13-C46, U16-A43, A14-U45}	0% : 100% : 0%
	{G12-C47, U17-G42, G13-C46, U16-A43, A14-U45, G15-U44}	0% : 100% : 0%
	OFF+	
P1	{G6-C21}	49% : 51% : 0%
	{G6-C21, A10-U17}	59% : 41% : 0%
	{G6-C21, A10-U17, G7-C20}	64% : 24% : 12%
	{G6-C21, A10-U17, G7-C20, G9-C18}	67% : 7% : 26%
	{G6-C21, A10-U17, G7-C20, G9-C18, C8-G19 }	68% : 0% : 32%
P3	{G28-C37}	49% : 51% : 0%
	{G28-C37, U30-A35}	57% : 43% : 0%
	{G28-C37, U30-A35, C29-G36}	64% : 28% : 8%

Supplementary Table 13: **SLEQ's performance given the perturbation of important structures in the candidate set for the *crcB* cotranscriptional data.** The first column lists the disturbed motif. The second column lists the removed base pairs and the third column lists the redistribution of population fractions of ON+, OFF+ and OTHER clusters.

		Minimal PCC	
ASD	16S	*add*(without)	*add*(with)
10^5	1.0	1.0	1.0
10^4	1.0	1.0	1.0
10^3	0.92	0.97	0.96
10^2	0.43	0.85	0.84
10	0.16	0.37	0.03

Supplementary Table 14: **Exploration of SLEQ's reproducibility at various average sequencing depths (ASD) for the *E. coli* 16S ribosomal RNA and *add* RNA in the absence and presence of ligand.** For each study case at each ASD level, we generated 10 independent simulated libraries and calculated the Pearson correlation coefficient (PCC) between each pair of libraries. To measure the reproducibility of SLEQ's reconstruction over 10 libraries, we examined the minimum PCC. We observed that the minimum PCC was greater than 0.92 for all cases with ASD $>= 10^3$.

Read Numbers	ASD	Standard deviation Mutation	Truncation
10^7	$3 * 10^5$	0	0
10^6	$3 * 10^4$	0	0
10^5	$3 * 10^3$	1.7%	0.5%
10^4	$3 * 10^2$	3.9%	1%
10^3	30	4.1%	3.5%
800	24	5.8%	3.7%
500	15	4.7%	4.8%
400	12	6.7%	5.4%
300	9	8.8%	5.5%
200	6	13.4%	7.2%
100	3	13.7%	9.1%

Supplementary Table 15: **Comparison of mutation and truncation modes with the human *MRPS21* riboSNitch at various average sequencing depths.** We downsampled reads from 10^7 to 100 for both modes so that the ASD ranged from $3 * 10^5$ to 3. For each ASD, this down sampling process was repeated 10 times to generate 10 independent libraries. For both modes and each ASD, we calculated standard deviations of estimated population fractions over 10 libraries. We did not observe any significant difference between results for the two modes (p-value=0.35).

Transcript length	Runs	Without ligand ON+:OFF+:OTHER	With ligand ON+:OFF+:OTHER
78nt	1	0% : 100% : 0%	51% : 49% : 0%
	2	0% : 84% : 16%	55% : 45% : 0%
	3	0% : 97% : 3%	53% : 47% : 0%
	4	0% : 100% : 0%	55% : 45% : 0%
	5	0% : 100% : 0%	52% : 48% : 0%
	6	0% : 100% : 0%	55% : 45% : 0%
	7	0% : 100% : 0%	55% : 45% : 0%
	8	0% : 100% : 0%	54% : 46% : 0%
	9	0% : 100% : 0%	56% : 44% : 0%
	10	0% : 100% : 0%	53% : 47% : 0%
	mean	0% : 98% : 2%	54% : 46% : 0%
	std	0% : 4.8% : 4.8%	1.5% : 1.5% : 0%
75nt	1	0% : 89% : 11%	41% : 59% : 0%
	2	0% : 100% : 0%	44% : 56% : 0%
	3	0% : 100% : 0%	41% : 59% : 0%
	4	0% : 100% : 0%	39% : 61% : 0%
	5	0% : 100% : 0%	41% : 59% : 0%
	6	0% : 100% : 0%	35% : 65% : 0%
	7	0% : 100% : 0%	43% : 57% : 0%
	8	0% : 100% : 0%	41% : 59% : 0%
	9	0% : 100% : 0%	42% : 58% : 0%
	10	0% : 100% : 0%	41% : 59% : 0%
	mean	0% : 99% : 1%	41% : 59% : 0%
	std	0% : 3.3% : 3.3%	2.3% : 2.3% : 0%
69nt	1	21% : 79% : 0%	61% : 39% : 0%
	2	22% : 78% : 0%	61% : 39% : 0%
	3	20% : 76% : 4%	61% : 39% : 0%
	4	15% : 85% : 0%	51% : 49% : 0%
	5	18% : 82% : 0%	57% : 43% : 0%
	6	14% : 75% : 11%	61% : 39% : 0%
	7	17% : 83% : 0%	56% : 44% : 0%
	8	14% : 73% : 13%	58% : 42% : 0%
	9	17% : 83% : 0%	58% : 42% : 0%
	10	21% : 79% : 0%	61% : 39% : 0%
	mean	18% : 79% : 3%	58% : 42% : 0%
	std	2.8% : 3.7% : 4.8%	3.1% : 3.1% : 0%

Supplementary Table 16: **Verification of SLEQ's robust reconstruction on 10 different Boltzmann ensembles for 3 different transcript lengths without and with ligand.**

Transcript length	Runs	Without ligand ON+:OFF+:OTHER	With ligand ON+:OFF+:OTHER
66nt	1	27% : 68% : 5%	62% : 38% : 0%
	2	34% : 66% : 0%	65% : 35% : 0%
	3	20% : 62% : 18%	65% : 35% : 0%
	4	35% : 65% : 0%	63% : 37% : 0%
	5	34% : 66% : 0%	59% : 41% : 0%
	6	29% : 56% : 15%	65% : 35% : 0%
	7	25% : 62% : 13%	63% : 37% : 0%
	8	26% : 68% : 6%	58% : 42% : 0%
	9	29% : 62% : 9%	57% : 43% : 0%
	10	30% : 66% : 4%	59% : 41% : 0%
	mean	29% : 64% : 7%	62% : 38% : 0%
	std	4.4% : 3.5% : 6.2%	2.9% : 2.9% : 0%
57nt	1	27% : 0% : 73%	46% : 0% : 54%
	2	23% : 0% : 77%	46% : 0% : 54%
	3	21% : 0% : 79%	50% : 0% : 50%
	4	20% : 0% : 80%	56% : 0% : 44%
	5	34% : 0% : 66%	59% : 0% : 41%
	6	28% : 0% : 72%	49% : 0% : 51%
	7	13% : 0% : 87%	46% : 0% : 54%
	8	14% : 0% : 86%	44% : 0% : 56%
	9	39% : 0% : 61%	62% : 0% : 38%
	10	21% : 0% : 79%	44% : 0% : 56%
	mean	24% : 0% : 76%	50% : 0% : 50%
	std	7.8% : 0% : 7.8%	6.2% : 0% : 6.2%

Supplementary Table 17: **Verification of SLEQ's robust reconstruction on 10 different Boltzmann ensembles for 2 different transcript lengths without and with ligand.**

	Truncation		Mutation	
η	$\hat{\eta}$	$\hat{\rho}_A : \hat{\rho}_B$	$\hat{\eta}$	$\hat{\rho}_A : \hat{\rho}_B$
0.010	0.010	0.197 : 0.803	0.010	0.198 : 0.802
0.020	0.020	0.198 : 0.802	0.018	0.197 : 0.803
0.030	0.030	0.196 : 0.804	0.029	0.199 : 0.801
0.040	0.040	0.198 : 0.802	0.037	0.197 : 0.803
0.050	0.050	0.200 : 0.800	0.047	0.194 : 0.806
0.060	0.060	0.198 : 0.802	0.059	0.200 : 0.800
0.070	0.070	0.199 : 0.801	0.065	0.199 : 0.801
0.080	0.080	0.200 : 0.800	0.078	0.200 : 0.800
0.090	0.090	0.200 : 0.800	0.085	0.199 : 0.801
0.100	0.100	0.196 : 0.804	0.095	0.196 : 0.804

Supplementary Table 18: **Performance evaluation with the preQ$_1$ RNA at different η values.** The left-most column shows the ground truth value of η, which is the probability of modification at an unpaired nucleotide. The second and fourth columns show SLEQ's estimates of η in both modes. The third and fifth columns show respective estimated relative abundances of the two competing structures shown in **Supplementary Figure 29**. The ground truth in all simulation was kept fixed at $0.2 : 0.8$ for preQ$_1$_A and preQ$_1$_B, respectively.

	Truncation		Mutation	
$\rho_A : \rho_B$	$\hat{\eta}$	$\hat{\rho}_A : \hat{\rho}_B$	$\hat{\eta}$	$\hat{\rho}_A : \hat{\rho}_B$
0.05 : 0.95	0.05	0.049 : 0.951	0.047	0.047 : 0.953
0.1 : 0.9	0.05	0.097 : 0.903	0.047	0.1 : 0.9
0.2 : 0.8	0.05	0.198 : 0.802	0.047	0.198 : 0.802
0.3 : 0.7	0.05	0.3 : 0.7	0.047	0.3 : 0.7
0.4 : 0.6	0.05	0.4 : 0.6	0.047	0.4 : 0.6
0.5 : 0.5	0.05	0.5 : 0.5	0.047	0.5 : 0.5
0.6 : 0.4	0.05	0.6 : 0.4	0.047	0.6 : 0.4
0.7 : 0.3	0.05	0.7 : 0.3	0.047	0.7 : 0.3
0.8 : 0.2	0.05	0.8 : 0.2	0.048	0.8 : 0.2
0.9 : 0.1	0.05	0.9 : 0.1	0.049	0.9 : 0.1
0.95 : 0.05	0.05	0.95 : 0.05	0.048	0.95 : 0.05

Supplementary Table 19: **Performance evaluation with the preQ$_1$ RNA at different ensemble compositions.** The left-most column shows ground truth relative abundances of structures preQ$_1$_A and preQ$_1$_B shown in **Supplementary Figure 29** (ρ_A and ρ_B). The third and fifth columns show their respective estimated relative abundances per mode. The second and fourth columns show corresponding estimates of η. The ground truth of η in all simulation was kept fixed at 0.05.

Truncation $\hat{\rho}_A : \hat{\rho}_B$	*Mutation* $\hat{\rho}_A : \hat{\rho}_B$
0.21 : 0.76	0.21 : 0.75
0.20 : 0.71	0.20 : 0.73
0.18 : 0.73	0.20 : 0.75
0.20 : 0.75	0.21 : 0.72
0.24 : 0.75	0.20 : 0.74
0.20 : 0.74	0.22 : 0.77
0.22 : 0.78	0.21 : 0.73
0.17 : 0.77	0.19 : 0.74
0.18 : 0.74	0.20 : 0.73
0.20 : 0.74	0.21 : 0.72

Supplementary Table 20: **Performance evaluation with the preQ$_1$ RNA in the presence of noisy ensemble composition.** Relative abundances of structures preQ$_1$_A and preQ$_1$_B shown in **Supplementary Figure 29** (ρ_A and ρ_B) were kept fixed at 0.2 : 0.7. Besides two dominant structures, we randomly selected other 10 structures from candidate set as minor structures. We assigned random values as the relative abundances of these 10 minor structures and normalized them to 0.1. We tested 10 times based on 10 independent sets of minor structures. Two columns show the estimated relative abundances of two dominant structures in both modes for 10 tests. We observed that SLEQ is capable to predict dominant structures from noisy ensemble composition.

SNR	Ground truth $\rho_A : \rho_B$	Mutation $\hat{\rho}_A : \hat{\rho}_B$	Truncation $\hat{\rho}_A : \hat{\rho}_B$
$40dB$	0.2 : 0.8	0.199 : 0.801	0.199 : 0.801
$30dB$	0.2 : 0.8	0.197 : 0.803	0.197 : 0.803
$20dB$	0.2 : 0.8	0.196 : 0.804	0.192 : 0.808

Supplementary Table 21: **Performance evaluation with the preQ$_1$ RNA at various noise levels in SP data.** Relative abundances of structures preQ$_1$_A and preQ$_1$_B are shown in **Supplementary Figure 29** (ρ_A and ρ_B) were kept fixed at $0.2 : 0.8$. We define SNR $= \mu/\sigma$, where μ is simulated (noiseless) SP data, and σ is standard deviation of noise that we varied in simulations. For different SNRs (20dB, 30dB, 40dB), we calculated varying σs, and then different levels of Gaussian noises $\tilde{}N(0, \sigma)$ were generated and added to simulated SP data. The left-most column is the values of SNR. The second column is the ground truth of relative abundances. The third and forth columns show respective estimated relative abundances per mode. When SNR $> 10dB$, our method accurately recovered the ground truth.

Run	Ground truth η	$\rho_A : \rho_B$	Mutation $\hat{\eta}$	$\hat{\rho}_A : \hat{\rho}_B$	Truncation $\hat{\eta}$	$\hat{\rho}_A : \hat{\rho}_B$
1	0.05	0.2 : 0.8	0.05	0.21 : 0.78	0.056	0.00 : 0.60
2	0.05	0.2 : 0.8	0.05	0.18 : 0.75	0.053	0.03 : 0.51
3	0.05	0.2 : 0.8	0.05	0.17 : 0.83	0.06	0.07 : 0.48
4	0.05	0.2 : 0.8	0.053	0.15 : 0.84	0.059	0.07 : 0.60
5	0.05	0.2 : 0.8	0.052	0.07 : 0.93	0.061	0.05 : 0.68
6	0.05	0.2 : 0.8	0.053	0.19 : 0.81	0.07	0.02 : 0.45
7	0.05	0.2 : 0.8	0.049	0.17 : 0.68	0.056	0.05 : 0.43
8	0.05	0.2 : 0.8	0.05	0.14 : 0.84	0.057	0.00 : 0.45
9	0.05	0.2 : 0.8	0.053	0.1 : 0.9	0.06	0.04 : 0.53
10	0.05	0.2 : 0.8	0.0476	0.17 : 0.79	0.057	0.04 : 0.77

Supplementary Table 22: **Performance evaluation with the preQ$_1$ RNA at SNR = $10dB$.** Relative abundances of structures preQ$_1$_A and preQ$_1$_B shown in **Supplementary Figure 29** (ρ_A and ρ_B) were kept fixed at 0.2 : 0.8. The way of disturbing SP data with additive Gaussian noises was as described in **Supplementary Table 21**. We ran 10 times for 10 independent noisy SP data. The left-most column is the indexes of 10 runs. The second and third columns show the ground truth of η and relative abundances. The forth and sixth columns show η estimates in both modes. The fifth and seventh columns show respective estimated relative abundances per mode. When SNR = $10dB$, abundance estimates are poor due to large amount of noise, especially in truncation mode.

Run	Ground truth $\rho_A : \rho_B$	Mutation $\hat{\rho}_A : \hat{\rho}_B$	Truncation $\hat{\rho}_A : \hat{\rho}_B$
1	$0.2 : 0.8$	$0.11 : 0.89$	$0.05 : 0.93$
2	$0.2 : 0.8$	$0.17 : 0.61$	$0.16 : 0.84$
3	$0.2 : 0.8$	$0.21 : 0.79$	$0.10 : 0.65$
4	$0.2 : 0.8$	$0.16 : 0.59$	$0.10 : 0.90$
5	$0.2 : 0.8$	$0.16 : 0.83$	$0.09 : 0.79$
6	$0.2 : 0.8$	$0.12 : 0.81$	$0.16 : 0.83$
7	$0.2 : 0.8$	$0.22 : 0.49$	$0.19 : 0.80$
8	$0.2 : 0.8$	$0.15 : 0.85$	$0.09 : 0.79$
9	$0.2 : 0.8$	$0.11 : 0.75$	$0.09 : 0.97$
10	$0.2 : 0.8$	$0.13 : 0.87$	$0.04 : 0.74$

Supplementary Table 23: **Performance evaluation with the preQ$_1$ RNA at SNR $= 10dB$ when $\hat{\eta}$s of two modes were kept fixed at 0.05.** The way of disturbing SP data with additive Gaussian noises was the same as **Supplementary Table 21**. We ran 10 times for 10 independent noisy SP data. The left-most column is the indexes of 10 runs.The second column is the ground truth of relative abundances. The third and forth columns show respective estimated relative abundances per mode. When SNR $= 10dB$ and $\hat{\eta}$ was fixed to the ground truth, the results of abundance estimates in truncation mode became better compared to **Supplementary Table 22**.

	Truncation		Mutation	
η	$\hat{\eta}$	$\hat{\rho}_A : \hat{\rho}_B : \hat{\rho}_C : \hat{\rho}_D$	$\hat{\eta}$	$\hat{\rho}_A : \hat{\rho}_B : \hat{\rho}_C : \hat{\rho}_D$
0.010	0.010	0.13 : 0.2 : 0.25 : 0.42	0.009	0.14 : 0.2 : 0.25 : 0.41
0.020	0.020	0.15 : 0.2 : 0.25 : 0.40	0.018	0.15 : 0.2 : 0.25 : 0.40
0.030	0.030	0.15 : 0.2 : 0.25 : 0.40	0.028	0.14 : 0.2 : 0.25 : 0.41
0.040	0.040	0.15 : 0.2 : 0.25 : 0.40	0.039	0.14 : 0.2 : 0.25 : 0.41
0.050	0.050	0.15 : 0.2 : 0.25 : 0.40	0.046	0.15 : 0.2 : 0.25 : 0.40

Supplementary Table 24: **Performance evaluation with the VcQrr$_3$ RNA at different η values.** The left-most column shows the ground truth value of η, which is the probability of modification at an unpaired nucleotide. The second and fourth columns show SLEQ's estimates of η in both modes. The third and fifth columns show respective estimated relative abundances of the four alternative structures shown in **Supplementary Figure 31**. The ground truth in all simulation was kept fixed at 0.15 : 0.2 : 0.25 : 0.40 for four structures, respectively.

		Truncation		Mutation
ρ	$\hat{\eta}$	$\hat{\rho}_A : \hat{\rho}_B : \hat{\rho}_C : \hat{\rho}_D$	$\hat{\eta}$	$\hat{\rho}_A : \hat{\rho}_B : \hat{\rho}_C : \hat{\rho}_D$
0.15 : 0.4 : 0.2 : 0.25	0.03	0.15 : 0.4 : 0.2 : 0.25	0.028	0.14 : 0.41 : 0.2 : 0.25
0.1 : 0.5 : 0.3 : 0.1	0.03	0.09 : 0.3 : 0.1 : 0.51	0.028	0.09 : 0.3 : 0.1 : 0.51

Supplementary Table 25: **Performance evaluation with the VcQrr$_3$ RNA at two different ensemble compositions.** The left-most column shows ground truth relative abundances of four alternative structures shown in **Supplementary Figure 31**. The third and fifth columns show their respective estimated relative abundances per mode. The second and fourth columns show corresponding estimates of η. The ground truth of η in all simulation was kept fixed at 0.03.

Ground truth	Truncation	Mutation
$\rho_A : \rho_B : \rho_C : \rho_D$	$\hat{\rho}_A : \hat{\rho}_B : \hat{\rho}_C : \hat{\rho}_D$	$\hat{\rho}_A : \hat{\rho}_B : \hat{\rho}_C : \hat{\rho}_D$
0.15 : 0.4 : 0.2 : 0.15	0.15 : 0.45 : 0.23 : 0.15	0.15 : 0.44 : 0.22 : 0.16

Supplementary Table 26: **Performance evaluation with the VcQrr$_3$ RNA in the presence of noisy ensemble composition.** In addition to the four dominant structures, we randomly selected 10 structures from the candidate set as minor structures. We assigned random values as the relative abundances of these 10 minor structures and normalized them to 0.1. The left-most column is the ground truth of relative abundances. The second and third columns show the estimated relative abundances of four dominant structures in both modes. We observed that SLEQ is capable to predict dominant structures from noisy ensemble composition.

	Ground truth	Mutation	Truncation
SNR	$\rho_A : \rho_B : \rho_C : \rho_D$	$\hat{\rho}_A : \hat{\rho}_B : \hat{\rho}_C : \hat{\rho}_D$	$\hat{\rho}_A : \hat{\rho}_B : \hat{\rho}_C : \hat{\rho}_D$
$40dB$	$0.15 : 0.4 : 0.25 : 0.2$	$0.15 : 0.40 : 0.25 : 0.20$	$0.13 : 0.41 : 0.26 : 0.20$
$30dB$	$0.15 : 0.4 : 0.25 : 0.2$	$0.14 : 0.40 : 0.25 : 0.20$	$0.15 : 0.40 : 0.25 : 0.20$
$20dB$	$0.15 : 0.4 : 0.25 : 0.2$	$0.12 : 0.42 : 0.25 : 0.21$	$0.12 : 0.42 : 0.25 : 0.18$
$10dB$	$0.15 : 0.4 : 0.25 : 0.2$	$0.09 : 0.42 : 0.14 : 0.11$	$0.00 : 0.29 : 0.00 : 0.00$

Supplementary Table 27: **Performance evaluation with the VcQrr$_3$ RNA at various noise levels in SP data.** Relative abundances of four alternative structures shown in **Supplementary Figure 31** were kept fixed at $0.15 : 0.4 : 0.25 : 0.2$. The way of disturbing SP data with additive Gaussian noises was the same as **Supplementary Table 21**. The left-most column is the values of SNR. The second column is the ground truth of relative abundances. The third and forth columns show respective estimated relative abundances per mode. When SNR $> 10dB$, our method accurately recovered the ground truth.

SNR	Ground truth<br>$\rho_A : \rho_B : \rho_C$	Mutation<br>$\hat{\rho}_A : \hat{\rho}_B : \hat{\rho}_C$	Truncation<br>$\hat{\rho}_A : \hat{\rho}_B : \hat{\rho}_C$
$40dB$	$0.5 : 0.3 : 0.2$	$0.501 : 0.299 : 0.2$	$0.502 : 0.303 : 0.195$
$30dB$	$0.5 : 0.3 : 0.2$	$0.5 : 0.3 : 0.2$	$0.5 : 0.3 : 0.2$
$20dB$	$0.5 : 0.3 : 0.2$	$0.49 : 0.29 : 0.18$	$0.52 : 0.29 : 0.18$
$10dB$	$0.5 : 0.3 : 0.2$	$0.41 : 0.16 : 0.05$	$0.00 : 0.00 : 0.00$

Supplementary Table 28: **Performance evaluation with the MST RNA at various noise levels in SP data.** Relative abundances of three alternative structures shown in **Supplementary Figure 32** were kept fixed at $0.5 : 0.3 : 0.2$. The way of disturbing SP data with additive Gaussian noises was the same as **Supplementary Table 21**. The left-most column is the values of SNR. The second column is the ground truth of relative abundances. The third and forth columns show respective estimated relative abundances per mode. When SNR $> 10dB$, our method accurately recovered the ground truth.

SNR	Ground truth<br>$\rho_A : \rho_B : \rho_C$	Mutation<br>$\hat{\rho}_A : \hat{\rho}_B : \hat{\rho}_C$	Truncation<br>$\hat{\rho}_A : \hat{\rho}_B : \hat{\rho}_C$
$40dB$	$0.56 : 0.27 : 0.17$	$0.56 : 0.27 : 0.17$	$0.56 : 0.27 : 0.17$
$30dB$	$0.56 : 0.27 : 0.17$	$0.56 : 0.27 : 0.17$	$0.56 : 0.27 : 0.17$
$20dB$	$0.56 : 0.27 : 0.17$	$0.54 : 0.28 : 0.18$	$0.53 : 0.28 : 0.19$
$10dB$	$0.56 : 0.27 : 0.17$	$0.18 : 0.18 : 0.06$	$0.00 : 0.00 : 0.00$

Supplementary Table 29: **Performance evaluation with the TBWN RNA at various noise levels in SP data.** Relative abundances of the three alternative structures shown in **Supplementary Figure 33** were kept fixed at $0.56 : 0.27 : 0.17$. The way of disturbing SP data with additive Gaussian noises was the same as **Supplementary Table 21**. The left-most column is the values of SNR. The second column is the ground truth of relative abundances. The third and forth columns show respective estimated relative abundances per mode. When SNR $> 10dB$, our method accurately recovered the ground truth.

3 SUPPLEMENTARY METHODS

3.1 Pattern reduction

Accommodating the large number of theoretically possible mutation patterns in simulation poses a challenging computational problem. However, with real data the number of observed mutation patterns is manageable. Therefore, a pattern reduction technique is necessary to generate simulated mutation patterns. We calculated pattern probabilities in ascending order of mutation numbers per read. Each time, we obtained the probability of a pattern and drew reads according to its likelihood. If the number of reads is 0, we discarded this pattern and continued to the calculation for the next pattern. We employed a threshold for termination (e.g.: 0.98). Once the accumulated probability of all generated patterns exceeded this threshold, the pattern generation terminated.

3.2 Design matrix for base-selective profiling techniques

Beyond SHAPE, different structure profiling techniques can be base-selective. This is the case for DMS and hence analyzing DMS data with SLEQ requires minor adjustments to the design matrix. In this section, we use DMS as a representative example to describe the construction of the design matrix. While with SHAPE data, we defined four events, in this case we define six events at a finer resolution. This is to accommodate for the different modification/detection propensities. We consider the events $\mathcal{A}_A$, $\mathcal{A}_C$, $\mathcal{B}_A$, and $\mathcal{B}_C$ as the A/C-specific counterparts of the above-defined events $\mathcal{A}$ and $\mathcal{B}$ (see Online Methods), with the following probabilities:

- $Pr(\mathcal{A}_A) = 1 - (1 - \eta_A)(1 - \gamma_l)$.
- $Pr(\mathcal{B}_A) = (1 - \eta_A)(1 - \gamma_l)$.
- $Pr(\mathcal{A}_C) = 1 - (1 - \eta_C)(1 - \gamma_l)$.
- $Pr(\mathcal{B}_C) = (1 - \eta_C)(1 - \gamma_l)$.
- $Pr(\mathcal{C}_{extend}) = \gamma_l$.
- $Pr(\mathcal{D}_{extend}) = 1 - \gamma_l$.

Additionally, the above-defined events $\mathcal{C}$, $\mathcal{D}$ (see Online Methods) are extended to include unpaired U and G sites. We denote the set of unpaired A sites in the first $p-1$ sites in structure s as UA_{ps}. Similarly, we have UC_{ps} for site C. We also denote the set of other sites in the first $p-1$ sites in structure s as O_{ps}. Thus, x_{ps} in truncation mode is calculated as follows: If site p is an unpaired A site in structure s,

$$x_{ps} = \Pi_{l \in UA_{ps}}(1-\eta_A)(1-\gamma_l)\Pi_{l \in UC_{ps}}(1-\eta_C)(1-\gamma_l)\Pi_{l \in O_{PS}}(1-\gamma_l)[1-(1-\eta_A)(1-\gamma_p)]. \quad (1)$$

If site p is an unpaired C site in structure s,

$$x_{ps} = \Pi_{l \in UA_{ps}}(1-\eta_A)(1-\gamma_l)\Pi_{l \in UC_{ps}}(1-\eta_C)(1-\gamma_l)\Pi_{l \in O_{PS}}(1-\gamma_l)[1-(1-\eta_C)(1-\gamma_p)]. \quad (2)$$

If site p is a paired site or U/G site in structure s,

$$x_{ps} = \Pi_{l \in UA_{ps}}(1-\eta_A)(1-\gamma_l)\Pi_{l \in UC_{ps}}(1-\eta_C)(1-\gamma_l)\Pi_{l \in O_{PS}}(1-\gamma_l)\gamma_p. \quad (3)$$

For the complete read pattern $L+1$,

$$x_{L+1,s} = \Pi_{l \in UA_{L+1,s}}(1-\eta_A)(1-\gamma_l)\Pi_{l \in UC_{L+1,s}}(1-\eta_C)(1-\gamma_l)\Pi_{l \in O_{L+1,fs}(1-\gamma_l)}. \quad (4)$$

Similarly, in mutation mode, we extends SHAPE's events (see Online Methods) to accommodate DMS as follows:

- $Pr(\mathcal{A}_A) = 1-(1-\eta_A)(1-\gamma_l)$.
- $Pr(\mathcal{B}_A) = (1-\eta_A)(1-\gamma_l)$.
- $Pr(\mathcal{A}_C) = 1-(1-\eta_C)(1-\gamma_l)$.
- $Pr(\mathcal{B}_C) = (1-\eta_C)(1-\gamma_l)$.
- $Pr(\mathcal{C}_{extend}) = \gamma_l$.
- $Pr(\mathcal{D}_{extend}) = 1-\gamma_l$.

Given a pattern p and structure s, we denote the set of unpaired and non-mutated/mutated A sites as UNA_{ps} and UMA_{ps}, respectively. Similarly, we have UNC_{ps} and UMC_{ps} for C sites. The remaining set of non-mutated sites (U/G and paired A/C) is denoted as ON_{ps} and that of mutated ones as OM_{ps}. We can now express x_{ps} as follows:

$$\begin{aligned} &x_{ps} = \Pi_{l \in UMA_{ps}}[1-(1-\eta_A)(1-\gamma_l)]\Pi_{l \in UNA_{ps}}(1-\eta_A)(1-\gamma_l)\Pi_{l \in UMC_{ps}}[1-(1-\eta_C) \\ &(1-\gamma_l)]\Pi_{l \in UNC_{ps}}(1-\eta_C)(1-\gamma_l)\Pi_{l \in OM_{ps}}\gamma_l\Pi_{l \in ON_{ps}}(1-\gamma_l) \end{aligned} \tag{5}$$

The estimation of β_l and γ_l in the DMS case is the same as in the SHAPE case. Since η_A and η_C are different in DMS due to the base-selective feature, their estimations are adjusted. The first step remains unchanged: order all βs and calculate their mean. Then, select all βs which are from A sites and are larger than the mean. η_A is set to the median of these selected βs. η_C can be obtained similarly.

3.3 Analysis of human *MRPS21* riboSNitch from DMS-MaPseq data

Raw DMS-MaPseq reads were quality-trimmed and stripped of sequencing adapters using cutadapt[13] and then were aligned using bowtie2 ver. 2.2.5[14]. The total number of raw reads was 102466345, of which 92135108 were successfully aligned. Of the aligned reads, 62135800 were aligned to allele A and 29999308 were aligned to allele C. The mutation of U and G nucleotides were assumed to originate from background noise and were ignored. We further discarded reads that did not have information about all 33 nt of interest. We were left with 38485845 reads for allele A and 18264689 for allele C. The ground truth of the relative abundances of these two alleles as assessed from the final alignment result was 0.678 : 0.322 (note that this is similar to what may be assessed without discarding any of the aligned reads). **Supplementary Table 6** summarizes the distribution of reads with respect to the number of mutations per read.

To run SLEQ on this data, we require two inputs. The first input was obtained by binning aligned reads into modification patterns. There were 614 and 34 possible patterns for mutation and truncation modes respectively. For the second input, we started by generating a structure candidate set of 1000 samples for each allele. Many structures in each candidate set were identical in pairing states

for the 33 nts of interest. We thus randomly selected a representative for such identical structures and discarded the remaining structures. Thus, the size of the candidate sets was reduced to 92 and 22 for alleles A and C, respectively.

As there were no A and C sites simultaneously unpaired for both alleles, SLEQ was unable to accurately estimate η_A and η_C. However, it is still able to perform its core function of reconstructing the structure landscape which we sought to validate. We first estimate $\boldsymbol{\beta}$ and decompose it into η_A and η_C using the ground truth for relative abundances. For example, if an adenine site s was unpaired in allele A but not in allele C, we obtained an estimate for η_A as $\beta_s/0.678$ and if an adenine site s was paired in allele A but not int allele C, we obtained an estimate for η_A as $\beta_s/0.322$. A similar approach was taken to obtain an η_C estimate. Then, we averaged all estimates for unpaired adenine and cytosine separately and fed them to SLEQ. Using this approach, we obtained $\eta_A = 0.13, \eta_C = 0.05$ in mutation mode and $\eta_A = 0.14, \eta_C = 0.05$ in truncation mode, which is in agreement with different reaction affinities of adenine and cytosine. We ran SLEQ 10 times with 10 independent sets of structure candidates. **Supplementary Table 1** summarizes the results from these runs, including the number of candidate structures, the estimated the relative abundances of the two alleles and the number of selected structures in both modes.

3.4 Analysis of *crcB* fluoride riboswitch from SHAPE-Seq data

To explore the formation of the P1 and P3 hairpins, we processed transcripts of lengths 26 nt and 41 nt respectively. For the transcript of length 26 nt, 1000 candidate structures were generated using ViennaRNA. There were originally 20 unique candidate structures after removing duplicates. After prefiltering, the candidate set was further reduced to 19 and 13 structures in the absence and presence of the ligand respectively. All structures consistently include the P1 hairpin, as the sequence was too short to form other motifs. For the transcript of length 41 nt, the number of unique candidate structures was 53, which was reduced to 52 and 51 in the absence and presence of ligand after prefiltering. The estimated relative abundances of the P3 hairpins by SLEQ were 61% and 68% in the absence and presence of ligand respectively. These results suggest that the formation of the P1 and P3 hairpin were ligand-independent. Ligand-dependent dynamics come into play with increasing transcript length. To explore these ligand-dependent dynamics, we analyzed transcripts

of lengths between $55 - 80$ nt. For length in the range of $55 - 61$ nt, we 'spiked-in' three structures into the candidate set: the first contained PK1 only, the second contained PK1 and LR1, the third contained PK1 and 2 LR (see **Supplementary Fig. 5a, b, c**). As the terminator started to form at ~ 62 nt, for transcripts of length $62 - 80$ nt, we 'spiked-in' three additional structures: the first contained PK1 and PT, the second contained PK1, LR1 and PT, the third contained PK1, 2 LRs and PT (**Supplementary Fig. 5d, e, f**). As SLEQ only considered pairing states (unconstrained/constrained) of nucleotides in a structure, it treated the pseudoknot PK1 and LRs as constrained nucleotides like other paired nucleotides. SLEQ's outputs from the analysis of all these transcripts are summarized in **Supplementary Table 2**.

Furthermore, we compared results before and after 'spiking-in' structures with one LR1. After this 'spiking-in', we observed that SLEQ selected the newly added structure with LR1 as well as structures with two LRs and rarely selected the structure without LRs. This implies that SLEQ is able to distinguish the difference between structures with and without LRs. A recent NMR study of the same fluoride riboswith showed that the structure with LR1 was important to ligand-binding bifurcation. This structure was identified as an excited state (ES)[15]. The ligand stabilized LR2 to prevent transition to the ES and thereby prevented the formation of OFF state. In our analysis, SLEQ only detected ES at a few transcript lengths. This is because the ES was short-lived and hardly detected by SHAPE-Seq due to resolution limitations[15]. Another important finding was that the population fractions of ON+, OFF+ and OTHER were nearly identical and undisturbed by the newly selected ES structure (see **Supplementary Fig. 10).** The only discrepancy was in the redistribution of the internal composition of ON+ structures at a few transcript lengths in which ES was detected. This finding suggests that SLEQ is robust to minor perturbations in candidate structures and our analysis of population fractions via manual clustering of SLEQ's outputs is reasonable.

3.5 Analysis of *crcB* fluoride riboswitch mutants from SHAPE-Seq data

To corroborate our interpretation of structural transitions in fluoride riboswitches, we analyzed mutants M18 (G13A A14U), M19 (U45A C46U), M20 (G69A A70U), M21 (M18 and M19), M22 (M19 and M20) and M23(M18, M19 and M20) in the presence of the ligand (see **Supplementary**

Fig. 8a).

M18 was located in PK1 and expected to affect PK1 formation. From the PPP of M18 (see **Supplementary Fig. 8b**), we observed the P1, P3 hairpins and complete terminator stems. The pseudoknot PK1 was absent, which validated our assumption. M19 was located in both PK1 and the CT. **Supplementary Figure 8c** shows that the CT was affected on several base pairs while PK1 was disrupted completely. M20 was located only in the CT. From its PPP (see **Supplementary Fig. 8d**), we observed the P1, P3 stems, PK1, LR and the affected terminator. The relative abundance of PK1 was 43%. M21 consisted of M18 and M19. We observed the P1, P3 stems, PK1, LR and the affected terminator in the PPP (see **Supplementary Fig. 8e**). The relative abundance of PK1 was 18%. As expected, PK1 was restored and the CT was disrupted. M22 involved M19 and M20. Its PPP (see **Supplementary Fig. 8f**) shows that the P1, P3 stems and the CT were predicted. As expected, PK1 was disrupted completely, but CT was unaffected. M23 included M18, M19 and M20, which should restore both PK1 and the terminator. **Supplementary Figure 8g** shows the P1, P3 stems, PK1, LR and the CT. The relative abundance of PK1 was 29%. We found that PK1 was more easily disrupted: it disappeared completely due to relevant mutations while the CT was affected only on several base pairs and did not vanish completely.

3.6 Analysis of *crcB* fluoride riboswitch in equilibrium from SHAPE-Seq data

We analyzed the equilibrium SHAPE-Seq data for the *crcB* fluoride riboswitch, published in [16], and compared the results with the cotranscriptional case. The inspected transcript length was 77 nt. **Supplementary Figure 11** shows the PPP of the equilibrium **(a)** and cotranscriptional **(b)** states in the absence and presence of ligand. In the equilibrium case, SLEQ predicted that only the OFF structure was dominant with and without the ligand. In contrast, for the cotranscriptional case, PK1, LR showed up in the presence of fluoride and vanished in the absence of fluoride. These results suggest that cotranscriptional folding is necessary to achieve alternative ligand-dependent structures[16].

3.7 Analysis of *thiM* TPP and *add* adenine riboswitch aptamer domains from SHAPE-Seq data

With human riboSNitch alleles and the fluoride riboswitch, we demonstrated that SLEQ can accurately reconstruct the RNA structural landscape when multiple structures coexist. In this section, we demonstrate the applicability of SLEQ to RNAs with only one dominant structure. For this purpose, we used *thiM* TPP (TPP) and *add* adenine riboswitch aptamer domain (*add* aptamer) data in the presence of ligand (published in [17]). We compared SLEQ's results with the ligand-bound secondary structures of TPP published in [3] (**Supplementary Fig. 12b**) and *add* aptamer published in [4] (**Supplementary Fig. 13b**). **Supplementary Figure 12a** showed that the results predicted by SLEQ (below the diagonal) were consistent with ground truth (above the diagonal). The average relative abundances of P1 and P2 were 93.6% and 86.9%, respectively. The average relative abundance of P3 was 85.0%, where base pairs (G12-C29), (U16-A26), (U17-A25), (C18-G24) were 100% and the other two base pairs (C13-G28) and (C14-G27) were 61.7% and 48.6%, respectively. The relative abundances of P4 and P5 were both 100%. **Supplementary Figure 13a** shows the same consistency of SLEQ's prediction for the *add* aptamer. The average relative abundance of the P1, P2 and P3 stems were 96.3%, 66.6% and 74.2%, respectively. We observed that there were a few other structures that showed up in SLEQ's prediction. These may be due to the coexistence of minor structures with very low relative abundances in the solution or alternatively can be accounted for by the noise in the data.

3.8 Comparison with RING-MaP

To compare SLEQ to RING-MaP, we analyzed DMS-MaPseq data for the human *MRPS21* riboSNitch. The reasons why we chose this dataset are as follows: first, it was mutation data, which was required by RING-MaP; second, it had reference structures and the ground truth values of the population fractions could be reconstructed from the mutation reads. Such data with available reference structures and respective relative abundances are rare.

We applied the clustering-analysis module in the RING-MaP software to process the data. Results are shown in **Supplementary Figure 14**. The algorithm identified two clusters with popula-

tion fractions of 76% and 24% and separated respective reactivity profiles from the mixed data. We visually inspected each of the clustered reactivity profiles and compared them with reference structures of each the two alleles. We found that the top cluster in **Supplementary Figure 14** was consistent with allele A, as nucleotides at 8, 10 and 25 were highly reactive and they were all unpaired in allele A while paired in allele C. Similarly, the bottom cluster in **Supplementary Figure 14** was consistent with allele C, as nucleotides at 18 and 24 were highly reactive and they were both unpaired in allele C while paired in allele A. Therefore, the population fractions of alleles A:C estimated by RING-MaP were 76% : 24%, which was consistent with the ground truth, 68% : 32% (8% deviation). In comparison, SLEQ's estimation was 62% : 38%, which was consistent with ground truth (6% deviation) (see **Supplementary Table 7**). As for the prediction of structures, SLEQ identified structures of two alleles from the candidate set, which were consistent with references. However, RING-MaP only provided reactivity profiles for each allele. We wondered if these reactivity profiles could be used by a data-guided secondary structure prediction software to accurately predict structures for each allele. To investigate this, we used RNAstructure[5] to predict secondary structures of each allele given the respective clustered profiles by RING-MaP. The resulting structures are consistent with references and are shown in **Supplementary Figure 15**.

In conclusion, RING-MaP and SLEQ are comparable on the identification of number of conformations an RNA adopts and the estimation of respective population fractions for this data. However, two fundamental differences between the two methods are: 1) RING-MaP requires mutation data while SLEQ has no such limitation. 2) RING-MaP only provides reactivity profile of each cluster while SLEQ provides the dominant structures directly.

3.9 Comparison with M^2-REEFFIT

To compare SLEQ to M^2-REEFFIT, we analyzed SHAPE-CE data for a bistable RNA (BST), a Vibrio vulnificus adenosine deaminase (*add*) mRNA riboswitch and the 126-235 region of the *E. coli* 16S ribosomal RNA, which were published in the M^2-REEFFEIT paper[9]. The reasons why we choose these datasets are: first, we were not able to properly install the M^2-REEFFIT software to process other datasets and hence compared SLEQ's results to the reconstructions reported in

[9]; second, M^2-REEFFIT requires chemical profiling data of wild type and all single mutants, which is not always available in other datasets; third, one major advantage of choosing these data is that they have ground truth structures studied by either NMR or crystallography, which is a good reference for comparison.

3.9.1 Methodology

As all three datasets were obtained by electrophoresis-based sequencing while SLEQ operated at the NGS read level, we bridged the gap between these two data types by simulating NGS reads of wild type from corresponding CE reactivity profiles. Specifically, we downloaded corresponding CE data from RMDB[12]. To mimic the stochasticity inherent in a structure profiling experiment and in the cDNA sequencing that follows it, we replaced negative reactivity values with zeros and normalized all values by the maximum reactivity. We then multiplied the normalized reactivities by a factor α ($\alpha = 0.015$ for BST, $\alpha = 0.03$ for add and $\alpha = 0.03$ for 16S). The value of α was determined empirically to ensure the simulated reads were realistic. We took the resulting values as βs and set $\gamma = 0$. Probabilities of all read patterns were calculated according to the formula in [18, 19] and simulated truncation reads were drawn according to these probabilities. We then applied SLEQ on these simulated reads. The estimated βs calculated by SLEQ using our generated reads were the same as the real β derived from the CE data, which verified our read generation process. It is important to note that in the process of this simulation, we did not assume any ensemble compositions but instead assumed that the entire molecule population in the sample adopts a single identical average structure as characterized by the input reactivities. This ensures a fair comparison between SLEQ and M^2-REEFFIT.

3.9.2 BST

BST is a 25-nt synthetic short RNA, which adopts two competing hairpins, BST-A and BST-B. The corresponding population fractions measured by NMR were $70 \pm 5\%$ and $30 \pm 5\%$, respectively (see **Supplementary Fig. 16**)[9]. As [9] reported, M^2-REEFFIT recovered $73 \pm 11\%$ and $26 \pm 9\%$, for BST-A and BST-B respectively, using SHAPE-CE data of the wild type and all

mutants. Using SHAPE-CE data of wild type only, we ran SLEQ on 10 independent simulated datasets and corresponding results were shown in **Supplementary Table 8**. The mean and the standard deviation of population fractions calculated based on these 10 runs were BST-A:BST-B $= 71 \pm 0.2\%$ and $29 \pm 0.2\%$. Both results estimated by SLEQ and M^2-REEFFIT were generally comparable and consistent with the NMR study though SLEQ had a smaller standard deviation (see **Supplementary Table 9**).

3.9.3 *add*

For the *add* RNA, two conformations, apoA (P1,P2, P3, P4 and P5) and apoB (P1B, P2B, P3, P4B, P5), were detected by NMR in the absence of ligand while the holo structure (P1, P2, P3 and P5) became dominant in the presence of the ligand[10] (see **Supplementary Fig. 34** and Fig. 1a in [10]). The main difference between these three structures was the state of ligand-binding pocket: it was empty in apoA and constrained by interactions with the ligand in holo while completely absent in apoB. Here, the pocket refers to the circle formed by the P1, P2 and P3 helices. The difference between an empty and constrained pocket is whether or not the pocket makes contact with the ligand.

A series of NMR experiments were implemented on different conditions in [10]. To identify references from this NMR study, we looked for specific experiments with conditions similar to the chemical profiling experiments performed in Das's group[9]. The chemical profiling data for *add* RNA were obtained at room temperature with $[Mg^{2+}] = 10$mM. In the absence of the ligand, the population fractions measured by NMR at similar experimental condition were apoA:apoB = 30% : 70% (see Fig. 1c in [10]). In the presence of the ligand, NMR study only gave the population fraction of holo structure at room temperature with $[Mg^{2+}] = 0$mM and $[Mg^{2+}] = 5$mM. Therefore, we did not have the exact population fraction of holo structure for our expected condition (see Fig. 1c in [10]). Fortunately, we can infer that the population fraction of holo at room temperature with $[Mg^{2+}] = 10$mM should be larger than the value with $[Mg^{2+}] = 5$mM ($\sim 35\%$), as the trend reported in [10] is that the population of holo structure increased with the concentration of Mg^{2+}.

With the ligand, SLEQ obtained 9 structures from over 600 candidate structures. We clustered

these structures into clusters apoA+, apoB+ and holo+ based on whether the structure has an empty pocket, no pocket or a constrained pocket. The population fractions of these clusters were apoA+:apoB+:holo+ = $0\% : 54 \pm 0.08\% : 46 \pm 0.08\%$. We also tested the case of only considering apoA, apoB and holo as candidate structures, and the estimated population fractions obtained by SLEQ were apoA+:apoB+:holo+ = $5 \pm 0.08\% : 32 \pm 0.1\% : 63 \pm 0.08\%$. Both population fractions of holo structure were consistent with NMR study ($>\sim 35\%$). Also, the population fraction of apoA measured by NMR was less than 10%, which was consistent with our results. In this case, Cordero *et al.* only reported that the holo structure dominated the ensemble but did not specify its population fraction.

Without the ligand, SLEQ gave 6 structures from more than 600 candidate structures and the population fractions after clustering were apoA+:apoB+=$20 \pm 0.05\% : 80 \pm 0.05\%$. We also tested the case of considering only the apoA and apoB as candidate structures and the population fractions were estimated to be apoA:apoB = $46 \pm 0.1\% : 54 \pm 0.1\%$. These results were consistent with the NMR study (30% : 70%). In this condition, M^2-REEFFIT did not quantify population fractions of complete structure but rather provided the population fractions of helices: P1 = $14 \pm 10\%$, P2 = $37 \pm 15\%$, P3 = $86 \pm 18\%$, P4 = $69 \pm 10\%$, P4B = $36 \pm 6\%$ and P5 = $95 \pm 18\%$. This highlights another difference between the two methods. To make a comparison under this condition, we converted SLEQ's outputs into helix frequencies. The detailed comparison results are shown in in **Supplementary Table 10**. Both methods obtained results that were close to the NMR results in estimating frequencies of about half of the helices and deviated from the NMR results in the remaining half. In addition, SLEQ's error margins were smaller than M2-REEFFITs by $1-2$ orders of magnitude.

3.9.4 16S

For the $126-235$ region of the *E. coli* 16S ribosomal RNA, its crystal structure has a four-way junction including P1a-c, P2a-b, P3 and P4a-b.(see Fig. 2h FWJ-A in [9]). However, conventional SHAPE-directed prediction produce an alternative structure without P2a and P4, and which instead forms alt-P1d and alt-P4 (see Fig. 2h FWJ-B in [9])[20]. M^2-REEFFIT did not recover the SHAPE-based model, but instead predicted a shifted FWJ-A. Similar to the results of *add* RNA as

before, M^2-REEFFIT only reconstructed population fractions of helices. We applied SLEQ to analyze the CE data of wild type and it selected 10 structures from over 600 candidates. We visually inspected these structures and clustered them according to their similarities to the references. Four structures were close to FWJ-A with a sum population fraction 51% and three were close to FWJ-B with a sum population fraction 34%. The remaining three structures were not close to either of the two references and their sum population fraction was 15%. Hence, SLEQ's results support both the crystal structure and conventional SHAPE-directed prediction. To make a direct comparison with M^2-REEFFIT, we summarized population fractions of helices from SLEQ's results (see **Supplementary Table 11**). The respective mean and standard deviation values for SLEQ's results were calculated from 5 runs based on 5 independent simulated read datasets (see **Supplementary Table 12**). As there is no ground truth for population fractions, it is impossible to conclude which approach is more accurate. The results from both methods were highly consistent in population fractions of P1a, P1c P2b, P3 and alt-P4 and did not match well on the remaining 4 helices.

3.10 Performance of SLEQ on the perturbation of important structures

We explored SLEQ's performance when important structures were missing in the candidate set and instead some of their variants were included. In this situation, we wondered whether SLEQ would select these variants as opposed to other random structures. Here, we defined a structure as random if it was missing some key functional motifs that are present in the important structures. To get intuition on this issue, we performed a case study on the co-transcriptional SHAPE-Seq data at the 76nt transcript length in the presence of ligand. As baseline reference, we generated a stochastic Boltzmann ensemble sample as our candidate set and spiked-in the ON structure (henceforth called set S1). In this case, ON and OFF structures were the important structures. SLEQ selected two structures from set S1. Their indices were 36 and 136. As we mentioned in the main text, we clustered selected structures into three clusters: ON+, OFF+, OTHER. If a structure had PK1, it belonged to ON+. If a structure had both P1 and terminator motifs, it belonged to OFF+. If a structure neither belonged to ON+ or OFF+, it belonged to OTHER. We did not require P3 as a necessary motif for the OFF+ cluster. This was because when the transcript length was not long enough to form a CT, there were many nucleotides between P1 and the terminator, which rendered

it unstable to form a small P3 helix and retained many other nucleotides unconstrained. According to this criterion, we found that fold 36 belongs to the OFF+ cluster and fold 136 belongs to the ON+ cluster. Relative abundances were ON+:OFF+= 39 : 61.

To mimic the scenario in which important structures are missing but their variants are included, we perturbed set S1 by incrementally removing base pairs from functional motifs of important structures so that these important structures were replaced by their variants in the candidate set. In this study, we perturbed the PK1, LRIs and PT motifs in ON and P1 and P3 motifs in OFF, respectively (see **Supplementary Table 13**).

The first perturbed important structure is ON. we gradually removed the base pairs of PT from both ends towards the center in a zipper-like fashion (see **Supplementary Fig. 17**). When the first base pair of PT (A49-U66) was removed, SLEQ selected this perturbed ON and OFF. We further removed another BP from the other end (A55-U60), and SLEQ still selected this perturbed ON and OFF. In the process of divergence with the removal of 1 to 7 BPs until PT completely disappeared, SLEQ robustly selected the OFF and the perturbed ON structures. It never selected a random structure. This is likely because data in the pseudoknot region still directed SLEQ properly in terms of structure identification. However, population fractions were redistributed with increasing divergence between variants and the important structures. Similar results were observed in LRIs perturbation. The third motif we perturbed is the pseudoknot PK1, which is the most important motif in the ON structure. In this perturbation, SLEQ robustly selected the OFF and perturbed ON structures and never selected a random structure as long as no more than 4 out of 6 base pairs were removed. After removing over 4 BPs, SLEQ no longer identified this perturbed ON structure but only selected the OFF structure. This is because at this point, the perturbed ON became very similar to the OFF due to the disappearance of the main difference between them, that is, the PK1 pseudoknot.

Next, we perturbed the other important structure – OFF. Since there were many similar OFF structures in the candidate set, it was difficult to find all of them and diverge each motif. Therefore, we created a new candidate set that included ON, two OFF+ and OTHER structures. We reran SLEQ on this new candidate set and it selected fold 36 and 136 with relative abundances ON:OFF=39 : 61 as before, which means that the baseline reference was unchanged. Similarly, we diverged the P1

helix of both OFF+ structures in a zipper-like fashion. When no more than 3 out of 5 BPs were removed, SLEQ consistently identified the ON and perturbed OFF+ structures, and never selected a random structure. When we removed more BPs, in addition to the ON and perturbed OFF+ structures, SLEQ began selecting an additional random structure (see **Supplementary Fig. 18**). This is because both OFF+ and this random structure have CT, where the main difference between them was in the presence and absence of P1 (hence this structure's classification as random). Once P1 was fully opened, SLEQ no longer identified this variant (i.e., OFF structure with no P1) and selected the same random structure as before. Similar results were observed in the P3 divergence. Here, we did not diverge the terminator as it also existed in OTHER structures.

In conclusion, 24 perturbed candidate sets were generated with 1-7 base-pair deviations from important structures. SLEQ consistently identified variants and rejected random structures as long as no more than 40% of the base pairs in a particular key motif were disrupted. However, variation in structures resulted in gradual re-distribution of population fractions.

3.11 Performance of SLEQ in the case of missing important structures

In the previous section, we found that SLEQ is able to identify variants of important structures when the exact important structures were not included in the candidate set. However, if all important structures and their variants were excluded from the candidate set, SLEQ is no longer able to extract these structures and the reconstruction results were biased. Like other "sample and select" approaches, SLEQ's accurate reconstruction relies on the completeness of the candidate set. Here, completeness means that the candidate set includes all relevant structures rather than the entire set of possible structures. In this section, we propose a preliminary method to help recognize when certain important structures are missing, which may aid users when assessing the completeness of their candidate sets in some cases. Here, we illustrate this approach with three RNAs: BST, *MRPS21* riboSNitch and fluoride riboswitch.

3.11.1 BST

We started from a very simple case – BST RNA. As we described in Section 3.9.2, BST is a 25-nt synthetic RNA which adopts the BST-A and BST-B conformations (see **Supplementary Fig. 16**). Its landscape was studied with mutate-and-map data in [9]. We downloaded this mutate-and-map data from RMDB[12] and extracted the SHAPE-CE reactivity profile of wild type. To mimic the scenario in which important structures were missing from the candidate set, we considered BST-B as the only candidate structure, so that BST-A was missing. This simple case was purposed in order to gain intuition on the method. We compared the reactivity profile we expect to see in this case to BST's real profile and observed mismatches. In **Supplementary Figure 19**, the colored background denotes the expected reactivity profile. According to our knowledge of the reactivity profile, reactivity values of constrained nucleotides in BST-B should be low. These are colored white. Reactivity values of unconstrained nucleotides in BST-B should be high and are colored red. We normalized the real profile using the 2%-8% method[6] and results were overlaid on the background. By visually comparing the expected and real profiles, we observed two mismatched regions in which real values were high while the expected values were low. For example, nucleotides $1-4$, $13-14$ were highly reactive but they were paired in BST-B. This suggested that BST-B alone was unable to explain the real data. Therefore, we inferred that certain important structures containing unpaired nucleotides in some of these sites were missing. Similarly, we obtained results for when BST-A was the only candidate. The comparison of expected and real profiles was shown in **Supplementary Figure 20**. We observed similar mismatches in which real reactivity values at some nucleotides were medium-to-high while they were expected to be low. For example, this is exhibited in nucleotides 9, 13, $18-20$ and 25. This suggested that BST-A alone was not enough to accurately generate the real profile. Hence, we inferred that certain key structures were missing from the candidate set. When both BST-A and BST-B were included in the candidate set, SLEQ estimated population fractions for these two structures to be BST-A:BST-B = 71%:29%. We generated the expected reactivity profile using both structures and their respective population fractions. For a nucleotide that was unpaired in both BST-A and BST-B, it was 100% unpaired and the reactivity should be high. Let η be this high reactivity value. For a nucleotide that was unpaired in BST-A but paired in BST-B, it was 71% unpaired and it was assigned a reactivity

value of 0.71η. For a nucleotide that was paired in BST-A but unpaired in BST-B, it was 29% unpaired and it was assigned a reactivity value of 0.29η. For a nucleotide that was paired in both structures, is was 0% unpaired and its reactivity value was set to 0. This model-based reactivity profile is illustrated with different background colors in **Supplementary Figure 21** and overlaid with real profiles. By comparing the two profiles visually, we observed that most highly reactive regions had a reddish background, indicating an improved match.

3.11.2 Human *MRPS21* riboSNitch

We further illustrated this approach using the human *MRPS21* riboSNitch data. The RNA adopts alleles A and C two conformations. We first generated a Boltzmann ensemble sample of 1000 structures for alleles A and C, respectively and then combined them as the candidate set. To mimic a more realistic scenario of missing important structures from the candidate set, we removed allele A and all its structural variants from the candidate set. SLEQ selected 6, all of which were variants of allele C. We chose the one with the minimum free energy as our representative and sketched the expected profile according to it. We then compared the expected and real profiles as before (see **Supplementary Fig. 22**). Nucleotides marked by blue triangles were highly reactive in the real profile but expected to be low under the model, which indicates the absence of certain important structures from the candidate set. We observed similar mismatches when allele C and all its variants were removed from the candidate set (see **Supplementary Fig. 23**). When using the original candidate set without removing any structures, SLEQ reconstructed population fractions for the alleles as A:C = 61%:39%. We sketched the expected reactivity profile by considering two structures and their respective population fractions and compared it to the real profile (see **Supplementary Fig. 24**). We observed that most highly reactive nucleotides were in reddish regions, which indicates an improved fit.

3.11.3 *crcB* fluoride riboswitch

The third demonstration case is cotrascriptional *crcB* fluoride riboswitch at $76nt$ transcript length in the presence of ligand. For this data, we generated a Boltzmann ensemble sample of 1000 structures

and spiked-in ON+ structures. Based on this candidate set, SLEQ reconstructed ON and OFF structures with population fractions of 39% : 61%. To mimic the scenario in which important structures were missing, we removed all structures containing the PK1 motif (ON+) from the candidate set. Based on this new input set, SLEQ selected two OFF+ structures. The expected reactivity profile based on these two selected OFF+ structures was constructed and compared to the real profile (see **Supplementary Fig. 25a**). We observed two mismatches marked by ovals where real reactivity values (black bars) were medium-to-high but expected to be low. The two dashed lines marked cutoffs for low/medium/high reactivity regimes commonly used in interpreting normalized reactivities. We inferred that certain important structures with unpaired nucleotides in some of these regions were missing. When the ON structure was included in the candidate set, one of the two mismatched regions was fixed. This region is denoted by a rectangle (see **Supplementary Fig. 25b**). However, the other mismatch remained, which may be caused by missing certain unknown structures or may be due to the false positive of high reactivity values.

We observed that neither of these two mismatches were caused by PK1 motif. As PK1 constrained the P1 loop, its absence resulted in an expectation of high reactivity in this region whereas real values were not highly reactive (marked by an arrow in **Supplementary Fig. 25a**). This type of mismatch is different from all the previous mismatches we considered. Previously, we looked at mismatches where real values were high but expected values were low. This is hereafter referred to as a type A mismatch. In contrast, in the arrow-marked region, the real values were low and expected values were high. This mismatch is hereafter referred to as a type B mismatch. Based on our experience with SP data, type B mismatches generally conferred less confidence than type A in revealing gaps between true dynamics and the model. This is because there are factors beyond structural constraints that may suppress reactivity (e.g., solvent accessibility). Furthermore, in this case, the mismatches were not as apparent as in the above examples. This was due to the pairing states of ON and OFF structures being very similar, despite having quite different base pairing patterns. The differences of pairing states between ON and OFF are sites $12 - 16$nts (P1 loop) and $67 - 73$nts. For the P1 loop region, there was less information indicating missing ON structure due to this type B mismatch. The only area that was informative in detecting missing ON structure was $67 - 73$nts, which was consistent with the second fixed red region.

Although this pipeline is able to recognize missing important structures in some cases, it has several apparent limitations: 1) It requires user expertise and currently relies on qualitative inspection. 2) It requires manual iterative refinement of the candidate set by the modeler. 3) It relies on type A mismatches only. 4) It might warrant high data density in order to make robust inferences (i.e., SHAPE is better than DMS as it probes all nucleotides). 5) False alarms of high reactivity values might mislead users in thinking that certain important structures are missing. 6) It is important to normalize reactivity profiles (e.g., using the 2%-8% strategy) to ensure consistency in reactivity classification as low/medium/high. 7) Landscapes with many diverse dominant structures might result in blurring of peaks and valleys data signatures of key individual motifs.

3.12 Performance of SLEQ on different average sequencing depths

To explore the minimal average sequencing (ASD) depth for SLEQ's robust prediction, we applied SLEQ to reconstruct RNA landscapes on four cases (*add* RNA in the absence and presence of ligand, 16S RNA and DMS-MaPseq riboSNitch data) at different levels of ASDs. The ASD was defined by the ratio of read number and RNA length. For the first three cases, we simulated read counts for treated and mock-treated (control) samples from 3 respective real reactivity profiles. We used the same data and simulation technique as in our comparisons with M^2-REEFFIT. This allowed us to bridge the gap between capillary-based and NGS datasets. For each RNA, we simulated 10 different datasets of read counts from experimentally obtained CE reactivity profiles on five ASDs (10, 100, 1000, 10000 and 10000). In total, we generated 150 simulated libraries.

For the 16S RNA, SLEQ robustly selected exactly the same structure for 10 repeated libraries at ASD = 10^5 and estimated respective population fractions with almost 0% standard deviation (see **Supplementary Fig. 26a**). Here, we did not cluster the selected structures. Next, we reduced ASD to 10^4, SLEQ still robustly selected exact the same structures for 10 repeated libraries and the standard deviation of population fractions was less than 1% (see **Supplementary Fig. 26b**). When we further reduced ASD to 10^3, SLEQ began to select additional structures with a small fraction assigned to them (see **Supplementary Fig. 26c**). When ASD was decreased to 10^2, SLEQ selected more additional structures but the maximal standard deviation was less than 7% (see **Supplementary Fig. 26d**). When ASD = 10, SLEQ's reconstruction was no longer as

robust as before and the maximal standard deviation increased to 22% (see **Supplementary Fig. 26e**). For the *add* RNA in the presence of ligand, the overall reconstruction was more robust than the 16S case on various ASDs. When ASD >= 10^2, the standard deviation ranged from 0% to 6% (see **Supplementary Fig. 27a-d**). Even when the ASD was reduced to 10, the maximal standard deviation was 7% (see **Supplementary Fig. 27e**). For *add* RNA in the absence of ligand, the standard deviation of population fractions ranged from 0% to 4% when ASD >= 10^2 (see **Supplementary Fig. 28a-d**). When the ASD was reduced to 10, the maximal standard deviation was 12% (see **Supplementary Fig. 28e**).

To assess the consistency of SLEQ's structure selection and population estimation (referred to as reproducibility) in a more intuitive way, we calculated the Pearson correlation coefficients (PCC) between two simulated libraries at each ASD (see **Supplementary Table 14**). We noted that the minimum of PCCs was consistently > 92% for three RNAs at ASD>= 10^3.

For the fourth case, the original sequencing reads were under modification mode and we could summarize them into truncation mode by only considering the first modification at 3' end of each read and ignoring remaining modifications on the same read. We calculated frequencies of read patterns for two modes from the data. We redrew reads according to these calculated probabilities with the total number of reads ranging from 10^7 to 10^2. The resulting ASDs ranged from $3 * 10^5$ to 3. At each ASD level, 10 read datasets were drawn for both modes and the respective means and standard deviations were calculated over 10 runs (see **Supplementary Table 15**). We noted that the standard deviations of both modes were no more than 2% for the two alleles (mean population fractions of alleles A and C = 61% : 39%) at ASD>= 10^3. Therefore, we recommend an ASD of 10^3 for high quality reconstruction.

3.13 Performance of SLEQ on different Boltzmann ensembles

To explore whether SLEQ is able to robustly reconstruct the RNA landscape on different Boltzmann ensemble samples, we analyzed *crcB* fluoride riboswitch RNA for five transcript lengths (57, 66, 69, 75 and 78) in the absence and presence of ligand. For each transcript length, we generated 10 different Boltzmann ensemble samples of 1000 candidate structures using the ViennaRNA package.

Supplementary Table 16 and 17 summarized all results, which showed that SLEQ is able to robustly reconstruct the RNA landscape using different Boltzmann ensemble samples.

3.14 Comparison of mutation and truncation modes with real data

We compared SLEQ's reconstruction in mutation and truncation modes to explore whether mutation mode will outperform truncation mode at certain average sequencing depths. We performed a case study on real data: DMS-MaPseq riboSNitch. The reasons we chose this dataset were: 1) It had reference structures and population fractions. 2) It was mutation data and hence it was able to be summarized into truncation mode by only considering the first modification at 3' of each read and ignoring the remaining modifications on the same read. The inverse conversion was impossible so we were unable to carry out this comparison using truncation data. To decrease the average sequencing depths, we applied the techniques described in Section 3.12. At each ASD, we summarized SLEQ's reconstruction of the two modes by means and standard deviations over 10 libraries (see **Supplementary Table 15**). We applied an unpaired t-test on standard deviations at various ASDs to assess whether the two modes had significant difference in their performances. The $p-$value of 0.35 showed that in this case study mutation mode did not outperform truncation mode.

4 SUPPLEMENTARY NOTE 1: SIMULATIONS

4.1 Performance as a function of η

We tested the impact of increasing η on the ability of SLEQ to reconstruct the landscape using the preQ_1 RNA as a test case. This is an elegant example to validate SLEQ's functionality since it is a real RNA and its two main structures and their relative abundances have been studied with NMR[7]. We assigned relative abundances of $\rho = 0.2 : 0.8$ for those two structures (see **Supplementary Fig. 29**) and 0 for all others. Increasing η represents an increase in the number of modifications per read, causing patterns with multiple mutations to become more prevalent. Therefore, mutation mode is expected to have more informative equations than truncation mode.

However, in this particular case, both modes predicted the two main structures and estimated their relative abundances accurately. This implies that though the information included in truncation mode was less compared to mutation mode, it was sufficient to accurately recover the ground truth for this example (see **Supplementary Table 18**). We observed that the estimation of η in mutation mode was slightly smaller than in truncation mode, as pattern reduction was implemented in the mutation simulation. However, the difference is not statistically significant (p-value $= 0.84$) and does not affect prediction accuracy. The relative abundances, ρ, estimated in both modes had no significant difference (p-value $= 0.70$).

4.2 Performance as a function of relative abundances

We tested the performance of SLEQ for different relative abundances with preQ_1. The two structures described in the previous section were selected as the ground truth and we set $\eta = 0.05$. **Supplementary Table 19** showed that both modes recovered the ground truth accurately under different relative abundances. For preQ_1, the estimated results in both modes had no significant difference (p-value $= 0.56$). Similar to the previous test, we observed that $\hat{\eta}$s in mutation mode were smaller than truncation mode due to pattern reduction. Interestingly, while $\hat{\eta}_{mutation}$ is significantly smaller than $\hat{\eta}_{truncation}$ (p-value < 0.05), the prediction accuracy was not significantly affected, which suggests that SLEQ was able to reconstruct the landscape under a slightly biased estimation of η.

4.3 Robustness to noise in structure ensemble

We tested SLEQ's ability to predict dominant structures in the presence of many minor co-existing structures with small but non-zero relative abundances. Initialization of parameters is described here: the tested RNA is preQ_1 and $\eta = 0.05$. The two structures shown in **Supplementary Figure 29** were selected as the dominant structures and their corresponding relative abundances were $\rho = 0.2 : 0.7$. Then, we randomly selected 10 minor structures and assigned small relative abundances such that their sum was 0.1. We then ran SLEQ 10 times with different minor structures to validate SLEQ's robustness in the reconstruction. **Supplementary Table 20** shows that SLEQ

can predict dominant structures and estimate their relative abundances correctly in both modes even in the presence of many minor structures.

4.4 Robustness to noise in SP data

We also tested the robustness of SLEQ with different noise levels. To mimic the noisy scenario of SP data, we introduced additional noise resource beyond sampling. We initialized relative abundances with $\rho = (0.8, 0.2)$ for the two structures of preQ$_1$ and set $\eta = 0.05$. Additional noise was added to each pattern as follows: the theoretical probability for each patterns, μ, was calculated as described in Online Methods. We used SNR values of $40, 30, 20$ and $10dB$ and calculated the standard deviation for each pattern, σ, using the relationship SNR $= \mu/\sigma$. Noise was then added to each pattern according to a Gaussian distribution with mean 0 and standard deviation set by the σ values. SLEQ reconstructed landscape from these noisy SP data. **Supplementary Table 21** shows that when SNR $= 40, 30, 20dB$, the ground truth was recovered accurately. When the SNR decreases to $10dB$, over the 10 runs, SLEQ was not always able to recover the two dominant structures. (see 10 runs on **Supplementary Table 22**). However, a representative dot plot of base pair probabilities in this case shows that SLEQ is still robust in recovering the main helices (see **Supplementary Fig. 30**). Overall, the performance in mutation mode seems better than truncation mode for this example. We observed that the η estimation in these modes were different, which may account for the increase in estimation bias of the relative abundances for truncation mode. In order to verify our assumption, we fix $\eta = 0.05$ in both modes and implemented the remaining steps as before. The results are summarized in **Supplementary Table 23**. We observed that the performance of truncation mode improved significantly with an unbiased η value, as we expected.

4.5 Additional RNAs

In the previous four sections, we systematically validated SLEQ's capability of reconstructing RNA landscapes using the preQ$_1$ RNA as a case study. Given SLEQ's success on preQ$_1$, we further tested its functionality with other RNAs in a less comprehensive manner in regards to the parameter ranges

examined and the number of runs per test. We started with a longer RNA from the pathogen V. *cholerae*, $VcQrr_3$ (107 nts), which involved in a quorum sensing mechanism. Four structures were selected from [8] as the ground truth (see **Supplementary Fig. 31**). **Supplementary Tables 24, 25, 26 and 27** summarize the results of the tests described in the above sections on $VcQrr_3$. As with $preQ_1$, when the SNR was reduced to $10dB$, it was challenging to recover the ground truth by SLEQ. Nevertheless, mutation mode again outperformed truncation mode. The two modes again produced comparable results when η was fixed. We then chose a randomly generated sequence, M-stable (MST), and an imperfect RNA switch 'Tebowned' (TBWN) from [9] to test SLEQ's reconstruction ability under different noise levels. The selected ground truth structures are shown in **Supplementary Figures 32 and 33** and the results are summarized in **Supplementary Tables 28 and 29**.

Supplementary References

[1] Darty, K., Denise, A. & Ponty, Y. VARNA: Interactive drawing and editing of the RNA secondary structure. *Bioinformatics* **25**, 1974–1975 (2009).

[2] Baker, J. L. *et al.* Widespread genetic switches and toxicity resistance proteins for fluoride. *Science* **335**, 233–235 (2012).

[3] Serganov, A., Polonskaia, A., Phan, A. T., Breaker, R. R. & Patel, D. J. Structural basis for gene regulation by a thiamine pyrophosphate-sensing riboswitch. *Nature* **441**, 1167–1171 (2006).

[4] Serganov, A. *et al.* Structural basis for discriminative regulation of gene expression by adenine- and guanine-sensing mRNAs. *Chemistry and Biology* **11**, 1729–1741 (2004).

[5] Reuter, J. S. & Mathews, D. H. RNAstructure: software for RNA secondary structure prediction and analysis. *BMC Bioinformatics* **11**, 129 (2010).

[6] Low, J. T. & Weeks, K. M. SHAPE-directed RNA secondary structure prediction. *Methods* **52**, 150–158 (2010).

[7] Rieder, U., Kreutz, C. & Micura, R. Folding of a transcriptionally acting $PreQ_1$ riboswitch. *Proceedings of the National Academy of Sciences* **107**, 10804–10809 (2010).

[8] Rogers, E. & Heitsch, C. E. Profiling small RNA reveals multimodal substructural signals in a Boltzmann ensemble. *Nucleic Acids Research* **42**, e171 (2014).

[9] Cordero, P. & Das, R. Rich RNA structure landscapes revealed by mutate-and-map analysis. *PLOS Computational Biology* **11**, 1–24 (2015).

[10] Reining, A. *et al.* Three-state mechanism couples ligand and temperature sensing in riboswitches. *Nature* **499**, 355–359 (2013).

[11] Lorenz, R. *et al.* ViennaRNA package 2.0. *Algorithms for Molecular Biology* **6**, 26 (2011).

[12] Cordero, P., Lucks, J. B. & Das, R. An RNA Mapping DataBase for curating RNA structure mapping experiments. *Bioinformatics* **28**, 3006–3008 (2012).

[13] Martin, M. Cutadapt removes adapter sequences from high-throughput sequencing reads. *EMBnet. journal* **17**, 10–12 (2011).

[14] Langmead, B. & Salzberg, S. L. Fast gapped-read alignment with Bowtie 2. *Nature Methods* **9**, 357–359 (2012).

[15] Zhao, B., Guffy, S. L., Williams, B. & Zhang, Q. An excited state underlies gene regulation of a transcriptional riboswitch. *Nature Chemical Biology* **13**, 968–974 (2017).

[16] Watters, K. E., Strobel, E. J., Yu, A. M., Lis, J. T. & Lucks, J. B. Cotranscriptional folding of a riboswitch at nucleotide resolution. *Nature Structural Molecular Biology* **23**, 1124–1131 (2016).

[17] Watters, K. E., Angela, M. Y., Strobel, E. J., Settle, A. H. & Lucks, J. B. Characterizing RNA structures in vitro and *in vivo* with selective 2'-hydroxyl acylation analyzed by primer extension sequencing (SHAPE-Seq). *Methods* **103**, 34–48 (2016).

[18] Aviran, S., Lucks, J. B. & Pachter, L. RNA structure characterization from chemical mapping experiments. In *49th Annual Allerton Conference on Communication, Control, and Computing (Allerton)*, 1743–1750 (IEEE, 2011).

[19] Aviran, S. & Pachter, L. Rational experiment design for sequencing-based RNA structure mapping. *RNA* **20**, 1864–1877 (2014).

[20] Deigan, K. E., Li, T. W., Mathews, D. H. & Weeks, K. M. Accurate SHAPE-directed RNA structure determination. *Proceedings of the National Academy of Sciences* **106**, 97–102 (2009).
